# Bias-Aware Detection Limits, Calibration Transfer and Matched-Control Robustness Assessment in Dual-Parameter Photonic Refractive-Index and Temperature Sensing: A Coupled Interface-Mode Multilayer Case Study

**DOI:** 10.3390/s26185955

**Published:** 2026-09-20

**Authors:** Agah Oktay Ertay, Muhammed Mustafa Ertay

**Affiliations:** 1 Faculty of Engineering and Architecture, Department of Electrical and Electronics Engineering, Erzincan Binali Yildirim University, Yalnizbag Campus, Erzincan 24002, Türkiye; 2 Faculty of Engineering, Department of Electrical and Electronics Engineering, Duzce University, Konuralp Campus, Duzce 81620, Türkiye; mustafaertay@duzce.edu.tr

**Keywords:** bias-aware detection limit, calibration transfer, dual-parameter sensing, fabrication tolerance, matched controls, nonlinear parameter retrieval, optical sensors, probability of detection, refractive-index sensing, thermo-optic sensing

## Abstract

Dual-parameter photonic sensors are usually reported through a nominal sensitivity and one detection limit, without stating which statistic that limit is or whether it survives transfer between devices. This computational study supplies that evaluation for one structure, a 36-layer one-dimensional multilayer read in transmission, whose Zak-phase-distinct TiO_2_/SiO_2_ photonic-crystal sections enclose a 600 nm analyte cavity and a 500 nm thermo-optic reference cavity, each carrying a 5 nm ITO/5 nm TiO_2_ nanolaminate insert. Two coupled interface resonances at 1517 and 1651 nm, with loaded *Q* of 232 and 208 and refractive-index (RI) sensitivities of 90.71 and 329.41 nm/RIU, are inverted by a bounded nonlinear calibration to $2.43\times 10^{-5}$ RIU and 0.155 °C; the temperature channel reports the device temperature. Probability-of-detection limits at 1% false alarm and 95% detection are $4.36\times 10^{-5}$ RIU and 0.123 °C; they are set by the calibration standards and the wavelength reference, not by the linewidth. Transferring one calibration between devices worsens them 81-fold and 203-fold; a three-point per-device correction removes 84–93% of that loss. A trivial control matched on wavelength, *Q*, transmission, thickness and RI sensitivity shows no topological robustness advantage. Applied to the design itself, the same evaluation shows that the modes are cavity-selected, that hyperbolicity brings no benefit, and that the nanolaminate-free stack is preferred.

## 1. Introduction

Resonant optical sensors are widely investigated because they can provide label-free detection, compact integration, remote interrogation, and strong sensitivity to changes in the optical properties of a surrounding medium [1,2,3,4,5]. Refractive index (RI) and temperature are especially relevant in biochemical analysis, environmental monitoring, process control, and integrated photonics. They are also intrinsically coupled: temperature changes the RI of the analyte, the optical constants of the device materials, and, to a lesser extent, the physical layer dimensions. A resonance shift assigned to an analyte-induced RI change can therefore contain a thermo-optic contribution. Because one spectral observable cannot generally separate two unknown perturbations, simultaneous RI and temperature measurement requires at least two independent channels with sufficiently different response vectors [6,7].

Dual-parameter sensors have been implemented using cascaded Fabry–Pérot cavities, dual-polarization microrings, coupled photonic-crystal cavities, photonic-crystal nanobeams, coupled slabs, and dual quasi-bound states in the continuum [6,7,8,9,10,11,12,13]. Neural-network-assisted nanobeam design has also been explored as an alternative to purely manual modal optimization [14]. These studies establish the feasibility of retrieving RI and temperature from two resonances, most often through a local linear sensitivity matrix. However, the inversion becomes noise-sensitive when the two response vectors are nearly parallel; a local linear model can become biased over a finite two-dimensional operating range; and device-to-device variation can move the sensor away from its nominal calibration. Sensitivity, quality factor, figure of merit, and a detection limit derived only from an assumed wavelength resolution do not fully describe detection performance either. Statistically defensible limits distinguish decision and detection thresholds and account for false-alarm and missed-detection probabilities and calibration uncertainty [15,16], and can be extended to systematic bias.

Multilayer thin-film stacks are an older route to the same measurement, and one with experimental support. A few films deposited at the tip of an optical fiber form coupled Fabry–Pérot cavities whose reflection spectrum depends on the index of the exit medium and, when at least one film is thermo-sensitive, on temperature. Vargas-Rodriguez et al. modeled such a three-layer filter, an antireflection coating, a cyanoacrylate polymer and a silicon layer, by summing the multiple reflections at its interfaces, fabricated it, and recovered index and temperature from measured spectra by decomposing them into intrinsic mode functions, which widened the usable range beyond what a linear sensitivity matrix allows [17]. The same group later replaced the sensitivity matrix by a multiple-regression model over 27 spectral features, again validated on a fabricated filter [18]. Two points follow that the present paper takes as given: the forward model of a thin-film stack is settled physics, and the difficulty of dual-parameter sensing lies in the inversion and its calibration rather than in producing two spectral features. What these works, and the resonant designs above, do not report is the statistical definition of the detection limit, the cost of transferring a calibration between nominally identical devices, and a control against which a claimed structural advantage can be tested. Those are the quantities this paper adds.

Topological photonics offers a different route to spectrally isolated and spatially localized optical states by transferring geometric phases, bulk invariants, and bulk–boundary correspondence to photonic systems [19,20,21]. In an inversion-symmetric one-dimensional photonic crystal, the Zak phase of an isolated band is quantized and can be inferred from parity eigenvalues at inversion-invariant momenta. The corresponding surface impedance or reflection phase links the bulk geometric phase to the presence of an interface state inside a common band gap [22,23,24]; optical Shockley-like surface states in a band-inverted photonic superlattice were the experimental precursor to this picture [25]. The band-gap edges that this argument refers to can be obtained from the Bloch trace of the period transfer matrix, as they are here, or equivalently from generalized-scattering-matrix formulations in which auxiliary functions constructed from the scattering matrix of a single period determine the band-gap characteristics of the periodic structure [26]. This mechanism has motivated refractometric and biosensing structures based on one-dimensional multilayers [27], while the same bulk–boundary reasoning applied to the valley degree of freedom has produced silicon-chip and terahertz sensors built on valley photonic crystals [28,29,30]. Recent studies have further considered topological temperature sensing and dual-parameter RI–temperature detection [31,32].

The phrase “topological protection” nevertheless requires a restricted and testable interpretation in sensing. A useful sensor must respond to its target perturbation, whereas topological robustness applies only to disturbance classes that preserve the symmetry and separation mechanisms supporting the state. Reciprocal photonic topological modes are not universally immune to sharp defects, intervalley scattering, radiation loss, or distributed fabrication disorder [33,34,35]. Rosiek et al., for example, measured strong coherent backscattering and Anderson localization in valley-Hall interface modes under realistic nanoscale disorder [36]. A topological sensing advantage should therefore be evaluated against a trivial control exposed to the same disorder realization and compared under explicitly stated matching conditions. Otherwise, differences in resonance wavelength, linewidth or quality factor, optical access, field overlap, or sensitivity can be mistaken for an effect of topology.

Coupled topological interface states provide two channels within one multilayer architecture. When two localized states are brought into evanescent contact, their degeneracy is lifted, and symmetric- and antisymmetric-like resonances emerge. Their splitting and field distributions are governed by interface separation, spectral detuning, and termination or cap-layer symmetry [37,38]. These degrees of freedom can be used to give one hybrid mode stronger overlap with an analyte region and the other stronger overlap with a thermo-optic reference region. The existence of two topological resonances alone, however, does not guarantee stable parameter separation: modal sensitivities, matrix conditioning, nonlinear calibration, and uncertainty propagation must be considered together. Recent reviews identify multiparameter operation, scalable, defect-free fabrication, and experimental validation as continuing challenges for topological photonic sensors [39].

Hyperbolic metamaterials (HMMs) provide a complementary means of engineering these coupled responses. Their principal permittivity components have opposite signs over a designated spectral range, producing hyperbolic dispersion, access to large-wavevector states and strong control over confinement [40,41], and nanorod and multilayer HMMs have accordingly been studied for refractometric and biomolecular sensing, where material choice and filling fraction tune modal overlap, resonance position and loss [42,43,44,45,46]. HMM-containing one-dimensional photonic-crystal heterostructures can also support topological interface states with reduced angular dependence [47]. No bulk hyperbolic metamaterial is claimed here: a single ultrathin ITO/TiO_2_ constituent pair is not a bulk HMM with a developed high-*k* mode continuum. The nanolaminate is instead designed within a hyperbolic-effective-medium framework so that its principal effective-permittivity components carry opposite signs at the operating wavelengths, and that anisotropy is used to redistribute the fields of the two coupled interface modes: the layer was introduced to make the analyte and thermo-optic overlaps of the two modes more distinct, not to maximize a single RI sensitivity. Section 3.3 audits whether it succeeds and finds that it does not. Measured against the same stack without it, the layer lowers loaded *Q*, peak transmittance, the figure of merit, the determinant of the sensitivity matrix and the specification yield, and leaves the inverse problem worse conditioned. It is retained because the reported design is the one every other number in this paper refers to, and the audit is reported as a negative result rather than removed from view; on the strength of that audit, the nanolaminate-free stack is recommended for fabrication (Section 3.3), and the hyperbolicity criterion is withdrawn from the design specification. The EMT–explicit spectral agreement of Section 3.2 validates the effective description at the design wavelengths and is not evidence for high-*k* mode density or a bulk hyperbolic density of states.

The literature has separately established dual-resonance RI–temperature sensing, topological interface-state formation, coupled-state hybridization, and HMM-enhanced confinement. A unified analysis that combines these mechanisms with nonlinear two-dimensional calibration, covariance- and bias-aware detection limits, matched topological–trivial controls, high-sample fabrication uncertainty, and independent electromagnetic and transport validation has remained limited [9,14,32,37,39]. More fundamentally, it has not been systematically established whether a topological sensor has an intrinsic robustness advantage or whether an observed benefit depends on the operating point and on which properties of the trivial reference are matched. This distinction is essential because persistence of an interface resonance alone does not demonstrate improved sensing performance.

This work applies that evaluation to one structure: an ITO/TiO_2_-nanolaminate-loaded one-dimensional interface-mode multilayer that supports two coupled interface modes for simultaneous RI and temperature measurement, with its nanolaminate-free variant identified as the configuration to fabricate. The main contribution is not the multilayer or its forward model. Both are standard, and Section 4.2 shows that several published designs beat this one on nominal sensitivity and quality factor. The contribution is a complete, statistically defined account of what such a device can measure, applied to one frozen design and reported in full, including the parts that come out against the design. Three results carry it. First, the detection limit is computed as a statistic rather than read from an assumed resolution divided by a sensitivity: a decision limit, a bias-aware minimum detectable change and a probability-of-detection limit at declared false-alarm and detection rates are propagated from a correlated wavelength covariance through a bounded nonlinear two-dimensional calibration. Varying every input of that budget over two decades shows that the limits are set by the calibration standards and the wavelength reference, not by the resonance linewidth. Second, calibration is treated as a property of the individual device. Transferring one nominal calibration to perturbed devices costs factors of 81 and 203, a per-device correction from three reference measurements recovers 84–93% of that loss, and the fabrication variations that make the correction necessary are identified. Third, the topological claim that such designs usually assert is tested against trivial controls exposed to the same disorder and matched on the observables that determine performance. Against the control matched on wavelength, loaded *Q*, peak transmission, thickness and RI sensitivity at once, the topological design is 4.4 percentage points worse with an interval excluding zero, so no robustness benefit attributable to topology is isolated.

Based on those three results, the paper audits the structural claims the design is built on and reports each audit whichever way it comes out. The sensing modes are cavity-selected resonances of the topological junction rather than the continuation of its bare-interface states; the ITO/TiO_2_ nanolaminate lowers every metric measured, so the nanolaminate-free stack is recommended for fabrication; and the hyperbolicity condition acts on no observable at normal incidence and brings no benefit at oblique incidence, so it is withdrawn from the specification. First-order perturbation theory ties the sensitivity matrix to the modal overlaps, which turns the separability of the two channels into a design rule, the difference between the modes’ reference-to-analyte overlap ratios, and makes the reference-coating thermo-optic coefficient a materials specification. A predeclared N=1000 Monte Carlo gives a 95.9% specification yield with its Wilson interval, the angular and polarization requirements of the planar readout are stated as specifications, and the optical model is checked against five independent numerical formulations while the transport model is solved by finite elements [48].

Section 2 presents the architecture and methods, Section 3 reports the optical and sensing results, Section 4 discusses the robustness interpretation and limitations, and Section 5 summarizes the conclusions.

## 2. Materials and Methods

This section defines the frozen sensor geometry, material descriptions, electromagnetic formulation, calibration and uncertainty procedures, robust-design protocol, and independent numerical validation models. All publication calculations use one frozen design, defined by the geometry and material tables below. The numerical values reported in this section are therefore input parameters, declared analysis settings, or acceptance criteria. Calculated band invariants, resonance metrics, sensitivities, retrieval errors, detection limits, yields, and cross-solver discrepancies are reserved for Section 3. The study is entirely numerical; agreement among independent solvers is not described as experimental validation.

### 2.1. Proposed Sensor Architecture

The proposed sensor is a finite one-dimensional multilayer normal to the *z*-axis. Its compact layer sequence is

(1) $${\left(U_{1}\right)}^{N_{\mathrm{out}}}\;|\;H_{L}\;|\;A_{n}\;|\;{\left(U_{2}\right)}^{N_{\mathrm{mid}}}\;|\;A_{r}\;\left|\;H_{R}\;\right|\;{\left(U_{1}\right)}^{N_{\mathrm{out}}},$$

where $U_{1}=\left(B/2\right)A\left(B/2\right)$ and $U_{2}=\left(A/2\right)B\left(A/2\right)$ are inversion-symmetric binary photonic-crystal unit cells. Layer *A* is TiO_2_, layer *B* is SiO_2_, $H_{L}$ and $H_{R}$ are HMM-design nanolaminate inserts, referred to below simply as nanolaminate inserts, $A_{n}$ is the analyte region, and $A_{r}$ is the thermo-optic reference region. The unit cells are related by a translation and consequently have the same infinite-crystal dispersion. Their inversion-center convention and finite-stack termination differ, however, so their parity ordering, Zak-phase description, and boundary reflection phase must be evaluated together. A topological distinction is therefore not inferred from the labels $U_{1}$ and $U_{2}$ alone; it is tested through the bulk bands, parity eigenvalues, Zak phases, reflection phases, and localized finite-stack states [21,23,24].

The analyte region is exposed to the sample and responds directly to its RI. The reference region is isolated from analyte exchange in the model and is assigned a comparatively large negative thermo-optic coefficient. The two regions create nearby localization centers separated by the finite $U_{2}^{N_{\mathrm{mid}}}$ section. Evanescent overlap between the corresponding interface states produces two hybrid resonances rather than two independent defect peaks. The nanolaminate inserts modify the phase accumulation, confinement, loss, and relative field overlap of both hybrid states. Their purpose is consequently to improve the distinctness of the two response vectors rather than to maximize the RI sensitivity of one resonance. Section 3.3 tests that purpose directly by deleting the inserts and finds that it is not met: the same stack without them is better on every metric measured, including the probability-of-detection limits and the calibration-transfer penalty so the nanolaminate-free configuration is the one recommended for fabrication. The loaded stack is nevertheless kept as the analyzed case study throughout, because every downstream statistic, matched control and cross-solver validation in this paper refers to it, and the detection-limit and calibration-transfer framework that is the paper’s subject does not depend on which of the two stacks it is applied to.

**Figure 1:**
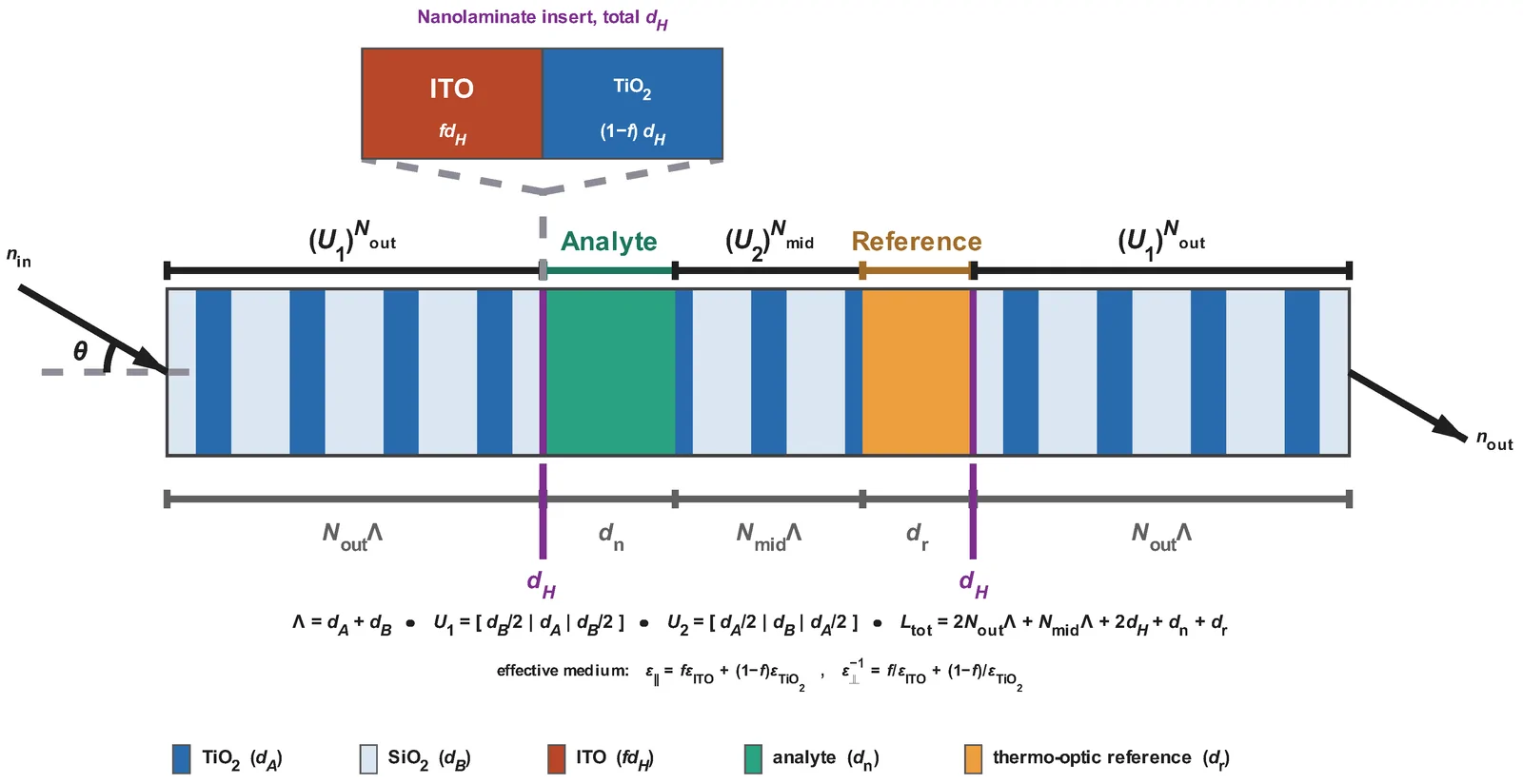
Frozen physical architecture of the ITO/TiO_2_-nanolaminate-loaded one-dimensional interface-mode sensor, the reference configuration analysed throughout; the variant recommended for fabrication in Section 3.3 omits the two inserts and is otherwise identical. The analyte and thermo-optic reference regions form two localization centers, while the finite central photonic section controls their coupling. Each nanolaminate insert is resolved as an explicit 5nm ITO/5nm TiO_2_ pair.

The frozen geometry is specified by the explicit layer table (Table 1) and drawn in Figure 1. Each nominally 10 nm nanolaminate insert is represented by one 5 nm ITO layer and one 5 nm TiO_2_ layer, so the physical reference is a 36-layer isotropic constituent stack and not a fabricated homogeneous anisotropic film. The two outer $U_{1}$ sections act as finite reflectors, the shorter $U_{2}$ section separates the analyte and reference localization centers and sets their evanescent coupling, and each cavity sits next to one insert so that its perturbation weights one hybrid mode. The incidence and exit arrows define the illumination convention used for all scattering calculations, and the inset shows that the optical-axis response used during effective-medium optimization comes from an ITO/TiO_2_ sub-period rather than from an intrinsically anisotropic material. Read quantitatively, the stack is 5407.40 nm in 36 layers: 18 low-index *B* layers totaling 2672.40 nm, 12 high-index *A* layers totaling 1615.00 nm, the 600 nm analyte domain, the 500 nm reference coating and the two inserts. The nanolaminate is therefore 20 nm of material, 0.37% of the stack, placed where the two localization centers already sit; the modes peak at z=3562 and 1911 nm for modes 1 and 2, inside the two shaded regions of the figure, so the drawn layout and the field distribution of Section 3.5 refer to the same positions.

The structure is interrogated by a plane wave. Normal incidence is used for the primary calibration, and TE/TM angle sweeps are used as a robustness audit. Complex reflection and transmission amplitudes are retained. The power coefficients are

(2) R=|S11|2,T=Re(Yout)Re(Yin)|S21|2,

where $Y_{\mathrm{in}}$ and $Y_{\mathrm{out}}$ are the polarization-dependent admittances of the incident and exit media. The admittance prefactor prevents an amplitude coefficient from being interpreted as transmitted power when the external media differ. Loaded quality factors are obtained from $Q_{\nu }=\lambda_{\nu }/{\mathrm{FWHM}}_{\nu }$. Throughout RI, temperature, angle, and fabrication sweeps, the two resonances are tracked by a combined cost based on spectral proximity, peak prominence, linewidth, transmission, and field-profile overlap. This continuous labeling prevents a simple wavelength sort from exchanging mode identities near an avoided crossing or a degraded peak.

### 2.2. Optical Material Models

The dispersion forms for both dielectrics and the reference coating, the ITO Drude parameterisation with its measured-film cross-checks, and the effective-medium treatment of the nanolaminate are given in full in Section S1.

### 2.3. Theoretical Framework

#### 2.3.1. Electromagnetic Wave Propagation in Anisotropic Layers

The structure is linear, reciprocal, nonmagnetic, and laterally uniform in its primary one-dimensional representation. With the $e^{-i\omega t}$ convention, the HMM tensor is

(3) $$\varepsilon =\mathrm{diag}(\varepsilon_{\|},\varepsilon_{\|},\varepsilon_{⊥}),$$

where the *z*-axis is normal to the interfaces. The conserved tangential wave number is $k_{x}=k_{0}n_{\mathrm{in}}\sin\theta$, with $k_{0}=\omega /c$. TE polarization is described by $E_{y}$, whereas TM polarization is described by $H_{y}$:

(4) $$\begin{array}{cccc} \frac{d^{2}E_{y}}{dz^{2}}+q_{\mathrm{TE}}^{2}E_{y} & =0,\; & q_{\mathrm{TE}}^{2} & =\varepsilon_{\|}k_{0}^{2}-k_{x}^{2}, \end{array}$$

(5) $$\begin{array}{cccc} \frac{d^{2}H_{y}}{dz^{2}}+q_{\mathrm{TM}}^{2}H_{y} & =0,\; & q_{\mathrm{TM}}^{2} & =\varepsilon_{\|}k_{0}^{2}-\frac{\varepsilon_{\|}}{\varepsilon_{⊥}}k_{x}^{2}. \end{array}$$

The passive square-root branch is selected continuously so that lossy or evanescent fields decay away from their excitation interface. The corresponding tangential admittances are

(6) $$Y_{\mathrm{TE}}=\frac{q_{\mathrm{TE}}}{\omega \mu_{0}},\;Y_{\mathrm{TM}}=\frac{\omega \varepsilon_{0}\varepsilon_{\|}}{q_{\mathrm{TM}}}.$$

Equations (4)–(6) make the incidence-angle dependence explicit. At normal incidence, the isotropic portions give identical TE and TM spectra, whereas at oblique incidence, TM waves sample both principal HMM components. The TE/TM interface algebra follows from Equations (4)–(6) with passive branch selection applied to every square root.

Two cascade implementations are maintained, and their roles are distinct. Production calculations, meaning every spectrum, resonance metric, sensitivity matrix, calibration surface and Monte Carlo ensemble reported here, use the characteristic-matrix (transfer-matrix) cascade, which is the faster route for the millions of spectral evaluations these studies require. A stabilized Redheffer scattering-matrix recursion, which keeps the evanescent orders bounded in thick stopband layers, is retained as an independent numerical-conditioning audit rather than as the production solver: over the operating band the two agree to a transmittance RMSE of $2.5\times 10^{-15}$, so the choice of cascade does not affect any reported quantity. The recursion, the conditioning audit and the loss-scale and period range within which unrestricted transfer-matrix multiplication remains safe are in Section S2.1.

#### 2.3.2. Hyperbolic Metamaterial Effective Permittivity

For ITO filling fraction $f=d_{\mathrm{ITO}}/\left(d_{\mathrm{ITO}}+d_{{\mathrm{TiO}}_{2},H}\right)$, local layered-medium theory gives [40,41]

(7) $$\varepsilon_{\|}=f\varepsilon_{\mathrm{ITO}}+\left(1-f\right)\varepsilon_{{\mathrm{TiO}}_{2}},$$

(8) $$\varepsilon_{⊥}={\left(\frac{f}{\varepsilon_{\mathrm{ITO}}}+\frac{1-f}{\varepsilon_{{\mathrm{TiO}}_{2}}}\right)}^{-1}.$$

Throughout this paper, ‖ denotes the layer plane and ⊥ the layer normal. The review cited above labels the same two components by their orientation relative to the anisotropy axis, so its subscripts are interchanged with respect to the convention used here; the two expressions are the same. The medium is hyperbolic at a given wavelength when

(9) Re(ε‖)Re(ε⊥)<0.

Type I corresponds to Re(ε‖)>0 and Re(ε⊥)<0; Type II has the opposite signs. Classification is evaluated separately at both resonance wavelengths and for every ITO data set. Absorption is never removed from the forward calculation, even when the sign criterion is discussed using real parts.

For TM polarization, the extraordinary-wave dispersion can also be written

(10) $$\frac{k_{x}^{2}}{\varepsilon_{⊥}}+\frac{k_{z}^{2}}{\varepsilon_{\|}}=k_{0}^{2}.$$

Opposite-sign tensor components open a large-wavevector sector and provide a mechanism for modifying confinement and coupling. Whether this mechanism is accurately represented by the local tensor is tested against the explicit bilayer rather than assumed.

The Bloch dispersion, the parity-based Zak phase and the reflection-phase criterion that together establish the band-inversion between the two half-crystals are set out in Section S3.

#### 2.3.3. Coupled Interface Resonances of a Topological Junction

The two localization centers are interpreted using a non-Hermitian two-mode Hamiltonian,

(11) $$H=\left[\begin{array}{cc} {\tilde{\omega }}_{a} & \kappa \\ \kappa & {\tilde{\omega }}_{r} \end{array}\right],\;{\tilde{\omega }}_{j}=\omega_{j}-i\gamma_{j},$$

where $\omega_{a}$ and $\omega_{r}$ are the uncoupled analyte- and reference-associated frequencies, $\gamma_{j}$ contains radiative and absorptive decay, and κ is the coupling coefficient. The hybrid eigenfrequencies are

(12) $${\tilde{\omega }}_{\pm }=\frac{{\tilde{\omega }}_{a}+{\tilde{\omega }}_{r}}{2}\pm \sqrt{{\left(\frac{{\tilde{\omega }}_{a}-{\tilde{\omega }}_{r}}{2}\right)}^{2}+\kappa^{2}}.$$

Near zero detuning, the eigenvectors are symmetric- and antisymmetric-like superpositions. Their splitting depends on interface separation, detuning, and termination [37,38], and here also on the phase accumulated in the anisotropic nanolaminate. Because the hybrid eigenvectors have different field weights in $A_{n}$ and $A_{r}$, they provide two distinct sensing channels. Temporal coupled-mode theory is used only as a physical interpretation of port access and weak line-shape asymmetry [49], and, in its multimode form, of the splitting of the two coupled interface states [50]; all quantitative spectra are obtained from the full dispersive multilayer calculation.

#### 2.3.4. Perturbation Theory for RI and Temperature Sensitivities

For a weak, nondispersive dielectric perturbation in a closed or weakly radiating system, the first-order shift of mode ν is

(13) $$\frac{\Delta \omega_{\nu }}{\omega_{\nu }}≃-\frac{1}{2}\frac{\int_{V}E_{\nu }^{*}\;\;\cdot \;\Delta \varepsilon \;E_{\nu }\;dV}{\int_{V}E_{\nu }^{*}\;\;\cdot \;\varepsilon \;E_{\nu }\;dV}.$$

An RI perturbation is concentrated in the analyte region, so its effect increases with the electric-energy overlap of that region. Temperature produces a distributed perturbation through the thermo-optic coefficients of the dielectric, reference, and ITO regions, together with thermal expansion of the interfaces. Moving high-contrast boundaries require the appropriate boundary perturbation terms rather than a naive volume replacement [51].

Equation (13) is consequently used to explain the physical role of modal overlap, not as the final numerical sensitivity formula. The proposed stack contains lossy, dispersive ITO and moving interfaces; quantitative RI and temperature derivatives are obtained by re-solving the complete structure at perturbed parameter values and continuously tracking the same two modes. The resulting sensitivities and field-overlap interpretation are presented in Section 3.5.

#### 2.3.5. Dual-Parameter Sensitivity Matrix

The linearization of the two resonance surfaces about $\left(n_{0},T_{0}\right)$ gives

(14) $$\Delta \lambda =S\Delta p,\;\left[\begin{array}{c} \Delta \lambda_{1}\\ \Delta \lambda_{2} \end{array}\right]=\left[\begin{array}{cc} S_{n,1} & S_{T,1}\\ S_{n,2} & S_{T,2} \end{array}\right]\left[\begin{array}{c} \Delta n\\ \Delta T \end{array}\right],$$

where $S_{n,\nu }=\partial \lambda_{\nu }/\partial n$, and $S_{T,\nu }=\partial \lambda_{\nu }/\partial T$. Local separation requires

(15) $$\det\left(S\right)=S_{n,1}S_{T,2}-S_{T,1}S_{n,2}\neq 0.$$

A determinant approaching zero indicates nearly parallel modal response vectors and an increasingly noise-sensitive inverse problem. Because RI and temperature have different units and practical excursion scales, the raw condition number is not physically interpretable. Conditioning is evaluated from

(16) $$\tilde{S}=D_{\lambda }^{-1}SD_{p},\;\kappa_{2}\left(\tilde{S}\right)=\frac{\sigma_{\max}\left(\tilde{S}\right)}{\sigma_{\min}\left(\tilde{S}\right)},$$

where $D_{p}$ contains declared representative RI and temperature excursions and $D_{\lambda }$ defines the wavelength scale. The local matrix is used for conditioning, small-error propagation, and initial guesses; it is not used as the global inverse model over the full domain.

### 2.4. Nonlinear Calibration and Parameter Retrieval

The forward map is defined by two nonlinear surfaces,

(17) $$\lambda_{1}=f_{1}\left(n_{a},T\right),\;\lambda_{2}=f_{2}\left(n_{a},T\right).$$

Using the validated uniaxial EMT representation defined in Section S1.3 of the Supplementary Material, the full complex spectrum is recalculated at every point of the calibration grid $n_{a}=1.33,1.34,\ldots ,1.40$ and T=25,37.5,…,125 °C. The continuously tracked resonance centers form tensor-product surfaces interpolated with a spline inside the calibration bounds. Nearest-bound fallback is disabled as a scientific extrapolation mechanism: solutions outside the declared domain are rejected, not reported as calibrated estimates.

For an observed wavelength vector, $\lambda^{\mathrm{obs}}$, the recovered parameters solve the bounded, covariance-weighted problem

(18) $$\left(\hat{n},\hat{T}\right)=\underset{1.33\leq n\leq 1.40,\;25\leq T\leq 125}{\mathrm{argmin}}{\left[\lambda^{\mathrm{obs}}-f\left(n,T\right)\right]}^{T}\Sigma_{\lambda }^{-1}\left[\lambda^{\mathrm{obs}}-f\left(n,T\right)\right].$$

The nearest calibration-grid point provides the initial estimate, after which bounded nonlinear least squares is run with a maximum of 120 iterations and a function tolerance of $10^{-12}$. An estimate is accepted only if the solver converges, the solution remains within the calibrated domain, the residual meets the declared tolerance, and both observations retain the identity of the calibrated modal pair.

Independent validation uses the interleaved grid $n_{a}=1.335$, 1.345, …, 1.395 and T=31.25, 43.75, …, 118.75 °C, which is not used to build the interpolation surfaces. Eight noisy replicas are generated per validation point using the declared spectral readout model. Retrieval RMSE, bias, failure fraction, boundary behavior, and parity plots are reported in Section 3.6. Since fabrication changes both zero points and response surfaces, the primary performance scenario assumes device-specific zeroing and two-dimensional calibration. A transferred nominal calibration is evaluated separately rather than conflated with per-device performance.

**Figure 2:**
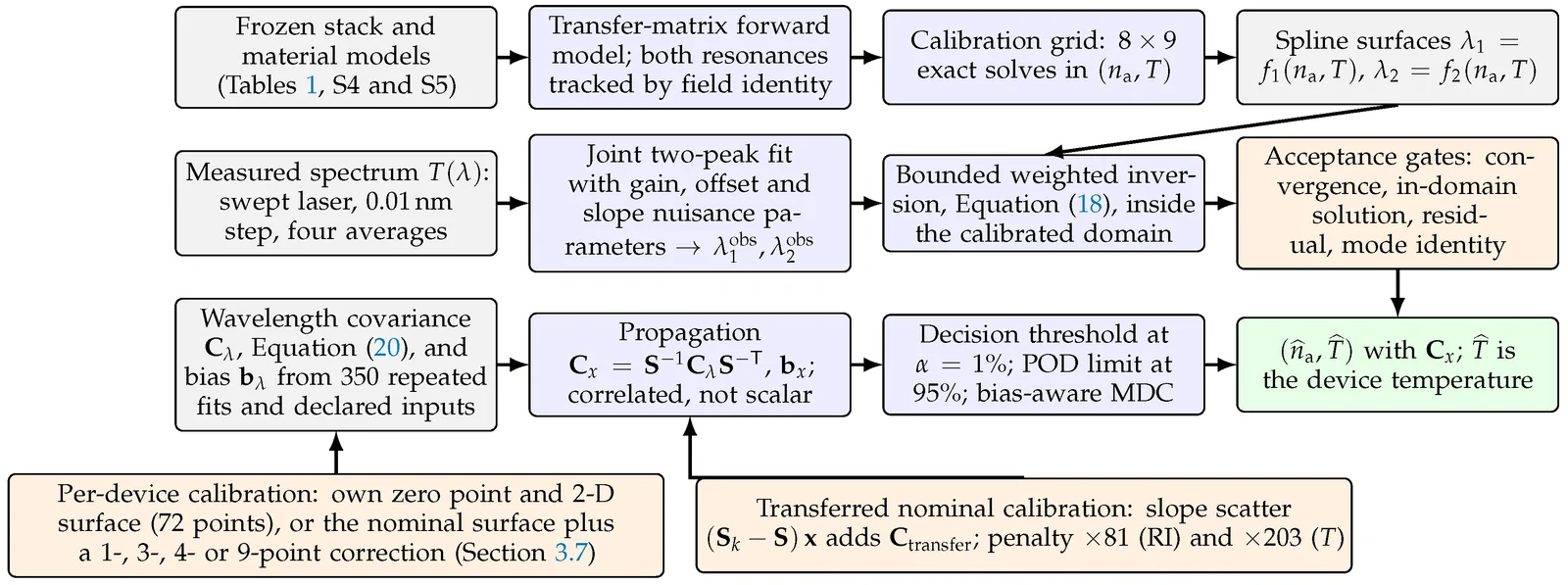
Graphical summary of the sensing and retrieval procedure. Top row: the offline calibration, in which the frozen stack is solved exactly on the 8×9 grid and the two tracked resonance surfaces are interpolated. Second row: the measurement chain, from the sampled transmission spectrum through the joint two-peak fit to the bounded weighted inversion and its acceptance gates. Third row: the uncertainty chain, from the five-component wavelength covariance and its bias through the sensitivity matrix to the decision threshold and the probability-of-detection limit. Bottom row: who owns the calibration, either the individual device or a nominal design transferred to it; the per-device options are compared in Section 3.7. Gray boxes are data, light purple boxes are computations, orange boxes are decisions, and the green box is the reported result.

Two points about that scenario are stated here because they fix what the reported numbers mean. First, the temperature that the inversion returns is the temperature of the multilayer itself: with the analyte thermo-optic coefficient set to zero in the forward model, the reference coating is the only element whose index responds to temperature, so $\hat{T}$ is the device temperature and not an independent measurement of the fluid temperature. Section 3.5 quantifies how the calibration changes when an aqueous analyte with its own thermo-optic coefficient is included. Second, the per-device calibration assumed for the headline limits is a full two-dimensional surface, 72 exact points per device, which is impractical for a fabricated population. Section 3.7 therefore also evaluates reduced per-device protocols in which the nominal surface is kept and only a low-order correction is determined from one, three, four or nine reference measurements on the individual device, the standardization approach used for calibration transfer in chemometrics [52]. Figure 2 summarizes the complete sensing and retrieval chain.

### 2.5. Measurement-Uncertainty and POD–LOD Framework

The measured spectrum is represented by

(19) $$T_{\mathrm{meas}}\left(\lambda \right)=gT_{\mathrm{model}}\left(\lambda ;\lambda_{1},\lambda_{2},\ldots \right)+a_{0}+a_{1}\left(\lambda -\lambda_{c}\right)+\eta \left(\lambda \right),$$

where *g* is an amplitude-gain nuisance parameter, $a_{0}$ and $a_{1}$ are baseline offset and slope, and η is detector noise. The two resonance centers are fitted jointly, so that common wavelength-axis, baseline, temperature, angle, and calibration errors generate nonzero covariance.

The reference instrument model uses a 0.01 nm sampling step, a 0.002 nm fine fit grid, a 0.020 nm instrumental resolution FWHM, a 0.005 nm source linewidth, detector-noise RMS $10^{-3}$ in normalized transmission, and four averaged acquisitions. The covariance components are estimated from 350 repeated spectrum fits and declared wavelength-reference, temperature-stability, angular-repeatability, and calibration-standard uncertainties. These values are simulated instrument specifications, not measurements from a physical prototype.

The instrument model corresponds to swept tunable-laser interrogation of the transmission spectrum, in which the source is stepped across both resonances and the two centers are extracted from the sampled line shapes by a joint fit, the readout used for scanned ring-resonator sensor arrays [53]. The resonance center is recovered far below the linewidth because it is a fit parameter rather than a sampling coordinate. For a peak of full width, Γ, sampled at *N* points inside its width with an amplitude signal-to-noise ratio SNR, the Cramér–Rao bound on the center is of order $\Gamma /(\mathrm{SNR}\sqrt{N})$, an order-of-magnitude form stated here for orientation and not taken from a published derivation. The same inverse dependence on signal-to-noise ratio, and the gain from denser sampling of the line, appear in the simulation benchmark of curve-fitting peak trackers for fiber Bragg gratings [54]; White and Fan showed, for extremum tracking of a Lorentzian line, that the resolution of a resonant refractometric sensor is set by amplitude noise (their Monte Carlo scaling is $\sigma \approx \Gamma /(4.5\;{\mathrm{SNR}}^{1/4})$), thermal drift and spectral sampling rather than by the sensitivity or linewidth alone [55]. Here, Γ=6.55 and 7.94 nm, the 0.01 nm step places about 650 and 790 samples inside each linewidth, and the $10^{-3}$ detector noise on a unit-normalized transmission with four averages gives an amplitude signal-to-noise ratio of order $10^{3}$, so the bound is $1.9\times 10^{-4}$ and $1.5\times 10^{-4}$ nm for the two modes. The 350 repeated joint fits return $1.8\times 10^{-4}$ and $1.4\times 10^{-4}$ nm (Figure S16 of the Supplementary Material). The finite instrument resolution enters as a bias rather than as noise: convolving the asymmetric line shapes with the 0.020 nm response displaces the fitted centers by −3.4 and −4.4 pm, and that displacement is carried in the bias-aware minimum detectable change rather than in the covariance. Section 3.6 shows how the reported limits move when every one of these inputs, and a slow drift between calibration and measurement, is varied over two decades, and Section 3.7 does the same for the calibration protocol.

Those five parameters determine $C_{\mathrm{fit}}$ and nothing else, so listing only them would leave the four remaining terms of Equation (20) undeclared, and Section 3.6 shows that those are the terms that set the reported limits: calibration repeatability supplies 93.9% of the index variance and thermal drift 47.0% of the temperature variance. Table 2 therefore gives the input standard deviation of every component, so that a reader can rebuild $C_{\lambda }$ and see which instrument specification each reported limit is a restatement of.

The angular entry needs a specification attached to it. At normal incidence the resonance shift is quadratic in angle, $\Delta \lambda ≃-\lambda_{0}\theta^{2}/\left(2n_{\mathrm{eff}}^{2}\right)$, so a symmetric jitter contributes almost nothing and the angular term is the smallest in the budget; a static misalignment $\theta_{0}$ makes the response locally linear, $d\lambda /d\theta =\lambda_{0}\theta_{0}/n_{\mathrm{eff}}^{2}$, and with $n_{\mathrm{eff}}=1.55$ from the 22.56 nm drift over 15° of Section 3.11 this is 0.020 nm per degree of jitter for every 0.1° of $\theta_{0}$. With the declared 0.020° jitter, the angular term reaches the $1.0\times 10^{-3}$ nm common-mode wavelength reference at about 0.25° of static misalignment; the operating specification adopted here is the more conservative ±0.1°, so that the alignment term stays well below every other entry in the budget. Holding the mean incidence within ±0.1° of normal is therefore a readout requirement of this design.

The conditioning figure quoted throughout uses a declared scaling, without which it is not reproducible. The scaled condition number is $\kappa =\mathrm{cond}(D_{\lambda }^{-1}SD_{p})$ with $D_{\lambda }=I$ in nm and $D_{p}=\mathrm{diag}\left(0.01\;\mathrm{RIU},10\;{}^{\circ}C\right)$, which expresses both parameter axes in units a user would actually resolve.

The wavelength-error covariance is decomposed as

(20) $$C_{\lambda }=C_{\mathrm{fit}}+C_{\mathrm{laser}}+C_{\mathrm{thermal}}+C_{\mathrm{angle}}+C_{\mathrm{cal}}.$$

For local propagation with calibration Jacobian J=∂f/∂(n,T),

(21) $$C_{p}≃{\left(J^{T}C_{\lambda }^{-1}J\right)}^{-1}.$$

The residual wavelength bias $b_{\lambda }$ is estimated from held-out fits and propagated separately as

(22) $$b_{p}≃{\left(J^{T}C_{\lambda }^{-1}J\right)}^{-1}J^{T}C_{\lambda }^{-1}b_{\lambda }.$$

Random precision and systematic bias are not merged into a single standard deviation. This follows the distinction among decision, detection, and quantification limits in classical analytical calibration [15,16].

For a recovered parameter change, $\hat{x}$, a two-sided threshold, $\tau_{x}$, is selected under the null distribution, so that

(23) $$P(|\hat{x}|>\tau_{x}|\Delta x=0)\leq \alpha ,\;\alpha =0.01.$$

The POD-based limit is

(24) $${\mathrm{LOD}}_{\mathrm{POD},x}=\inf\left\{\Delta x>0:P(\hat{x}>\tau_{x}|\Delta x)\geq 0.95\right\}.$$

The detection curves are two-sided Gaussian detection probabilities evaluated on the calibrated parameter covariance, which rests on 350 repeated spectrum generations with joint two-peak fitting propagated to parameter space through the sensitivity matrix. A separate 30,000-draw scalar Monte Carlo confirms that the decision threshold realizes the requested false-alarm rate and that the POD limit is detected with the requested probability; that check is an arithmetic validation and not a model validation, because it draws from the same Gaussian and cannot test whether the Gaussian describes the real fitting residuals. The test that would work is a resampling of the complete forward-plus-fit-plus-inverse chain, which is what the 350 repeated generations behind the covariance provide and what the nonlinear retrieval RMSE reports. Conventional 3σ values and bias-aware minimum detectable changes are retained as secondary metrics; the covariance-based POD limit is the primary LOD. The covariance matrices, bias estimates, false-alarm frequencies, detection probabilities and final limits are reported in Section 3.6.

The design search is described in Section S4: a five-stage hybrid over 76 discrete configurations and ten bounded variables, with every non-dominated candidate recomputed under the full model, and with the predeclared selection rule whose behavior Section 3.8 analyses.

Independent solver configurations, meshes, boundary treatments, the transport–reaction model and the runtime environment are specified in Section S5.

## 3. Results

The results follow the design argument: why two interface modes exist, what the nanolaminate does to them, what survives three structural audits, how the field distribution fixes the sensitivity matrix, and what that matrix delivers in sensing, retrieval, yield and matched-control terms. Figures S1–S3 of the Supplementary Material summarize the same argument in three composite panels.

### 3.1. Topological Band Structure and Coupled Interface Modes

The two translation-related unit cells produced coincident bulk dispersions, as expected, because translating the inversion center cannot change the eigenfrequencies of the infinite crystal. Their parity sequences differ nonetheless. For bands 1–4 the parity pairs (ξ(0),ξ(π/Λ)) are (+,−), (−,+), (+,+) and (+,−) for $U_{1}$, giving Zak phases π, π, 0 and π, while the $U_{2}$ pairs (+,+), (−,−), (+,−) and (+,+) give 0, 0, π and 0. The first common gap is therefore bounded by bands carrying different cumulative geometric phases, and the distinction comes from the inversion-center and termination convention rather than from any difference between the bulk spectra. The terminated crystals accordingly carry opposite reflection-phase branches inside the common stopband, with the $U_{1}$ phase staying negative and the $U_{2}$ phase remaining positive over most of the interval. Their sum, with the finite central-section phase, satisfies the round-trip condition of Section S3.3 of the Supplementary Material at two wavelengths, so the finite device supports two coupled interface resonances: 1516.790 and 1651.200 nm, peak transmittances 0.50994 and 0.67433, FWHM 6.5463 and 7.9419 nm, loaded quality factors 231.70 and 207.91. No single plot is treated as proof of two finite, lossy resonances; the bulk-band, parity, reflection-phase and field-localization evidence support them jointly [19,20,21,23,24].

**Figure 3:**
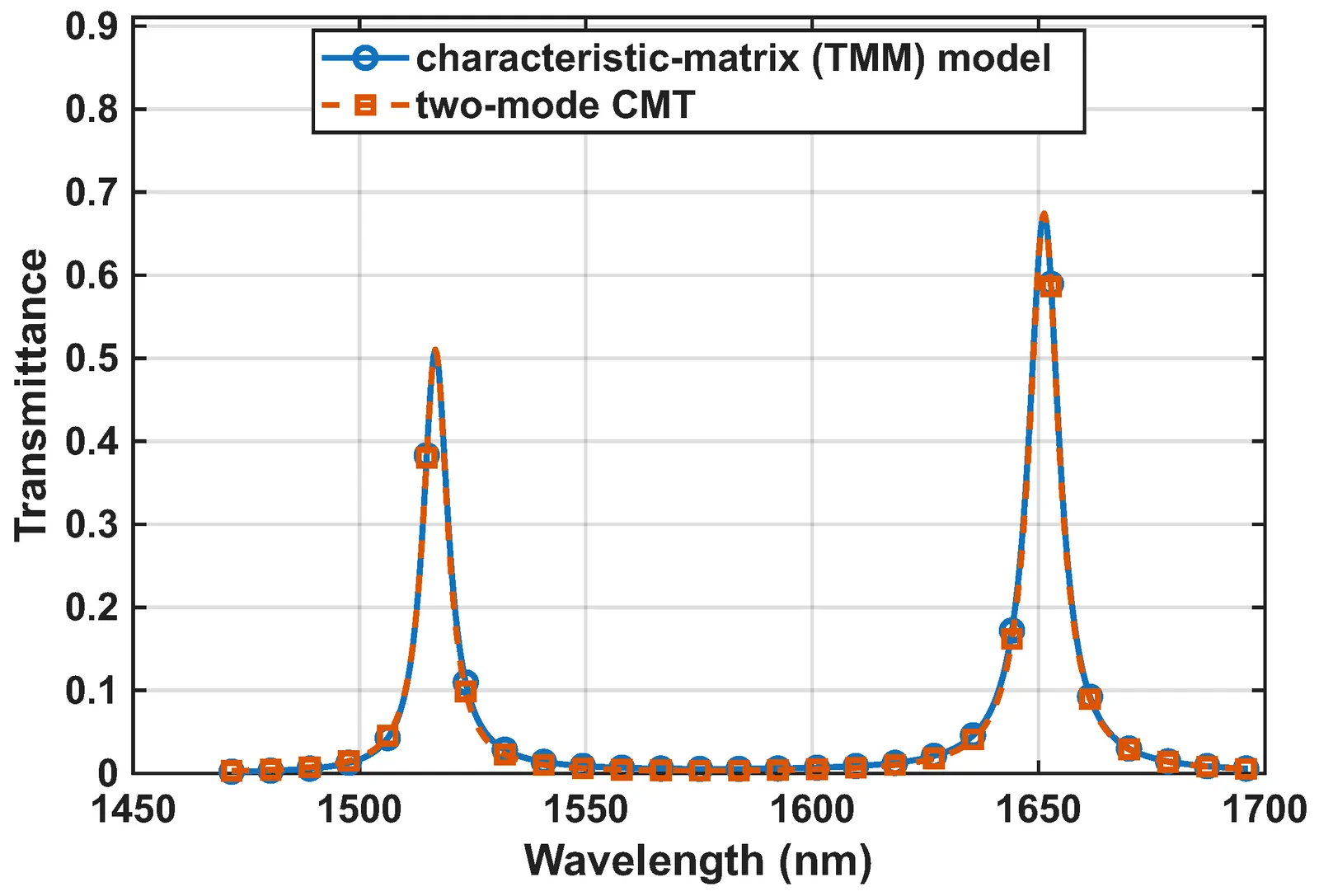
What follows from the Zak-phase difference: the coupled two-mode spectrum. The bulk dispersions and their first-band Zak phases ($\theta_{\mathrm{Zak}}^{U_{1}}=\pi$ and $\theta_{\mathrm{Zak}}^{U_{2}}=0$) are the premise of the argument and are plotted at full width in Figure 4; this figure shows the consequence that a transmission measurement can actually see. The solid curve is the full multilayer spectrum from the production characteristic-matrix (transfer-matrix) forward model and the dashed curve is the two-mode coupled-mode-theory reconstruction built from the two resonance wavelengths, loaded quality factors and peak transmittances alone. The stabilized Redheffer S-matrix implementation reproduces the same spectrum to an RMSE of $2.5\times 10^{-15}$ and is used as an independent numerical-conditioning audit rather than as the production solver (Section S11.2 of the Supplementary Material). The terminated-crystal reflection phase and symmetry-survival audits are shown in Figure 5 and in Figure S12 of the Supplementary Material, and the spatial profiles that identify which mode sits on which side of the interface are reported in Section 3.5.

**Figure 4:**
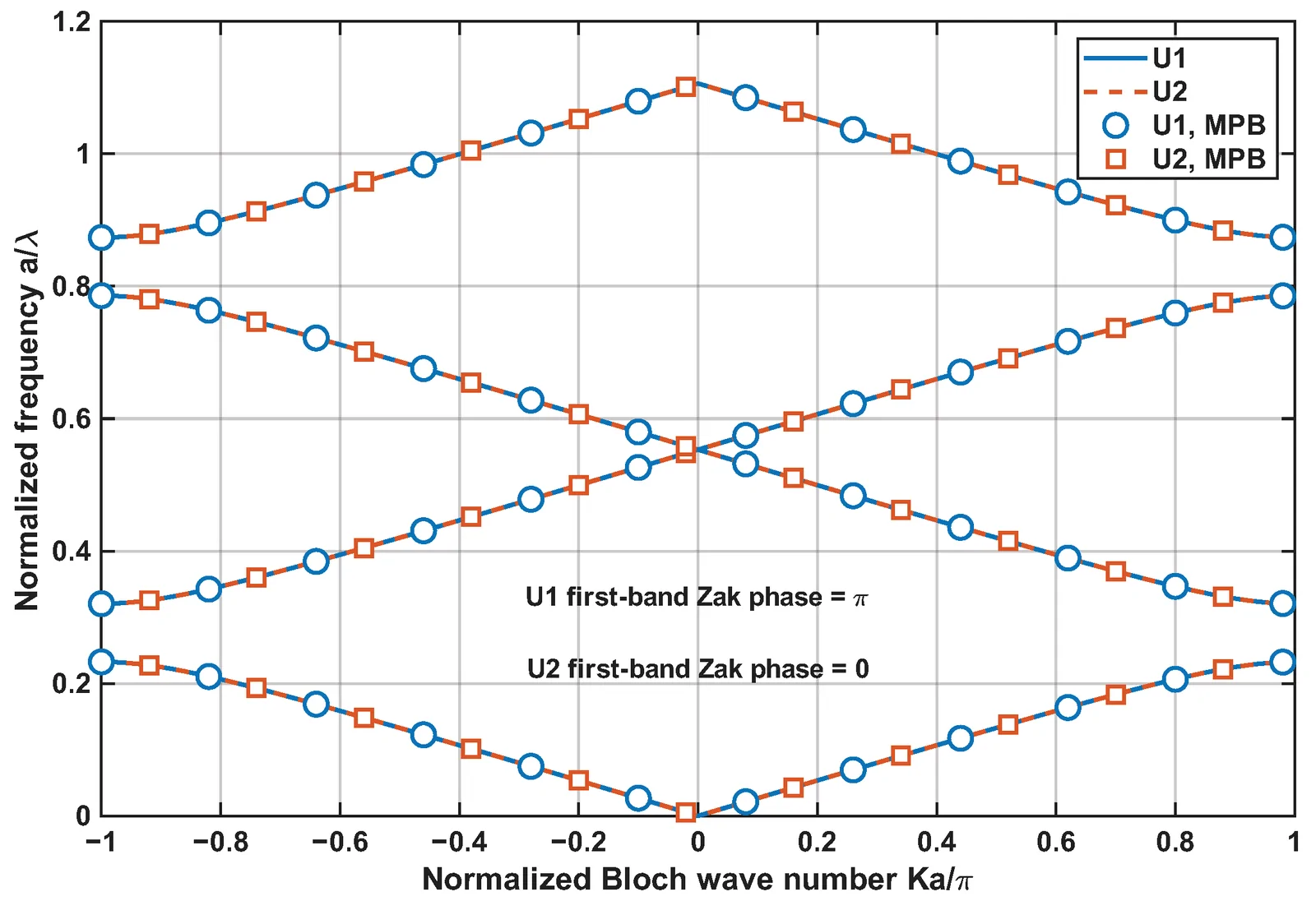
Bulk dispersions and first-band Zak phases of the two translation-related unit cells. Solid curves are $U_{1}$ and dashed curves are $U_{2}$, plotted as normalized frequency a/λ against normalized Bloch wave number Ka/π for the first four bands; the two dispersions overlap because the cells differ only in the position of the inversion center. The annotation inside the first common gap gives the first-band Zak phases obtained from the parity eigenvalues at K=0 and K=π/Λ, $\theta_{\mathrm{Zak}}^{U_{1}}=\pi$ and $\theta_{\mathrm{Zak}}^{U_{2}}=0$. Open circles ($U_{1}$) and open squares ($U_{2}$) are the same bands recomputed with an independent plane-wave eigensolver. They are drawn at a subset of its wave-number grid, offset between the two cells by half a sampling interval, because the two dispersions coincide and markers placed at identical wave numbers would conceal one another. That solver also reproduces the band inversion and the π Zak-phase difference between the two cells; that audit, and the convention that fixes the sign of an individual Zak phase, are reported in Section S11.2 of the Supplementary Material. Band-resolved parity pairs and Zak phases for all four bands are listed in the text of Section 3.1.

Figure 3 shows what that argument produces in the only observable a transmission measurement returns. The solid curve is the full multilayer spectrum from the production cascade; the dashed curve is a two-mode coupled-mode-theory reconstruction given nothing but the two resonance wavelengths, the two loaded quality factors and the two peak transmittances quoted above. That six-number reconstruction follows the cascade across the whole plotted window, including the flanks and the flat region between the resonances, which is the statement that the response really is two coupled modes rather than a pair of unrelated features that happen to fall in the same gap. Figure 4 carries the bulk-band and Zak-phase audit, and its open markers are the same four bands recomputed with an independent plane-wave eigensolver. The two calculations agree to $1.5\times 10^{-5}$ in band-scaled frequency across both cells, and the independent solver reproduces the first-band inversion and the π Zak-phase difference, its parity route matching the production value to double precision and a parity-free Berry-phase route giving 3.1393 rad, a departure from π of 0.07%. The convention that fixes the sign of an individual Zak phase, which the comparison depends on, is set out with the rest of that audit in Section S11.2 of the Supplementary Material.

In Figure 4, the bulk dispersions of $U_{1}$ and $U_{2}$ coincide across the whole Brillouin zone, so band structure alone cannot distinguish the two crystals; what separates them is the first-band Zak phase, π for $U_{1}$ and 0 for $U_{2}$. Inversion symmetry quantizes that phase to 0 or π, so the difference is discrete and, under a fixed inversion-center convention and an inversion-preserving deformation, cannot change without a gap closing. The finite-state assignment therefore rests on the termination-dependent Zak phase, the reflection phase and the localized field profile together, not on the unit-cell labels or on a transmission peak.

**Figure 5:**
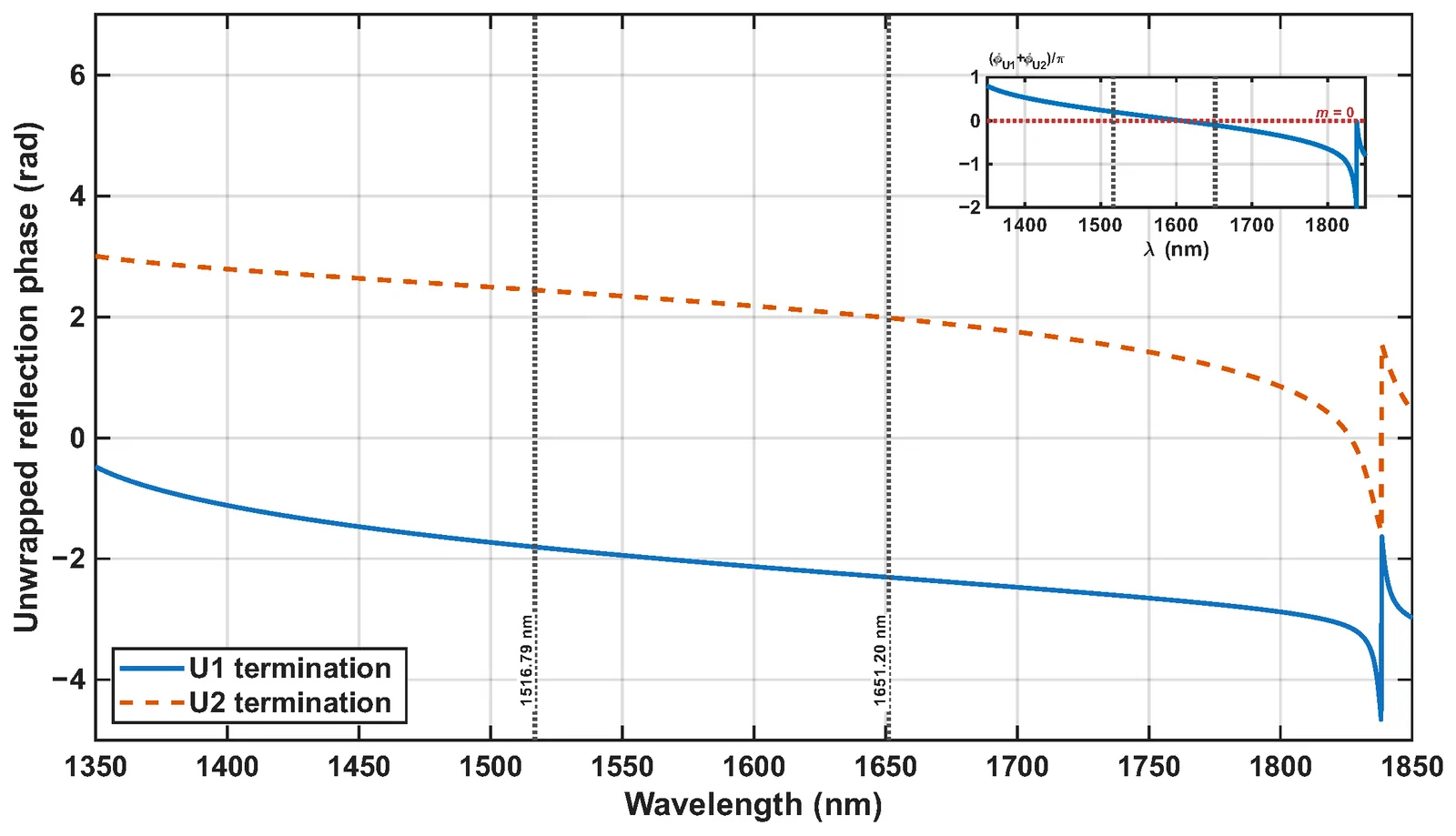
Reflection-phase evidence for the interface condition. The two terminated crystals carry opposite reflection-phase branches within the common stopband: the $U_{1}$ phase stays negative and the $U_{2}$ phase stays positive over most of the interval, and both vary rapidly near the long-wavelength gap edge. The two baseline resonances at 1516.79 and 1651.20 nm are marked by the black dotted vertical lines, so the phase branches can be read at the wavelengths where the modes actually form. The inset gives the ideal-interface phase sum $(\phi_{U_{1}}+\phi_{U_{2}})/\pi$ against its 2mπ reference, that is, the direct-interface condition of Equation (S18) of the Supplementary Material; the red dotted line is the m=0 reference, the only one inside the plotted range, on which the phase sum vanishes, and the black dotted vertical lines mark the same two resonances as in the main axes. The finite device adds the central-section phase of the round-trip condition, Equation (S20) of the Supplementary Material, which displaces the crossings from the ideal-interface values to the marked resonances; the inset is therefore the reference condition, not a prediction of the resonance positions. It is that round-trip condition, satisfied at two wavelengths, which fixes the number of interface resonances at two.

Figure 5 plots the two reflection-phase branches through the common stopband with the baseline resonances marked, so the branches can be read at the wavelengths where the modes actually form; the inset gives the ideal-interface phase sum against its 2mπ reference lines, and the displacement between those crossings and the marked resonances is exactly the central-section phase that the finite device adds.

What the symmetry audit does not establish is that it is equally load-bearing, and Figure S12 of the Supplementary Material bounds it. Three quantities routinely reported together behave quite differently here. Over an inversion-breaking detuning sweep reaching δ=0.85, the bulk parity magnitude falls essentially to zero, while the normalized gap width never departs from unity by more than $1.3\times 10^{-4}$; already at δ=0.30 the parity has lost 16% with the gap intact, so gap persistence certifies almost nothing about the symmetry that produced it. The interface state stays localized through the same detuning with only a modest change in participation ratio, so localization is a weak test too. Modal identity is the quantity that would matter for sensing, and it is not protected at a resolvable level. Across eight disorder amplitudes, the identity correlations of symmetry-preserving and symmetry-breaking ensembles fall almost together, from 0.998 at σ=0.010 to about 0.70 at σ=0.160. Their separation reaches 0.021 at exactly one amplitude (σ=0.096, difference 0.0206, 95% interval 0.0065 to 0.0266) and reverses sign at the two largest. Preserving the inversion symmetry therefore buys no resolvable protection of modal identity over most of the range tested, which is the numerical reason why the topological claim in this paper is made about the specification yield of a matched comparison rather than about immunity to disorder.

### 3.2. Nanolaminate-Induced Modal Engineering

**Figure 6:**
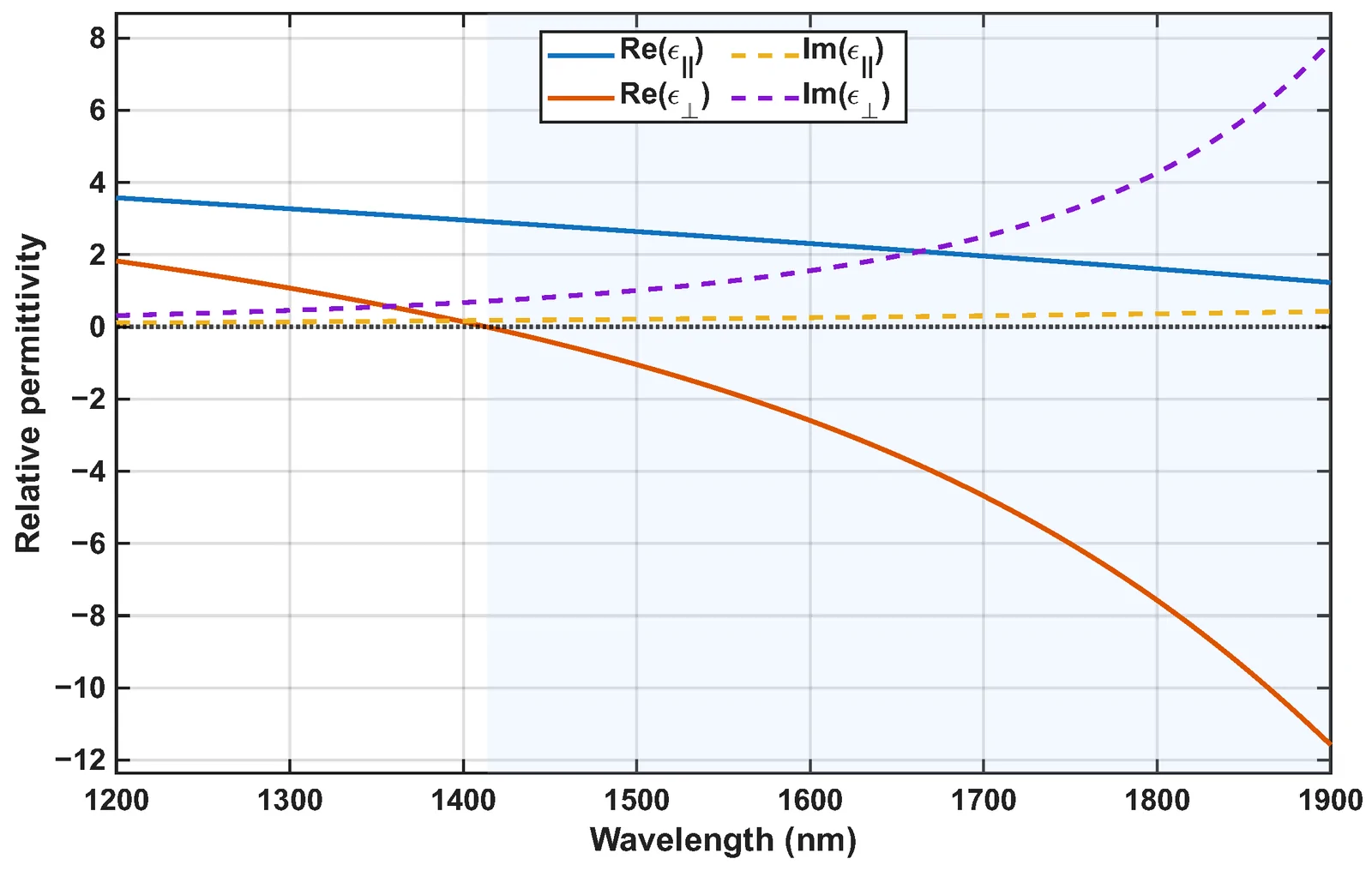
Effective-permittivity tensor of the ITO/TiO_2_ nanolaminate at filling fraction f=0.5 and reference temperature $T_{0}$, computed from the uniaxial effective-medium relations of Section 2.3.2 with the baseline ITO Drude model. Solid curves are Re(ε‖) and Re(ε⊥); dashed curves are the corresponding imaginary parts. Shading marks the hyperbolic interval Re(ε‖)Re(ε⊥)<0, which opens near 1414 nm, where Re(ε⊥) crosses zero and extends past the 1900 nm audit limit, so the upper edge is a support-censored lower bound. Both interface resonances, at 1516.79 and 1651.20 nm, lie inside the window and are Type I. The monotonic rise of Im(ε⊥) across the same interval is the loss penalty that prevents hyperbolicity from being optimized independently of loaded *Q*.

Figure 6 gives the sign structure of the effective permittivity tensor over 1200–1900 nm, with the shading marking the interval in which Re(ε‖)Re(ε⊥)<0. Only Re(ε⊥) changes sign, near 1414 nm, while Re(ε‖) stays positive throughout, so the window is Type I over its whole extent rather than switching character between the two modes, and it is a genuine hyperbolic interval rather than a high-index one. Both operating wavelengths fall inside it: the nearest tabulated tensor values are $\varepsilon_{\|}=2.584+0.218i$, $\varepsilon_{⊥}=-1.279+1.084i$ at the first resonance and 2.133+0.281i, −3.579+1.971i at the second. That is the condition the optimizer enforces, as a hard gate: a candidate losing Re(ε‖)Re(ε⊥)<0 in either mode is rejected irrespective of its sensitivity, detection limit and yield. The figure also shows what the condition costs. Im(ε⊥) grows monotonically with wavelength and is by far the larger loss component, 1.971 against 0.281 for Im(ε‖) at the second resonance, so driving a mode deeper into the window buys anisotropy at the price of layer-normal absorption, Joule loss in the metallic constituent being the inherent limitation of these media [40,41], and in this design hyperbolicity cannot be optimized independently of *Q* and optical access. The upper edge is not reached within the 1900 nm audit limit and is reported as a lower bound.

Replacing the two homogenized slabs by their explicit 5 nm ITO/5 nm TiO_2_ constituents gives a transmittance RMSE of $2.26\times 10^{-3}$ and a maximum absolute deviation of $1.216\times 10^{-2}$, with no resolvable peak exchange or wavelength offset, and the higher-wavelength resonance stays the more transmissive peak in both models, so relative external accessibility is preserved too. That justifies using the effective description for rapid optimization while retaining the explicit bilayer as the physical reference; the division of labour is set out in Section S1.3 of the Supplementary Material, and the agreement is specific to this period and wavelength range and does not remove the known nonlocal and finite-period limitations of layered effective-medium theory [56]. The sensor-level consequence of the loading is a modified distribution of modal energy between the analyte and reference regions; Section 3.4 quantifies it, and the price is linewidth, the loaded *Q* of the two modes falling by 14.6% and 13.0% when the layer is present. The deletion audit of Section 3.3 bounds what this may be credited with: the two response channels survive, indeed improve, when the layer is removed, so neither the nanolaminate nor its hyperbolic classification is responsible for a net sensing-performance advantage.

**Figure 7:**
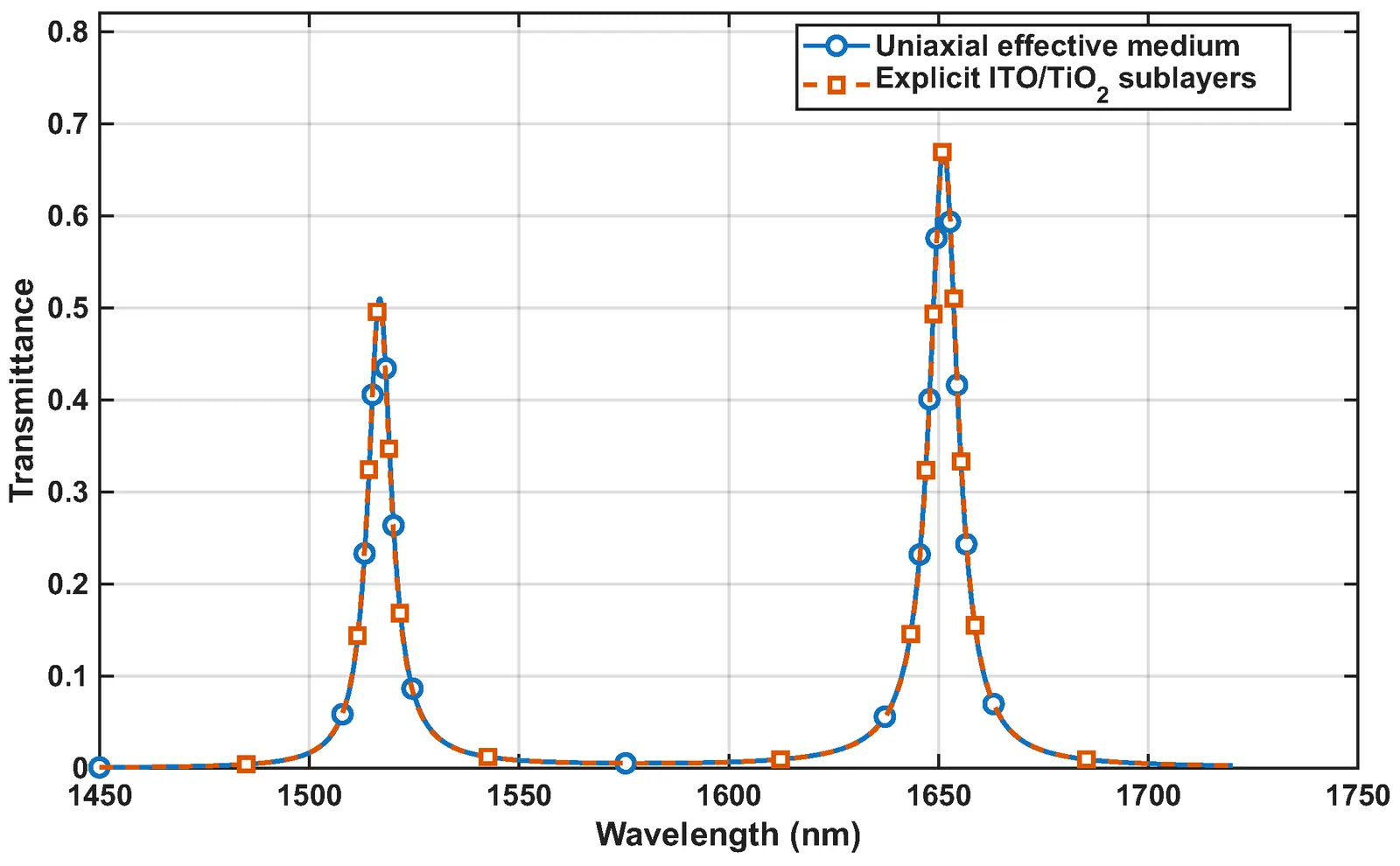
Effective-medium substitution tested against the explicit constituent stack. Transmittance of the complete device over 1450–1720 nm with the two nanolaminate slabs homogenized into uniaxial effective media (solid) and with their explicit 5 nm ITO/5 nm TiO_2_ sublayers retained (dashed); all other layers, material models, and solver settings are identical. Both interface resonances are reproduced without wavelength offset or peak exchange, with a transmittance RMSE of $2.26\times 10^{-3}$ and a maximum absolute deviation of $1.216\times 10^{-2}$. The agreement is specific to the present period and wavelength range and does not remove the known nonlocal and finite-period limitations of layered effective-medium theory.

Figure 7 shows that comparison inside the complete device: both resonances appear at the same wavelengths with the same ordering of peak heights and no exchange of modal identity, and the residual concentrates on the resonance flanks, where the transmittance derivative is largest, rather than at the peak centers.

### 3.3. Structural Audits: Interface, Nanolaminate and Hyperbolicity

Three structural claims are easy to assert and harder to earn: that the modes are interface states rather than tuned cavity resonances, that the nanolaminate earns its place, and that the hyperbolicity condition constrains something the measurement can see. Each is audited here against the same forward model that produces every other number in this paper, and each audit is reported whichever way it comes out.

The first audit closes the cavities. In the working geometry, the two half-crystals never touch: a 600 nm analyte region and a 500 nm reference region separate them, so the bulk–boundary argument is never tested at the working geometry. Scaling the analyte, reference and nanolaminate thicknesses together by a factor α interpolates from the bare contact at α=0 to the working device at α=1. At α=0, with nothing left to tune, the bare ${\left(U_{1}\right)}^{4}\left|{\left(U_{2}\right)}^{2}\right|{\left(U_{1}\right)}^{4}$ stack carries two states inside the common stopband (1340.00 to 1822.50 nm), at 1519.25 and 1719.50 nm; keeping the nanolaminate and closing only the cavities gives 1535.00 and 1736.75 nm. The Zak-phase mismatch therefore does force in-gap states at the junction, and their existence owes nothing to cavity tuning.

The continuation, however, does not connect those states to the operating modes. Tracking every in-gap transmission peak across 101 values of α resolves four branches. The two present at α=0 move to longer wavelengths as the cavities open and leave the gap through its upper edge at α≈0.11 and α≈0.51; the two modes used for sensing enter through the lower gap edge at α≈0.50 and α≈0.64 and reach 1651.25 and 1516.75 nm at α=1. Keeping or closing the nanolaminate changes these crossings by less than 0.02 in α, so the pattern belongs to the cavities. The junction is thus topological in the sense the invariants describe and hosts untuned in-gap states, but the pair used for sensing are cavity-selected resonances of that junction rather than the adiabatic continuation of its bare-contact states. Only the single-parameter path described here was tested; other trajectories through the $\left(d_{n},d_{r}\right)$ plane were not explored.

What the two modes may then legitimately be called follows from that result, and the terminology of this paper is fixed accordingly. The bulk invariants act on the finite device through one quantity only: the sign of the reflection phase of each terminated half-crystal inside the common gap, which the Zak phase fixes through the surface impedance [23,24]. That sign enters the round-trip condition of Equation (S20) of the Supplementary Material directly, so the two operating resonances are eigenmodes of a cavity bounded by two mirrors whose reflection-phase branches are of topological origin, and Figure 5 shows the resonances sitting on those branches. That is a constraint on where in-gap solutions can form and on how the reflection phase moves under an inversion-preserving deformation; it is not adiabatic continuity with the bare-contact states, and it is not protection, which Figure S12 of the Supplementary Material shows to be unresolvable for modal identity. The modes are therefore described here as cavity-selected interface resonances of a topological junction. The trivial controls of Section 3.9 carry the same two cavities without the phase mismatch and also host two modes, which is a second reason not to attribute the existence of the pair to topology; what the matched comparison measures is whether the junction changes how the pair behaves under disorder, and that is the only topological claim retained.

The second audit deletes the nanolaminate. Table 3 reports the complete metric set for the working stack and for the same stack with its two 10 nm layers removed and nothing else changed; both columns come from the same routine, and the loaded quality factors in the first agree with the production values of 0.6% and 1.6%, the residual being peak-refinement tolerance. Every measured quantity improves when the layer is deleted. Loaded *Q* rises by 16.9% and 14.7%, peak transmittance by 47% and 29%, the RI figure of merit by 18% and 20%, the determinant by 16.7%, and the scaled condition number falls from 8.201 to 7.301, while specification yield rises from 0.942 and 0.975 to 0.983 and 1.000 in the systematic-global and combined-realistic scenarios. No metric measured in this work favors the nanolaminate. Two caveats limit how far that travels: the comparison deletes the layer without re-optimizing around its absence, and the resonances shift by about 11 nm, so the two configurations are not compared at identical wavelengths. Both cut against the layer rather than for it, since a stack that was never re-adjusted for its absence still outperforms the selected design that contains it.

That table records what the nanolaminate costs but not what the cost is made of, and the difference decides whether it can be paid down. The loss decomposition of Section 3.4 settles it: removing the nanolaminate absorption entirely would raise the loaded *Q* only to 281 and 239, which is most of the 272 and 242 that the nanolaminate-free stack of Table 3 already reaches. The penalty in that table is consequently a mirror-coupling effect more than an absorption effect, and thinning the nanolaminate would not recover it.

The question that follows is which of the two stacks should be built, and the answer is the one without the layer. The final rows of Table 3 carry the comparison through to the quantities a user would specify. Deleting the nanolaminate leaves the per-device RI limit unchanged, $4.35\times 10^{-5}$ against $4.36\times 10^{-5}$ RIU, because Section 3.6 shows that limit to be set by the index-standard uncertainty and not by the resonance; it improves the temperature limit by 5%, to 0.117 °C, through the larger thermal slopes; and it reduces the transferred temperature limit by 12% and its penalty factor from 203 to 188, while the RI transfer penalty stays at 81 because it is slope scatter of the same relative size in both stacks. The only entry that does not improve is the correlated-local yield, 0.992 against 1.000, one sample in 120 and within the binomial uncertainty of either number. Nothing measured in this work therefore favors the nanolaminate, and the nanolaminate-free stack, two dielectric mirrors around two cavities with no ultrathin conductive oxide to deposit, is the configuration recommended for fabrication; it also removes the least certain material input of the model, the 5 nm ITO constituent. The loaded stack remains the case study reported everywhere else in this paper for a reason that is practical rather than scientific: the calibration surfaces, the matched-control families, the fabrication ensembles, and the five cross-solver validations were all built on it, and rerunning them on the other stack would change every number. Because the detection-limit and calibration-transfer framework being demonstrated does not depend on which stack it is applied to, we do not expect the conclusions to change; that expectation has not been verified, however, since the calibration surfaces, control families and cross-solver validations have not been rebuilt on the nanolaminate-free stack, and Section 4.3 lists this among the boundaries of the evidence. Where a reader needs the recommended stack’s figures, Table 3 now provides them.

The third audit corrupts the layer-normal permittivity. The hyperbolicity condition Re(ε‖)Re(ε⊥)<0 is a hard gate during optimization and the stated reason a measured wafer-top ITO film is rejected, so it should act on something the measurement can resolve. It does not, for a structural reason: the layer transfer matrix uses $q_{z}=\sqrt{\varepsilon_{\|}-\left(\varepsilon_{\|}/\varepsilon_{⊥}\right)s^{2}}$ for TM and $q_{z}=\sqrt{\varepsilon_{\|}-s^{2}}$ for TE with $s=n_{\mathrm{in}}\sin\theta$, so $\varepsilon_{⊥}$ enters only multiplied by $s^{2}$, never enters TE, and drops out identically at θ=0. Replacing $\varepsilon_{⊥}$ by $\varepsilon_{\|}$, or reversing its sign, changes the normal-incidence transmittance by exactly zero to double precision in both polarizations, so the sensitivity matrix, the calibration surface, the detection limits, the yield and every matched control, all evaluated at normal incidence, cannot distinguish the constrained design from an unconstrained one. The constraint is not physically vacuous: at 15° in TM, making the layer isotropic moves the resonances by 0.35 nm and the transmittance by 0.047, and reversing the sign of $\varepsilon_{⊥}$ moves them by 44.8 nm and 0.671. It is defensible only as a TM angular-robustness requirement; it is not a design condition for any headline result, and the rejection of the measured film on the strength of Re(ε⊥) alone is withdrawn accordingly.

Whether the condition earns even that place was then tested directly, since it was the only role left to it. The TM and TE responses were swept from 0 to 30°, twice the declared envelope, for the nominal hyperbolic tensor, for an isotropic-equivalent layer in which $\varepsilon_{⊥}$ is replaced by $\varepsilon_{\|}$ with everything else unchanged, and for the stack without the nanolaminate (Figure S17 and Table S12 of the Supplementary Material). Over the declared 0–15° TM range, the hyperbolic layer reduces the root-mean-square angular drift of the two resonances from 9.68 to 9.57 nm and from 10.81 to 10.64 nm, changes of 1.1% and 1.5%, while lowering the worst-case loaded-*Q* retention from 0.935 to 0.908 and the worst-case transmittance retention from 1.000 to 0.991; the local RI sensitivities of the two treatments differ by less than 0.1 nm/RIU at every angle. At 30° the drift advantage is still 1.1% and the *Q* retention has fallen to 0.618 against 0.759. The nanolaminate-free stack sits between the two on drift and *Q* and above both on transmittance. In TE, the two tensor treatments are identical to machine precision, as Equation (4) requires. There is thus no measurable sensing or robustness benefit attributable to the hyperbolic response at any angle tested: the anisotropy trades about one percent of angular drift for a larger loss of linewidth and throughput, because Im(ε⊥) is the dominant loss component in Figure 6 and oblique TM illumination is precisely what samples it. The hyperbolicity condition is accordingly removed from the design specification altogether, rather than retained as an inactive constraint. It survives only as a material classification of the ITO films in Section 3.10, and the nanolaminate-free recommendation above makes the classification moot for fabrication.

### 3.4. Field Overlap, Loss Budget and the Sensitivity Matrix

Everything this paper reports downstream (the conditioning, the detection limits, the calibration transfer and the yield) is a function of the sensitivity matrix S. The electromagnetic design question is therefore not how to make a resonance sharp or a slope large, but what in the field distribution sets each entry of S, because that is the only quantity a layer stack can be designed against. First-order perturbation theory answers it directly for a one-dimensional resonator. Writing the normalized electric-energy fraction that mode *i* places in functional region *ℓ* as

(25) Γℓ,i=∫ℓReε(z)|Ei(z)|2dz∫Reε(z)|Ei(z)|2dz,

a perturbation, $\delta n_{a}$, confined to the analyte region shifts the resonance by $\delta \lambda_{i}/\lambda_{i}=\Gamma_{a,i}\;\delta n_{a}/n_{a}$ [51], so the two columns of S read

(26) $$S_{n,i}=\frac{\lambda_{i}\;\Gamma_{a,i}}{n_{a}},\;S_{T,i}=\lambda_{i}\sum_{\ell }\Gamma_{\ell ,i}\;\theta_{\ell },\;\theta_{\ell }=\frac{1}{n_{\ell }}\frac{\partial n_{\ell }}{\partial T}+\alpha_{\ell }.$$

The two channels are structurally different, and that difference is the whole of the design problem. The index channel reads one region and is therefore bounded by a single overlap fraction. The thermal channel reads every region, weighted by the same overlaps but against each material’s thermal optical-path coefficient $\theta_{\ell }$, which combines its thermo-optic response with its expansion. A layer that contributes nothing to $S_{n}$ can dominate $S_{T}$.

Equation (26) is a linearization of a lossy, dispersive, strongly coupled structure, so it was checked rather than assumed. The computed overlaps are $\Gamma_{a}=0.0924$ and 0.2700 for modes 1 and 2, $\Gamma_{\mathrm{ref}}=0.2311$ and 0.1240, and $\Gamma_{\mathrm{HMM}}=0.0097$ and 0.0048. Substituted into Equation (26), these predict 105.39 and 335.19 nm/RIU, against local numerical derivatives of 100.00 and 328.00 nm/RIU: agreement to 5.4% and 2.2% with no fitted parameter. The same relation predicts the figure of merit as $Q\Gamma_{a}/n_{a}$, giving 16.10 and 42.21 ${\mathrm{RIU}}^{-1}$ against numerical values of 15.28 and 41.30. The residual is larger for mode 1, which is the more weakly analyte-coupled of the two and therefore the one for which the neglected radiative and dispersive corrections are a larger share of a smaller quantity. What matters for design is that both entries of the index column are predicted, to within a few percent, by one geometric number per mode.

The thermal column is not dominated by the analyte at all, and Table 4 shows why. Summed over a 100 K excursion, the optical path of the 12 high-index and 18 low-index mirror layers grows by 16.03 and 2.88 nm, the two nanolaminate slabs by 0.85 nm and the analyte channel by 0.40 nm, all positive; the single 500 nm reference coating moves by −15.68 nm, the only negative entry in the stack. Its own index change supplies −22.50 nm of that and its expansion returns +7.05 nm, so the coating fights itself and still wins. The net for the whole stack is +4.48 nm, a 22% residual of two competing contributions that are each three to five times larger. A temperature channel built this way is intrinsically a difference of large numbers, which is the physical reason the thermo-optic coefficient of the reference material is a materials requirement rather than a tolerance and the reason both thermal slopes are negative despite every other layer pushing the other way.

Combining the two columns turns Equation (26) into an explicit design rule, and the steps are worth setting out because the result turns out not to be a statement about sensitivity at all. Substituting both entries of Equation (26) into $\det S=S_{n,1}S_{T,2}-S_{n,2}S_{T,1}$ and collecting the common factor gives

(27) $$\det S=\frac{\lambda_{1}\lambda_{2}}{n_{a}}\;\Gamma_{a,1}\Gamma_{a,2}\sum_{\ell }\left(v_{\ell ,2}-v_{\ell ,1}\right)\theta_{\ell },\;v_{\ell ,i}\equiv \frac{\Gamma_{\ell ,i}}{\Gamma_{a,i}},$$

in which every absolute overlap has canceled. What survives is the difference between the two modes’ ratios of non-analyte to analyte overlap, weighted by the material coefficients; the analyte’s own term vanishes identically because $v_{a,i}=1$ for both modes. Separating the reference coating, whose coefficient the design specifies and which carries the only strongly negative, $\theta_{\ell }$, in the stack, from the layers whose coefficients the mirror materials fix,

(28) $$\det S=S_{n,1}S_{n,2}\;n_{a}\left(\theta_{\mathrm{REF}}\;\Delta x+\Delta y\right),\;\Delta x=v_{\mathrm{REF},2}-v_{\mathrm{REF},1},\;\Delta y=\sum_{\ell \neq \mathrm{REF}}\left(v_{\ell ,2}-v_{\ell ,1}\right)\theta_{\ell }.$$

Three consequences follow at once. The matrix is singular on the straight line $\Delta y=-\theta_{\mathrm{REF}}\Delta x$, whose slope is set by the reference material alone and carries no geometric information, so a stack can move within this plane but cannot move the line. A design’s margin against inseparability is consequently its distance from that line rather than the magnitude of either sensitivity. And because Δx and Δy are differences of ratios, two modes with identical overlap ratios give a singular matrix, however sensitive each one is individually, while two modes with equal sensitivities but different ratios stay separable. Section S12 of the Supplementary Material carries the intermediate algebra and states the approximation each step inherits. Here, the reference-to-analyte overlap ratio $\Gamma_{\mathrm{ref}}/\Gamma_{a}$ is 2.50 for mode 1 and 0.459 for mode 2, a factor of 5.45. That factor, and not the 3.6-fold difference in RI sensitivity, is the quantity the geometry has to deliver. The design objective for a dual-parameter interface-mode stack is consequently to break the overlap ratio between the two coupled resonances, which is what the asymmetric placement of the analyte and reference cavities about the $U_{1}|U_{2}$ junction does. The same statement read along the material axis is the reference-coating requirement of Section 3.10: holding the geometry fixed and sweeping $\partial n_{\mathrm{ref}}/\partial T$ moves the two ratios together until they cross, and detS passes through zero at $-2.67\times 10^{-4}$ $K^{-1}$.

**Figure 8:**
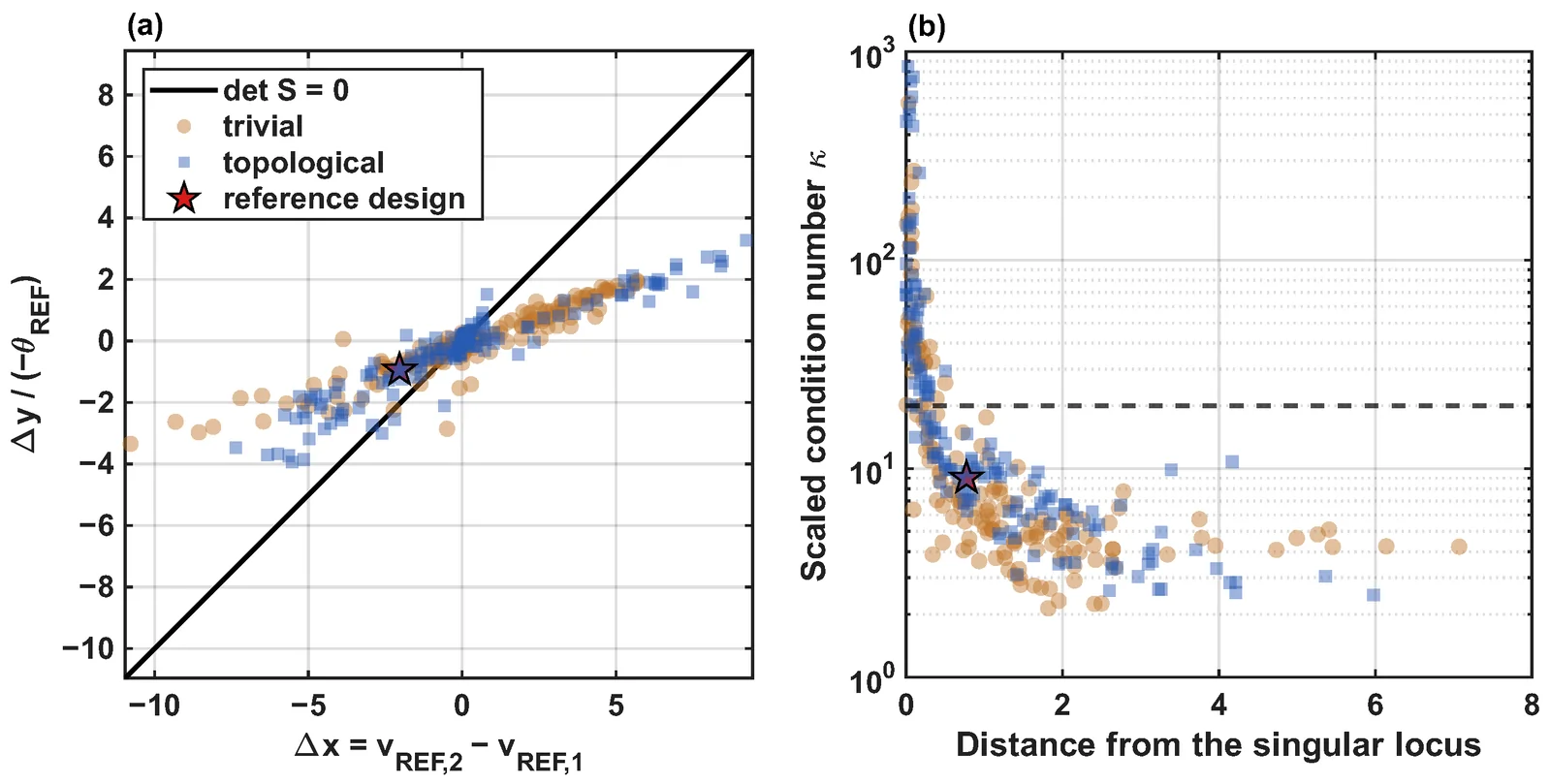
The sensitivity matrix as a design map, tested against the forward model. Each point is one geometry drawn from the declared design box by Latin hypercube sampling, 160 per architecture plus the reference design, evaluated with the full transfer-matrix model; 317 of 322 samples resolved two interface modes and are shown. (**a**) Normalized overlap differences on axes chosen so that the perturbation identity $\det S\propto \theta_{\mathrm{REF}}\Delta x+\Delta y$ puts the singular locus on the diagonal (solid line). The identity assigns the correct side of that line to 92.7% of the samples. Axis limits cover the central 98% of the cloud; 7 samples from the corners of the box fall outside and are not drawn. (**b**) Scaled condition number against distance from the locus, with the declared κ≤20 ceiling marked by the dashed line. The two are related by a rank correlation of −0.879, so margin from the locus is what governs invertibility. The identity predicts the position of the locus and the ordering of the margins; it does not predict the value of detS, whose median relative error is 24.4% away from the locus, because the thermal column it is built from is a difference of contributions three times larger than their sum.

Equations (26) and (28) describe one operating point, so they were tested as design rules rather than quoted as such; on the axes of Figure 8a the singular locus of Equation (28) is a straight line fixed by the materials alone. Both architectures were sampled over the declared design box, every sample evaluated with the full forward model rather than with the relation being tested, and 317 of 322 resolved two modes. The relation places a design on the correct side of the locus in 294 of those 317 cases, 92.7%, and the distance from the locus orders the conditioning with a rank correlation of −0.879 overall, −0.930 within the topological family and −0.812 within the trivial one. What it does not do is predict the value of the determinant: the median relative error is 24.4% for designs in the outer half of the distance distribution and 32.7% for the inner half.

That accuracy limit is a property of the quantity rather than of the linearization: the determinant is built from a thermal column that is itself a difference of large opposing contributions, −6.07 nm against +10.77 nm leaving +4.48 nm in Table 4, so first-order estimates carrying a few percent of error individually carry tens of percent onto their difference, and on the locus the relative error diverges by construction. The map is therefore usable for the two things it was tested on, which side of the locus a geometry falls on and how much margin it has, and Figure 8b shows that the margin, not the magnitude of either sensitivity, sets the conditioning: the median κ falls from 52.7 for geometries within 0.25 of the locus to 9.94, 5.64 and 4.13 in the successive distance bands, the worst case in the outermost band being 10.8 against 848 in the innermost. The reference design sits at a distance of 0.772, where the map’s own recomputation returns κ=9.03 against the production value of 9.14; Section 3.8 shows that the search objective was a compressed proxy for this margin. The map also has a boundary: 64.8% of topological and 76.6% of trivial samples fall within the declared κ≤20 ceiling, so conditioning alone does not distinguish the two architectures, and the separation reported in Section 3.9 rests on fabrication yield and mode identity rather than on the determinant.

The remaining electromagnetic budget is the loss, which does not enter S at all but sets how precisely a wavelength can be read from it, since resonance-center repeatability scales with the linewidth at fixed signal-to-noise. Recomputing the spectrum with every extinction coefficient set to zero splits $1/Q=1/Q_{\mathrm{rad}}+1/Q_{\mathrm{abs}}$: $Q_{\mathrm{rad}}=281.2$ and 239.1 against loaded values of 231.7 and 207.9, so $Q_{\mathrm{abs}}=1315$ and 1595 and dissipation is only 17.6% and 13.0% of the total loss rate. The absorbed fractions A=1−T−R are 0.154 and 0.293, the latter nine times mode 2s reflected fraction of 0.032, so the second resonance is well coupled and lossy while the first is under-coupled, reflecting 0.336. The design consequence is specific: removing the nanolaminate absorption entirely would raise the loaded *Q* only to 281 and 239, improving the linewidths by 21% and 15%, while the mirror stack sets the remaining 82% and 87% of the loss. Thinning or purifying the ITO is therefore not the lever on the detection limit; adding mirror periods is, and that lever trades directly against the peak transmittances of 0.510 and 0.674, hence against the signal-to-noise the narrower line was bought for. This tension places the device three orders of magnitude below a $Q>10^{5}$ platform on $S_{n}/\mathrm{FWHM}$, and it is a property of a transmission-read multilayer rather than of the topological mechanism.

### 3.5. Dual-Parameter Sensing Performance

One convention governs every number in this and the following sections and is stated first: the temperature channel reports the temperature of the device, not of the analyte. The analyte is modeled without a thermo-optic coefficient, so the reference coating is the only element whose index responds to temperature, and the retrieved $\hat{T}$ is the multilayer temperature that a stage or a package would set. The case of an aqueous analyte whose own index moves with temperature is treated below; the retrieved temperature then remains the device temperature, and the retrieved index becomes the analyte index at that temperature. The two modes responded differently to RI and temperature. At the nominal operating point, the numerical sensitivity matrix was

(29) $$S=\left[\begin{array}{cc} 90.7055 & -0.047799\\ 329.4134 & -0.015600 \end{array}\right],$$

where the first column is in nm/RIU and the second in nm/°C. Mode 2 was approximately 3.63 times more RI-sensitive than mode 1. Both modes shift negatively with temperature, and mode 1 does so about three times faster than mode 2. No sign difference is available to appeal to, and none is needed, so the mechanism is stated precisely. The determinant factorizes identically as

(30) $$\det S=S_{n,1}S_{n,2}\left(\frac{S_{T,2}}{S_{n,2}}-\frac{S_{T,1}}{S_{n,1}}\right),$$

so the matrix is invertible precisely when the two modes have different thermal-to-index response ratios. Those ratios are $-5.27\times 10^{-4}$ and $-4.74\times 10^{-5}$ RIU/°C, a separation of $4.80\times 10^{-4}$ dominated by mode 1. Term by term, detS=−1.415+15.746=14.331, so the only term containing $S_{T,2}$ supplies −9.9% of the total; reversing the sign of $S_{T,2}$ at fixed magnitude would move the determinant by 19.7%, to 17.161, and would not threaten identifiability in either direction. Whether the two thermal coefficients share a sign is therefore a visible consequence of where the ratios sit, not the mechanism. The distinction is not stylistic: an earlier version of this model gave the analyte layer the thermal expansion of the liquid rather than of the channel wall, and correcting it (Section S6 of the Supplementary Material) moved $S_{T,2}$ from +0.00452 to −0.01560. A separability argument written on signs would have been reversed by that correction; the ratio argument survives it, because the determinant moved by 0.95%.

That conclusion can be tested by moving the two ratios apart and together. The model above gives the analyte no thermo-optic coefficient, which sits awkwardly with the motivation for dual-parameter operation since temperature has to be measured alongside index precisely because it moves the analyte index. Water, the fluid the $1.33\leq n_{a}\leq 1.40$ window implies, has $\partial n_{a}/\partial T≃-1\times 10^{-4}$ $K^{-1}$ near 25 °C and $≃-2.5\times 10^{-4}$ $K^{-1}$ near 80 °C. Including it replaces $S_{T,i}$ by $S_{T,i}+c\;S_{n,i}$, which adds a multiple of the index column to the temperature column and leaves the determinant algebraically unchanged. That is a statement about the linearization, so it was checked against the full forward model, where the resonances are relocated at every temperature and no cancellation is guaranteed: the determinant moves from 14.332 at c=0 to 14.238 and 14.301 at the two coefficients above, at most 0.65%, and the scaled condition number from 8.20 to 8.48 and 9.11, all far below the declared threshold of 20. The thermal slopes themselves move a great deal over the same range, from (−0.0478,−0.0156) to (−0.0711,−0.1005) nm/°C, and at the strongest coefficient, mode 2 becomes the faster of the two, reversing which mode dominates the temperature channel. That the determinant survives a reordering of that size is the substance of the result, and it is a robustness finding rather than a caveat.

What does change is the meaning of the temperature channel, and the two cases should not be conflated. With c=0, the reference layer is the only thermally responsive element, and the device reports its own temperature; with the analyte term included, the index channel returns the analyte index at the temperature the device simultaneously reports, which is what a chemical measurement wants. Everything that follows is computed with c=0 and is therefore a device-temperature result, and the sweep above bounds the move to an aqueous sample at under 12% in conditioning and 1% in determinant. The RI figures of merit $S_{n}/\mathrm{FWHM}$ are 13.86 and 41.48 ${\mathrm{RIU}}^{-1}$. One convention needs stating because two different slopes appear in this paper. The entries of S are least-squares fits over the whole calibrated interval, since that is the interval the reported limits and yields refer to. The response is not exactly linear over so wide a range, so forward differences at $\Delta n_{a}=0.01$ give 99.0–100.1 nm/RIU for mode 1 and 329.0–330.2 for mode 2, the spread depending on whether the effective or the explicit nanolaminate is perturbed: mode 2 agrees with the fitted value to 0.25%, while the mode 1 local slope runs 9–10% above it. The perturbation studies of Section 3.10 and Section 3.11 quote local slopes because that is what a single perturbation changes, while the detection limits and retrieval use the fitted matrix. The gap between the two estimators measures the curvature that motivates the bounded nonlinear inversion; it is not a disagreement between calculations.

The matrix determinant was 14.3307 in the corresponding mixed units. After scaling, the parameter axes by 0.01 RIU and 10 °C, the two-norm condition number was 8.2006. This value indicates usable, but not ideal, separation: local inversion is stable enough for initialization and covariance propagation, while a nonlinear bounded calibration remains preferable over the complete two-dimensional range. The response-vector engineering achieved here is conceptually consistent with dual-resonance RI–temperature sensors based on coupled cavities, nanobeams, quasi-BIC slabs, and valley-Hall modes [9,13,14,32].

**Figure 9:**
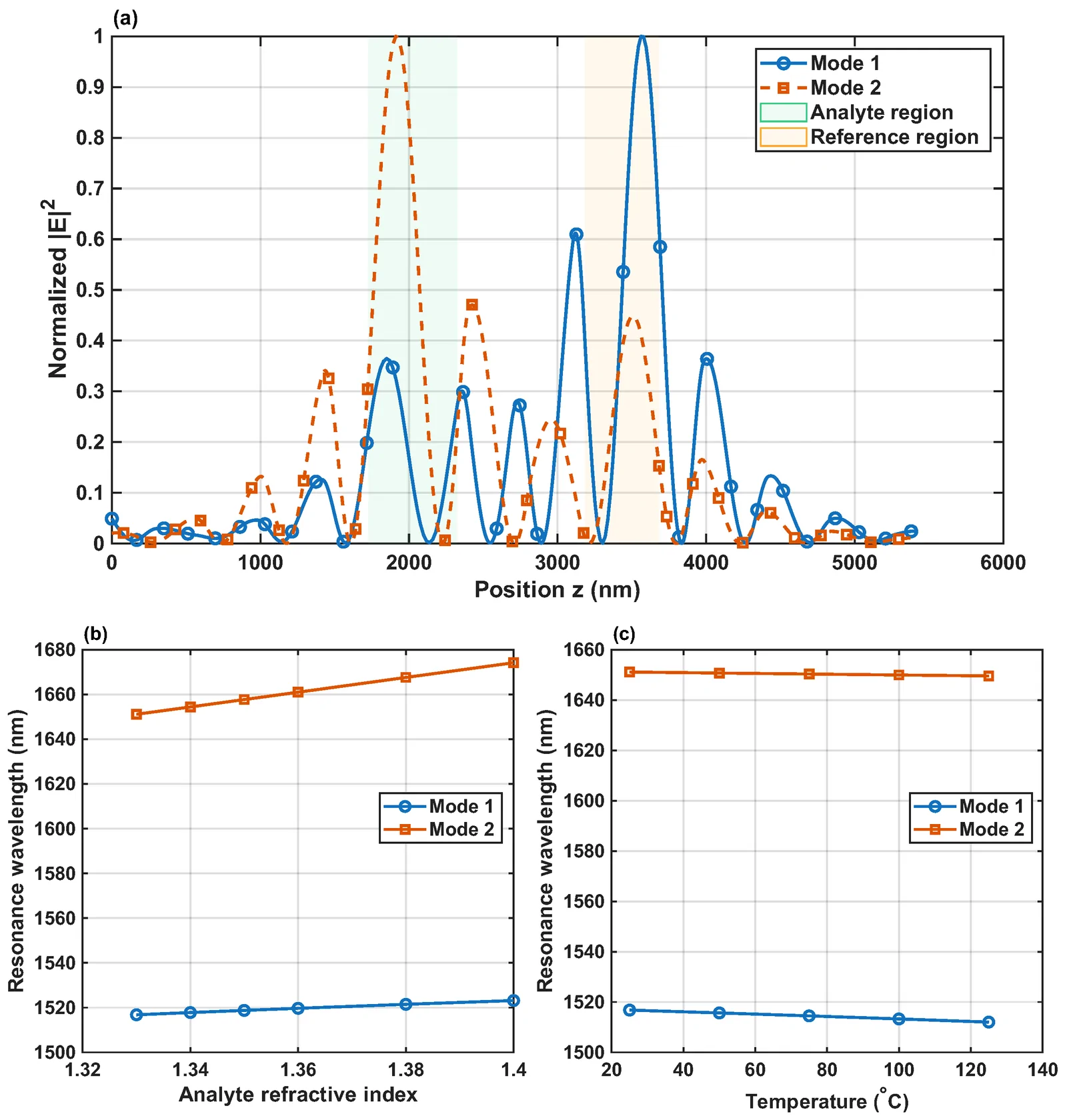
Where the two modes sit, and what each one responds to. (**a**) Spatial localization of the coupled interface resonances of the topological junction: the normalized ${\left|E\right|}^{2}$ profile of mode 1 concentrates on the reference side of the interface and that of mode 2 on the analyte side, which is the physical origin of their unequal response vectors. The green and orange shaded bands mark the analyte region and the reference region, respectively. (**b**) Fitted refractive-index slopes over the full calibrated interval, 90.7 and 329.4 nm/RIU for modes 1 and 2. (**c**) Fitted thermal slopes, −0.0478 and −0.0156 nm/°C. Both thermal slopes are negative, and both index slopes are positive, so no sign difference separates the two channels; what keeps the response directions from collapsing onto one another is the separation of the modes’ thermal-to-index ratios, $-5.27\times 10^{-4}$ against $-4.74\times 10^{-5}$ RIU/°C.

**Figure 10:**
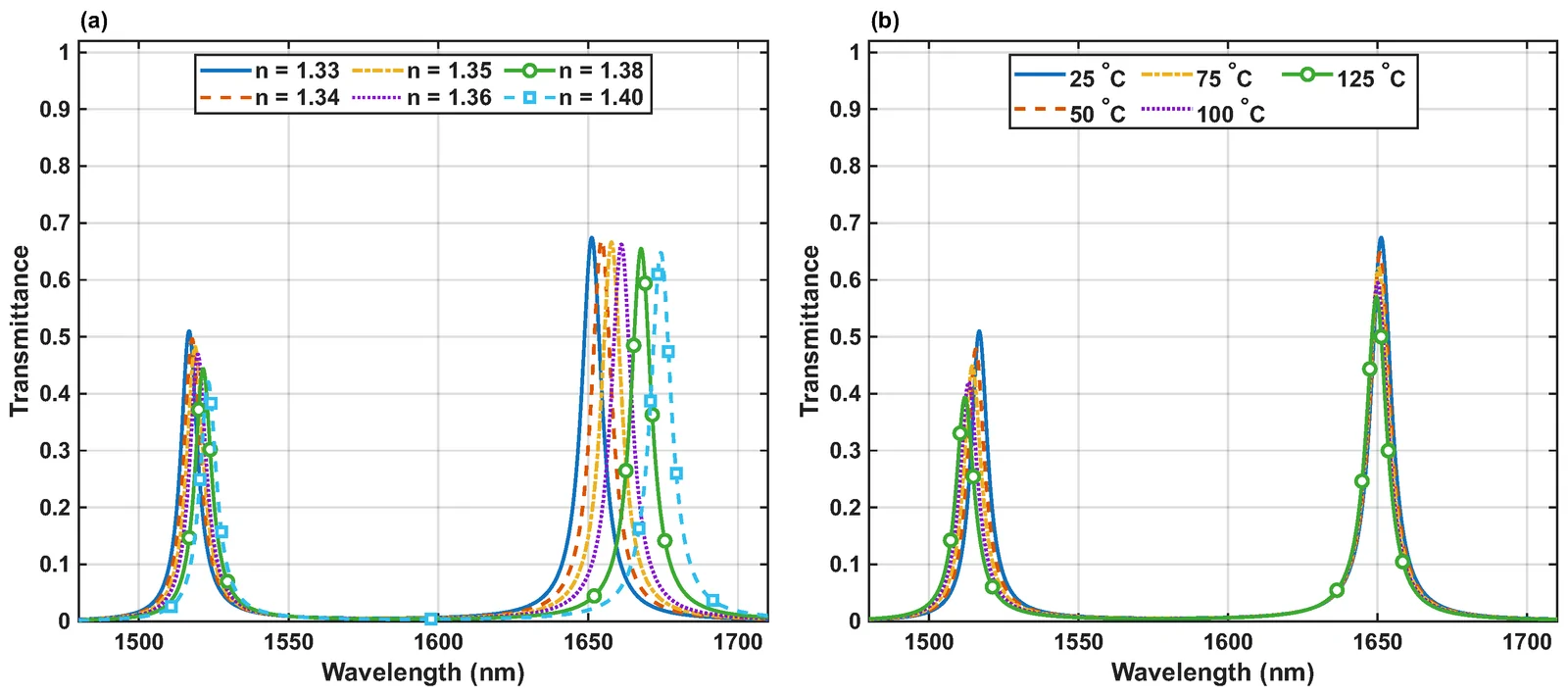
Raw dual-resonance spectral response to both measurands, computed with the production characteristic-matrix forward model [57] on a common wavelength grid. (**a**) Transmittance over the analyte refractive-index sweep $n_{a}=1.33$–1.40 at the reference temperature. (**b**) Transmittance over the temperature sweep 25–125 °C at the nominal analyte index. Under an index change, both resonances redshift by very different amounts; under a temperature change both blueshift, at rates differing threefold. The two response vectors stay non-parallel because the modes’ thermal-to-index ratios differ, as Equation (30) of the article makes explicit; the direction of the thermal shifts plays no part in that argument.

Over the sampled operating coordinates, the two modes trace clearly unequal response vectors: as $n_{a}$ increases from 1.33 to 1.40, they shift by 6.35 and 23.06 nm, while over 25–125 °C, mode 1 blueshifts by 4.78 nm against 1.56 nm for mode 2. Figure 9a shows the spatial origin of that asymmetry. The normalized ${\left|E\right|}^{2}$ profile of mode 1 peaks inside the reference band at z=3562 nm and that of mode 2 inside the analyte band at z=1911 nm; neither is confined to its own region, but the imbalance is significant, with mode 1 having fallen to 0.325 at the analyte peak and mode 2 to 0.411 at the reference peak. These are two differently weighted modes of one structure rather than two peaks of a single delocalized resonance, which is what allows one perturbation to move them by different amounts. The two sweeps behind the fitted slopes are continuous rather than switching between peaks: both resonances move monotonically to longer wavelengths and stay resolved throughout the index range, and in the temperature sweep, both peaks move monotonically to shorter wavelengths, with the lower-wavelength one being about three times as fast. The accompanying transmittance and line-shape changes are what the prominence, linewidth and identity gates in the nonlinear calibration exist to catch. Figure 10 carries both sweeps in full.

Figure 10 shows the two sweeps themselves, the raw observable on which everything downstream is built. Under an index change, both resonances redshift by very different amounts and stay more than 130 nm apart, so no peak-tracking ambiguity arises; under a temperature change, both blueshift, at rates differing threefold. The accompanying changes in peak transmittance and linewidth are what the prominence, linewidth and identity gates of the nonlinear calibration exist to catch.

### 3.6. Nonlinear Retrieval and Detection Limits

The nonlinear calibration contained 72 exact forward solutions: eight RI values and nine temperatures. On the interleaved validation grid, the bounded inversion achieved an RI RMSE of $2.433\times 10^{-5}$ RIU and a temperature RMSE of 0.1545 °C under the readout-noise model; the same grid solved without readout noise gives $2.430\times 10^{-5}$ RIU and 0.1536 °C, with maximum absolute errors of $4.585\times 10^{-5}$ RIU and 0.3723 °C. All validation inversions converged within the calibrated domain. Two features of the held-out parity, plotted in Figure S10 of the Supplementary Material, matter more than the summary statistics. The scatter about the identity line is uniform along the whole range rather than growing toward the ends, which is the signature that would appear if a single local sensitivity matrix were being extrapolated beyond its linear neighborhood. The maximum absolute error, $4.585\times 10^{-5}$ RIU, is 1.9 times the corresponding noise-free RMSE for the index, and 0.3723 °C is 2.4 times it for temperature, so the worst held-out point is a few times the typical one rather than an outlier of a different order. The temperature channel is the looser of the two, consistent with the temperature column of S being the weaker one.

**Figure 11:**
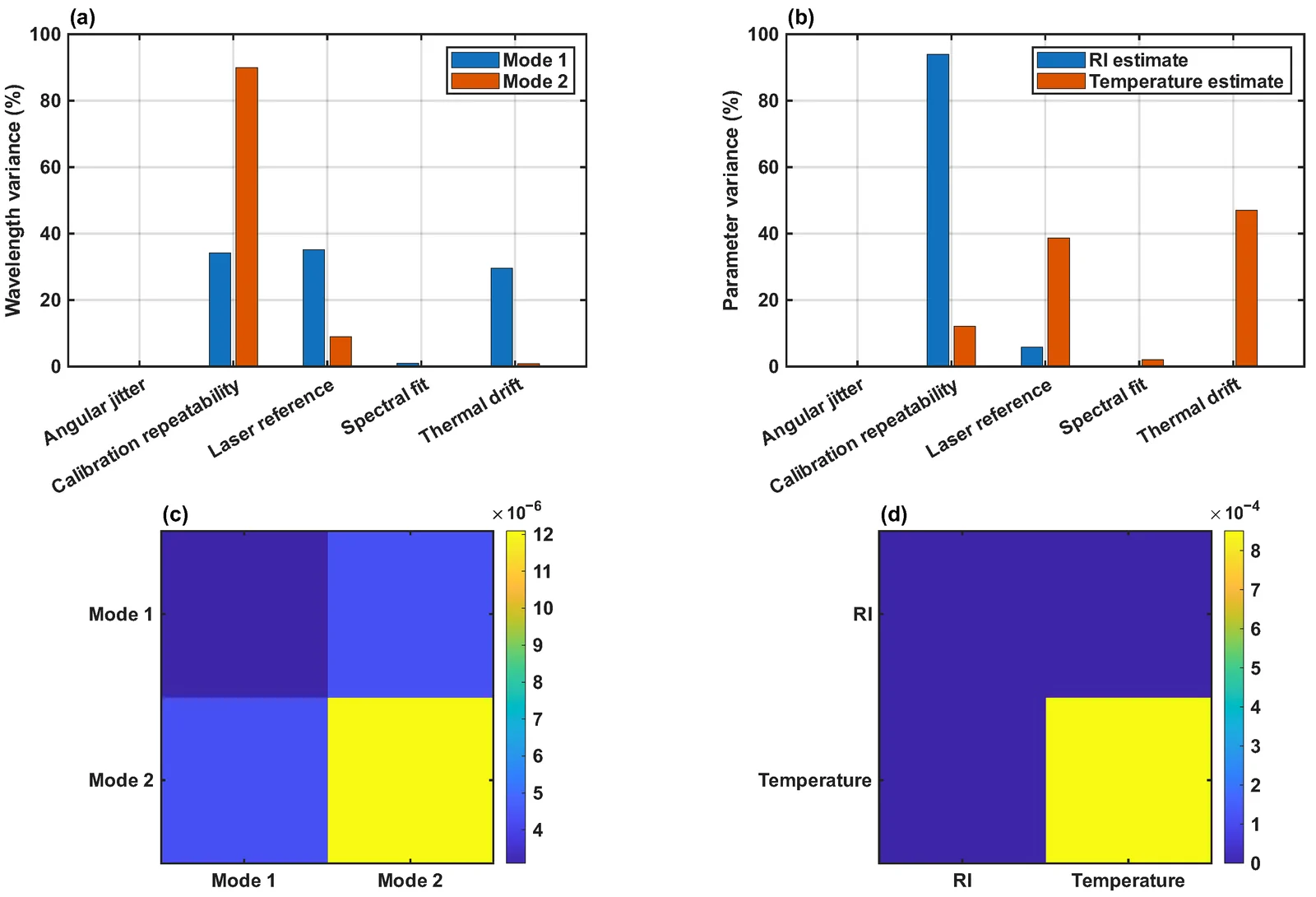
Full measurement-error budget behind the detection limits. (**a**) Per-source contributions in resonance-wavelength space. (**b**) The same sources after propagation through the sensitivity matrix into recovered-parameter space; calibration repeatability dominates the index variance, whereas the temperature variance splits mainly between laser-reference and thermal-drift terms. (**c**) Per-device wavelength covariance $C_{\lambda }$ in nm^2^, whose off-diagonal term corresponds to $\rho_{\lambda_{1},\lambda_{2}}=0.7130$. (**d**) Recovered-parameter covariance $C_{x}$, with correlation −0.1055. The off-diagonal terms are not negligible in either matrix, so independent scalar error bars would misstate both the size and the orientation of the uncertainty.

Under device-specific calibration, the fitted wavelength standard deviations were $1.759\times 10^{-3}$ and $3.477\times 10^{-3}$ nm with correlation $\rho_{\lambda_{1},\lambda_{2}}=0.7130$, and covariance propagation gave $\sigma_{n}=1.03\times 10^{-5}$ RIU and $\sigma_{T}=0.02917\;{}^{\circ}$C with a recovered-parameter correlation of −0.1055. The source-by-source decomposition (Figure 11) shows that the two wavelength variances are not built the same way: mode 1 is shared between the laser reference (35.2%), calibration repeatability (34.2%) and thermal drift (29.5%), whereas mode 2 is almost entirely repeatability (90.0%) with the laser reference at 9.0%. Propagated into parameter space, the asymmetry survives with the roles exchanged: 93.9% of the index variance comes from repeatability and 5.8% from the laser reference, while the temperature variance splits between thermal drift (47.0%), the laser reference (38.6%) and repeatability (12.2%). Reducing one instrumentation term therefore improves one measurand and barely touches the other, and the off-diagonal terms cannot be dropped: the inverse sensitivity matrix turns the strong positive wavelength correlation into a weak negative parameter correlation, so independent scalar error bars would misstate both the size and the orientation of the uncertainty.

The conventional 3σ limits were $3.10\times 10^{-5}$ RIU and 0.0875 °C. Residual biases of $-1.10\times 10^{-5}$ RIU and 0.0507 °C increased the bias-aware minimum detectable changes to $4.20\times 10^{-5}$ RIU and 0.1382 °C. Using a 1% false-alarm target and a 95% detection target, the covariance-based POD limits were

(31) $${\mathrm{LOD}}_{\mathrm{POD},n}=4.36\times 10^{-5}\;\mathrm{RIU},\;{\mathrm{LOD}}_{\mathrm{POD},T}=0.123\;{}^{\circ}C.$$

**Figure 12:**
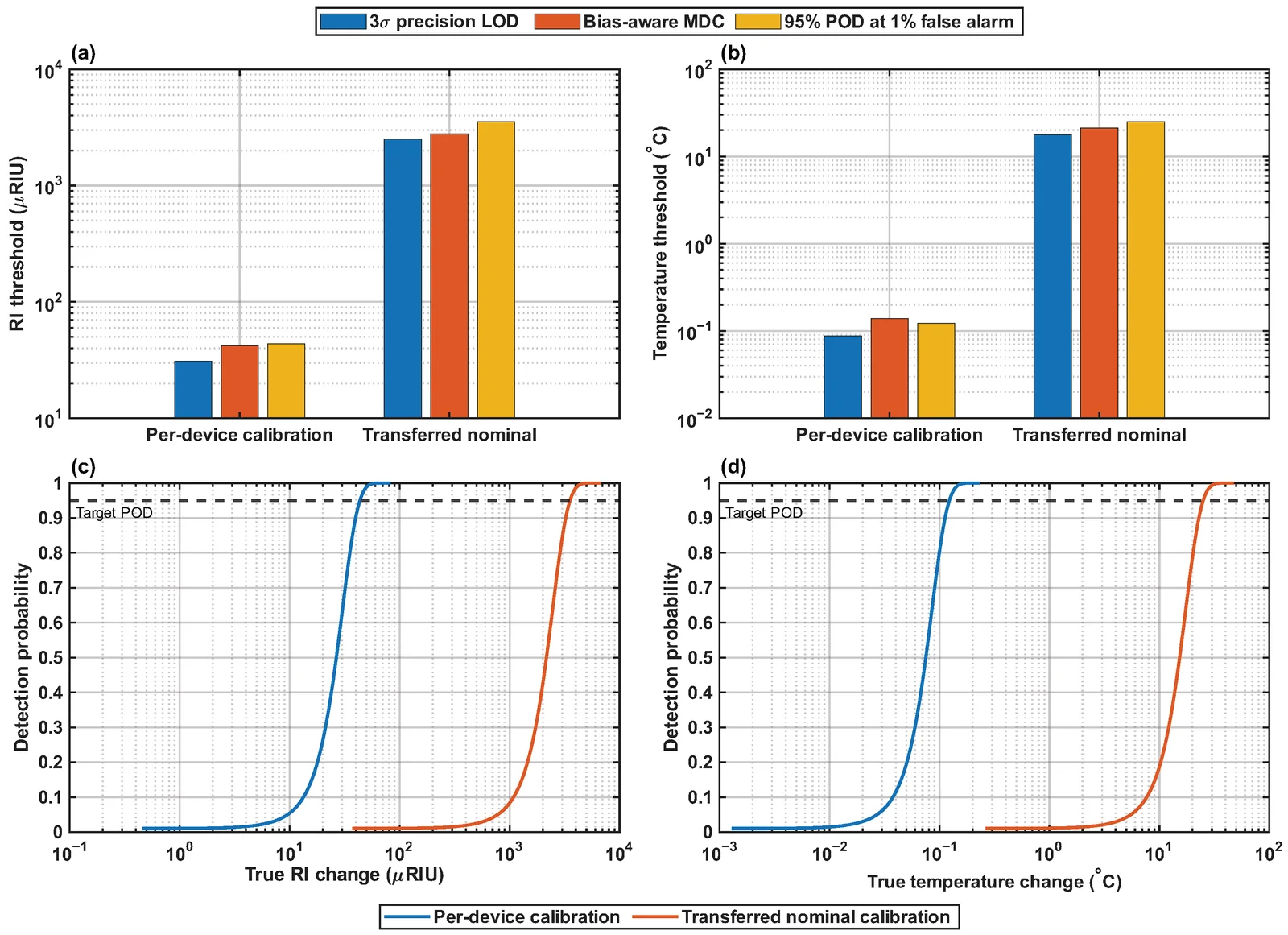
Bias-aware and POD-based detection limits. The upper panels (**a**,**b**) compare 3σ, bias-aware MDC, and POD thresholds for per-device and transferred calibration. Under per-device calibration the three thresholds are $3.10\times 10^{-5}$, $4.20\times 10^{-5}$ and $4.36\times 10^{-5}$ RIU for the refractive index and 0.0875, 0.138 and 0.123 °C for temperature. The lower panels (**c**,**d**) show the detection curves. The per-device curves reach 95% POD at a small RI and temperature changes, whereas transferred calibration shifts the curves by orders of magnitude.

The empirical false-alarm frequencies were 1.03% for RI and 0.83% for temperature, and the detection probabilities at the reported limits were 95.01% and 94.84%, so the decision and detection limits keep their classical distinction [15,16]. Figure 12 places the limits in their calibration context. The per-device 3σ, bias-aware MDC and 95%-POD bars stay close because the residual bias is comparable to the random uncertainty, whereas the transferred-calibration bars move by orders of magnitude: transferring the calibration multiplies the RI POD limit by 81, from $4.36\times 10^{-5}$ to $3.54\times 10^{-3}$ RIU, and the temperature limit by 203, from 0.123 to 25.01 °C. Temperature is the more fragile channel under transfer because its column of S is the weaker one, so the same wavelength offset becomes a larger parameter error. The headline limits therefore require device-specific zeroing and two-dimensional calibration and are not factory-calibration values.

**Figure 13:**
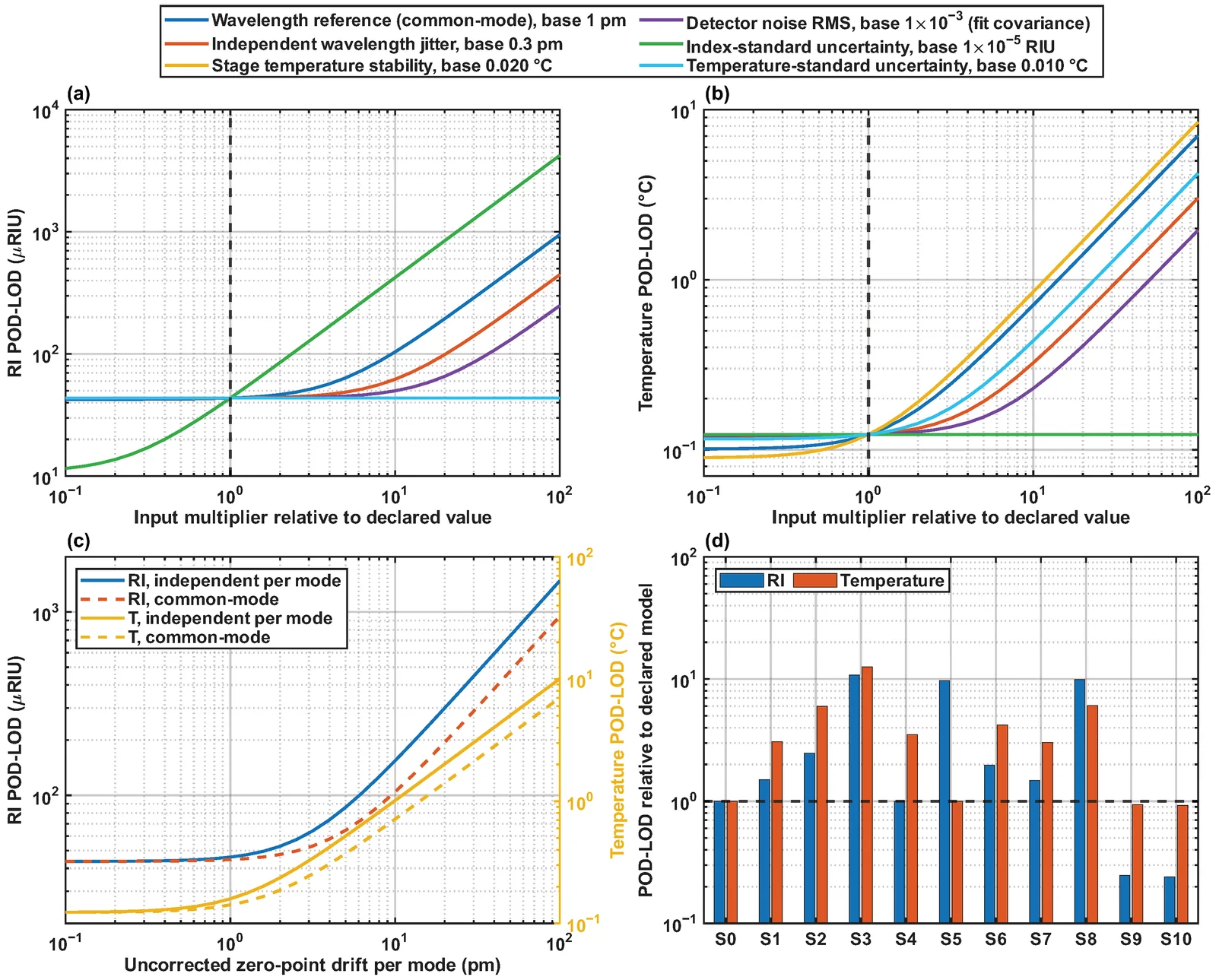
Sensitivity of the reported detection limits to the declared instrument inputs and to drift, computed with the production covariance budget and probability-of-detection chain. (**a**,**b**) RI and temperature POD limits when one input at a time is scaled from 0.1 to 100 times its declared value (vertical dashed line, at a multiplier of one); the RI limit tracks the index-standard uncertainty and the temperature limit the stage stability and wavelength reference, while the detector noise, which sets the spectral-fit precision, barely moves either. (**c**) Both limits are against an uncorrected zero-point drift between calibration and measurement, independent per mode or common to both; a common-mode drift is partly rejected by the inversion. (**d**) Instrument-class and drift scenarios relative to the declared model, S0, whose ratio of one is marked by the horizontal dashed line; the scenarios are defined in Table S10 of the Supplementary Material. S3 is a compact-spectrometer readout, S8 combines a 5 pm reference, 0.1 °C stage, $10^{-4}$ RIU standard and 5 pm drift, and S9 and S10 remove the calibration-standard and fit terms to expose the reference and thermal floor.

Because every entry of the budget behind Equation (31) is an assumed instrument specification rather than a measurement, the limits were recomputed with each input varied over two decades about its declared value, and under a set of instrument-class and drift scenarios (Figure 13; Table S10 of the Supplementary Material lists the scenarios). The result shows what the limits are made of. The RI limit is 93.9% index-standard uncertainty: the $10^{-5}$ RIU assigned to the calibration liquids propagates through $S_{n}$ into both resonances, and $4.36\times 10^{-5}$ RIU is very nearly the POD multiplier of 4.22 applied to that number. It is insensitive to the spectral fit, which supplies 0.3% of its variance, and to the stage temperature and the temperature standard; it grows by a factor of 1.5 when the wavelength reference is relaxed from 1 to 5 pm and by 2.5 at the 10 pm accuracy typical of an optical spectrum analyzer; and it follows the index-standard uncertainty one for one once that exceeds about $2\times 10^{-5}$ RIU. Removing the standard terms altogether would lower it to $1.08\times 10^{-5}$ RIU, the floor set by the wavelength reference and the fit, so the published figure is a statement about the calibration liquids at least as much as about the sensor. The temperature limit is built differently, from 47.0% stage stability, 38.6% wavelength reference and 11.8% temperature standard, so it degrades by a factor of 3.5 when the stage is held to 0.1 °C instead of 0.02 °C and by 3.1 at a 5 pm reference. Neither limit depends on the resonance linewidth within the range tested: the fit term supplies under 3% of either variance, tripling the detector noise moves the RI limit by 1% and the temperature limit by 11%, and the instrument resolution is immaterial between 5 and 200 pm. Drift is the input the budget did not contain and the one a field instrument cannot avoid. An uncorrected zero-point drift of 5 pm per resonance between calibration and measurement doubles the RI limit and multiplies the temperature limit by 4.2, and the drifts that on their own equal one detection limit are 4.0 and 14.3 pm on the two modes for the RI channel and 5.9 and 1.9 pm for the temperature channel. A drift-tolerant readout therefore needs re-zeroing at that level or a wavelength reference shared by both modes, which lowers the penalty, to 1.5 and 3.0 for the same 5 pm, because the inverse sensitivity matrix rejects part of a shift common to both resonances. A compact-spectrometer scenario combining 20 pm wavelength accuracy, $3\times 10^{-3}$ noise, 0.1 °C stage stability and a $10^{-4}$ RIU standard gives $4.7\times 10^{-4}$ RIU and 1.55 °C, factors of 10.8 and 12.6 above the declared model. The published limits are thus tunable-laser figures obtained with certified standards, and are reported as such.

### 3.7. Calibration Transfer and Reduced Per-Device Calibration

**Figure 14:**
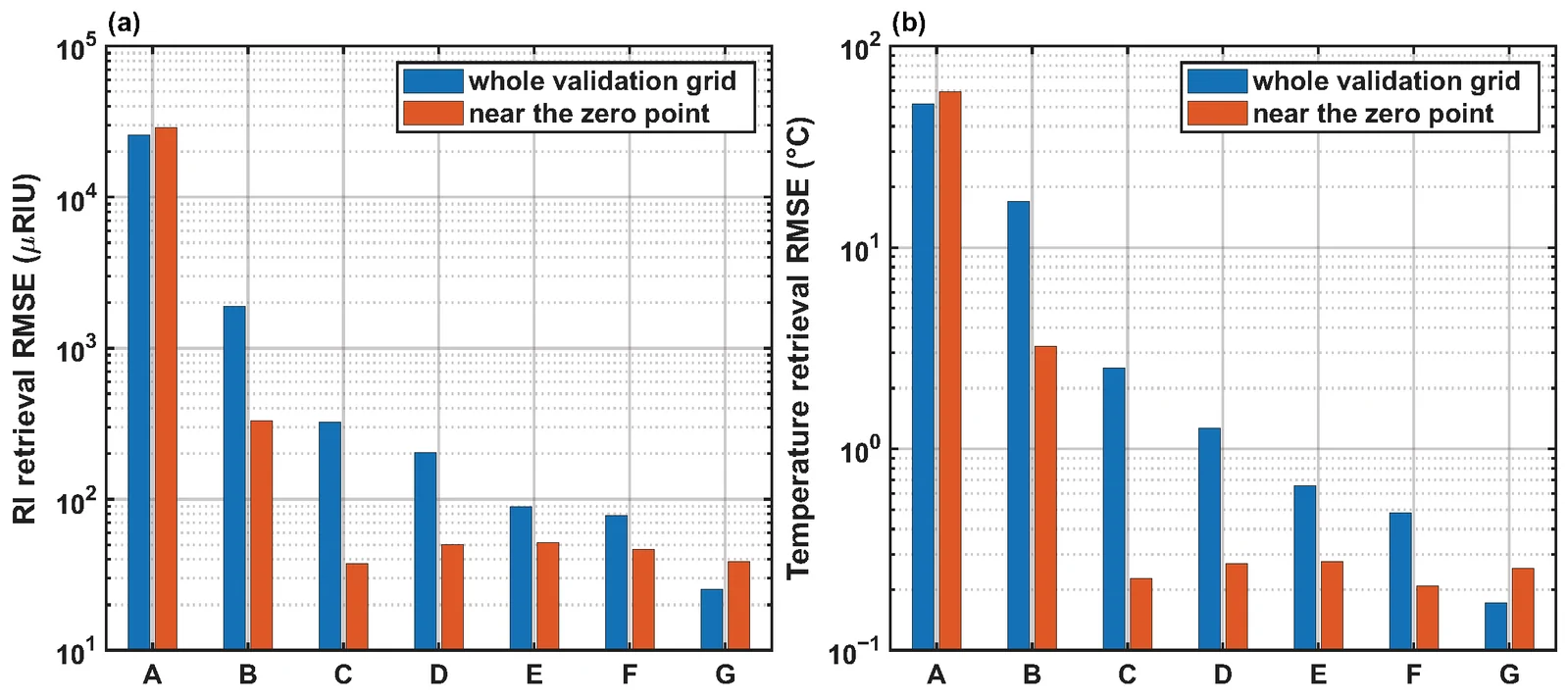
Retrieval error of 30 perturbed devices under seven calibration strategies, evaluated on the 56-point validation grid without measurement noise. A: nominal calibration transferred with no device measurement. B: per-device zero point only. C and D: zero plus an affine slope correction from one index step and one temperature step, with spans of 0.01 RIU and 12.5 °C (C) or 0.04 RIU and 50 °C (D). E: four points and a bilinear correction. F: nine points and a quadratic correction. G: the full 72-point per-device surface. The blue bars are the RMSE over the whole validation grid, and the orange bars are the RMSE at the validation points within one grid interval of the zero. (**a**) Refractive index. (**b**) Temperature. Table S11 of the Supplementary Material lists the values.

The 81-fold and 203-fold transfer penalties describe a device that receives the nominal calibration and no measurement of its own. What a fabricated population needs is the smallest per-device measurement that brings the limits back, and that was quantified rather than argued. Thirty perturbed devices were drawn from the combined-realistic fabrication model, the exact two-mode response of each was computed on the 8×9 calibration grid and on the 56-point interleaved validation grid, and both parameters were retrieved from the validation wavelengths under seven calibration strategies of increasing cost (Figure 14; Table S11 of the Supplementary Material shows every entry). Noise was excluded, so the errors are model-mismatch errors only; the measurement uncertainty of the reference points is the $C_{\mathrm{cal}}$ term already in the budget. The zero points scatter by 11.1 nm on both resonances across the population, so the untouched nominal calibration fails outright, with a retrieval error of $2.6\times 10^{-2}$ RIU and 52 °C and 86% of the retrievals driven to the domain boundary. Re-zeroing each device at one certified point brings the error to $1.9\times 10^{-3}$ RIU and 16.9 °C over the whole calibrated domain, which is the transferred scenario of Section 3.6 evaluated over a wider excursion, and to $3.3\times 10^{-4}$ RIU and 3.2 °C within the first grid interval of the zero; a re-zeroed device is therefore usable only near its zero. Adding two reference measurements, one index step and one temperature step, and correcting the nominal surface by the affine map they determine removes 84% and 86% of the remaining excess error with a span of 0.01 RIU and 12.5 °C, and 90% and 93% with a span of 0.04 RIU and 50 °C, leaving $2.0\times 10^{-4}$ RIU and 1.27 °C over the whole domain and $5.0\times 10^{-5}$ RIU and 0.27 °C near zero, with the latter being indistinguishable from the full 72-point surface at $3.9\times 10^{-5}$ RIU and 0.25 °C. A fourth point adds the bilinear cross term and reaches $9.0\times 10^{-5}$ RIU and 0.66 °C; nine points and a quadratic correction reach $7.8\times 10^{-5}$ RIU and 0.48 °C, three times the full-surface figures of $2.5\times 10^{-5}$ RIU and 0.17 °C. The remaining gap is the device-specific curvature of the two surfaces, which no low-order correction of a nominal surface can carry and which matters only far from the zero.

The practical protocol for a fabricated batch therefore has three tiers, and Figure 2 places them in the chain. Every device needs its own zero, measured at one certified index and temperature; nothing recovers a device whose resonances have moved by ten nanometres. A device that will be read within about 0.01 RIU and 12 °C of that zero, which covers a narrow-range assay, needs two further reference measurements and then reaches the per-device limits; a device that must cover the full domain needs four to nine points and reaches within a factor of three of them. The full two-dimensional surface is reserved for reference instruments. This is the standardization route used for calibration transfer in chemometrics, where a few transfer samples correct a master calibration instead of rebuilding it [52], and it inherits that route’s condition: the correction is only as good as the reference standards, which is exactly the term that dominates the RI limit in Figure 13. Wafer-level metrology can reduce the per-device burden but not remove it. The slope scatter that the correction absorbs is 8.7% and 3.1% on the two RI slopes and 28% and 36% on the temperature slopes across the population, and Table S15 of the Supplementary Material identifies its drivers by standardized regression on the 480-sample fabrication ensemble. The RI slopes are driven by the analyte-cavity thickness and by the indices of the high-index layers and of the reference coating, which ellipsometry can measure; the temperature slopes are driven almost entirely by the thermo-optic coefficient of the reference coating, standardized coefficients −0.80 and −0.67, which is a material-batch property and is measured most cheaply on the device itself, by the temperature step of the three-point correction. The zero-point offsets themselves follow the global thickness error, as Section 3.10 reports.

### 3.8. Design-Space Audit and Monte Carlo Yield

One thing has to be settled before the numbers in this section are read: the selected reference design is not what the search produced but what the search failed to displace. The hybrid retained 433 unique designs from 11,487 exact evaluations, 49 of them feasible and 42 in the approximate Pareto set, and across that feasible cloud the primary objective moves by only 8.0%, from 43.8 to 47.3 μRIU of RI POD-LOD, and by 3.0% across the ten candidates recomputed with the full model. The declared rule keeps the frozen reference unless a challenger improves POD-LOD by at least 2% without degrading fabrication yield by more than 0.02, loaded *Q* by more than 10%, or mode identity. Four of the nine challengers reach a smaller POD-LOD, by at most 0.36%, an order of magnitude below the threshold, and the two closest give up 32 and 28 points of internal 120-sample yield to do it; the rule’s designated best alternative is 0.12% worse on POD-LOD and is nominated only on the secondary criteria. The accurate description is therefore a feasibility-audited, hand-selected reference that survived an exact Pareto audit, not a design the optimizer chose.

The flatness is the more useful half of that finding. The primary objective varies by 3.0% over the exact-recomputed set, while the quantities those designs are also judged on vary far more: internal fabrication yield from 0.517 to 1.000 and the scaled condition number from 5.51 to 18.24. A search driven by RI POD-LOD therefore searches along the one direction in which this design space is nearly degenerate, which is why 11,487 evaluations left the starting point standing, and because challengers are gated on the primary objective, the rule cannot promote a design that is better on the criteria that do discriminate: the conditioning-focused candidate reaches an internal yield of 1.000 and a condition number of 5.51 against the reference’s 0.958 and 9.14 at a cost of 1.5% on POD-LOD, and the pattern-search local optimum reaches 1.000 and 6.41 at a cost of 0.1%. Those internal yields rest on 120 samples each, and their intervals overlap, so this is not a claim that the reference is the wrong design. The surrogate [58] was used only for acquisition, the MADS pattern search [59] supplied the local refinement, the reference-vector evolutionary calculation [60] served only as an independent diversity comparison, and every nondominated representative was recomputed with the full model; the stage sequence and settings are in Algorithm S1 and Table S3 of the Supplementary Material, and the complete Pareto projection is Figure S13 there.

How much discrimination the objective retains can be measured. Across the 36 scored designs of the paretosearch archive the RI POD-LOD correlates with the scaled condition number at +0.82, so it points the right way, but it compresses a 3.5-fold spread in conditioning into an 8.0% spread in itself. Over the ten exact-recomputed candidates, the correlation falls to +0.25 against conditioning, indistinguishable from zero at n=10, and to −0.33 against fabrication yield, while conditioning and yield remain nearly one axis at −0.98. The 2% promotion threshold is 25% of the objective’s range across the archive but 66% of its range across the candidates the rule judged, so at the moment of decision the rule asked for an improvement two-thirds the size of the whole achievable variation in a quantity that no longer tracked what was being traded. The objective’s own winner shows the symptom: the lowest-POD-LOD design ranks eighth on conditioning and ninth on fabrication yield, and promoting it would have surrendered 0.317 of yield for 0.36% of detection limit.

The remedy is a different objective rather than a larger search. Distance from the singular locus of Section 3.4 orders the conditioning at a rank correlation of −0.879 across 317 sampled geometries and does not saturate as the detection limit does; a search steered by that margin, with the detection limit demoted to a constraint, would discriminate where this one could not. Redirecting the search would change the reference design and every result reported here, so the point is recorded as a design conclusion rather than acted on.

The screening fabrication scenarios produced self-referenced yields of 1.000 for local uncorrelated errors, 1.000 for correlated local errors, 0.9417 for systematic global errors, and 0.9750 for the combined realistic scenario, each with 120 samples; a separate post-optimization gate with 300 samples gave 0.9733. These intermediate estimates were not used as the headline yield. The independent confirmation used a new seed and N=1000, of which 959 devices passed every declared spectral, conditioning, retrieval, and manufacturability gate:

(32) $$\hat{p}=\frac{959}{1000}=0.959.$$

The 95% Wilson interval was 0.9449–0.9696 [61], quantifying binomial sampling uncertainty under the stated distributions rather than industrial reliability. Because most realizations retain two identifiable resonances even when some fail linewidth, conditioning or retrieval gates, modal survival alone overestimates usable-device yield, and that argument applies to the declared gates themselves. They are loose relative to what the device achieves: the RI error gate is $1.0\times 10^{-3}$ RIU against a nominal $2.43\times 10^{-5}$, a margin of 41; the temperature gate 5.0 °C against 0.155 °C; and no gate constrains the detection limit at all. A realization could in principle pass everything while being an order of magnitude worse than the headline limit, so the yield and the detection limit must not be read as mutually supporting. Whether that happens can be checked exactly, since the Monte Carlo stores the sensitivity matrix and both quality factors of every realization: propagating the wavelength covariance through each realization’s own S, with its wavelength precision rescaled by its own *Q* under the linewidth-limited argument of Section 4.2, gives a per-device detection limit. Gating on that limit, staying within a factor of two of the nominal, lowers the yield from 0.979 to 0.944 over all four scenarios and from 0.975 to 0.892 in the combined-realistic one. The looseness is real but does not inflate the headline number much, because the realized limits cluster tightly: median $4.49\times 10^{-5}$ RIU, 95th percentile $5.33\times 10^{-5}$, 99th $6.14\times 10^{-5}$, against a nominal $4.36\times 10^{-5}$. The honest labels are that 0.979 is a modal and spectral survival yield and 0.944 a performance-linked yield, the latter being the one to quote beside a detection limit; Figure S15 of the Supplementary Material gives the per-scenario histograms. Neither replaces the headline: 0.944 is a diagnostic computed over the pooled 120-sample screening scenarios under the additional twofold-LOD gate, whereas the specification yield of Equation (32) is the independent N=1000 confirmation under the declared gates, and the two answer different questions.

The yield also depends on an assumed correlation length for layer-thickness errors, a modeling choice rather than a measured property of a deposition tool, so its influence was quantified directly. Recomputing over ρ∈{0,0.25,0.50,0.72,0.90,0.99} with the design, the gates, the sample count (N=300 per point) and the seed all fixed moves the yield from 0.970 for independent errors to 0.973 at the declared ρ=0.72 and 0.980 in the nearly fully correlated limit, a spread of 0.013 narrower than any individual Wilson interval in the sweep. Fully correlated thickness error is close to a common scale factor that the cavity tuning partly absorbs, while independent error is the mildest case, so the declared value sits between the limits rather than at a favorable one. That is a bound, not a null: at N=300 per point the sweep excludes any dependence larger than about one percentage point across the full range, and a smaller dependence would not have been detected. These are relative comparisons and do not replace the N=1000 headline confirmation.

### 3.9. Matched-Control Topological Assessment

Two paired protocols were required to interpret the role of topology, and their combined outcome narrows in two steps before the boundary appears. In Protocol 1 the trivial structure was matched in resonance wavelength, loaded *Q*, peak transmission and total physical thickness and exposed to the identical 250 disorder vectors. Both retained 100% dual-mode trackability, yet the topological structure passed the complete specification in 95.2% of samples against 58.0% for the control: a paired difference of 0.372 with a 95% bootstrap interval of 0.312–0.432, 93 pairs passing only for the topological design and none only for the trivial one. Paired resampling preserves the common-disorder comparison and avoids inflating the difference through independent Monte Carlo noise [62], and the exact McNemar test on the 93 discordant pairs gives $p=2\times 10^{-28}$, so the difference is not an artifact of the sampling. The second step removes the reading that would otherwise be natural, that the advantage is extra sensitivity: at the nominal operating point the spectrally matched control is in fact about 1.19× more RI-sensitive, so whatever the comparison measures, it is not a sensitivity gain. A separately fitted RI-sensitivity-matched trivial control then supplies the boundary: under that protocol the yield difference is no longer statistically resolved, and its sign reverses. That sequence would on its own support at most a conditional, operating-point-specific reading rather than topological protection. A third step closes the sequence: an extended search found a control matched on all Protocol 1 observables and the RI sensitivity simultaneously, and against it, the difference is resolved in the control’s favor.

One control is not a family, and a single paired difference cannot separate a property of the topological operating point from a property of the particular trivial stack the fit returned. The matching conditions define an admissible set, so that set was enumerated: inside the declared search box, under the same predeclared gate on wavelength, loaded *Q*, peak transmission, total thickness and parasitic spectrum, 14 mutually distinct trivial stacks were found and each was subjected to the identical 250 disorder vectors, with admission decided by the match gate and by separation in parameter space alone and never by the paired difference. Every member favors the topological design, with paired differences from 0.268 to 0.692, a median of 0.406 and exact McNemar rejection for all 14 (largest $p=1\times 10^{-20}$; Table S9 of the Supplementary Material); the reported control sits at the 32nd percentile, so the published 0.372 is a below-median draw. Two boundaries belong with that result. The family varies the nanolaminate over the entire declared range (10.3–18.7 nm, fill 0.422–0.580), but every admissible member has its reference cavity pressed against the lower edge of the box (240–244 nm against a bound of 240), and all but one an analyte cavity above 870 nm against a bound of 900, so a wider box could hold controls this search did not reach. What the enumeration establishes is that the reported difference was not obtained against a weak opponent.

The search that had failed to find a jointly matched control was then widened because a null search inside a narrow box is weak evidence. The initial search used the Protocol 1 box with three starts of 350 evaluations. The extended search opened the analyte cavity to 200–1300 nm, the reference cavity to 150–900 nm and the coupling scale to 0.70–1.40, added the outer and central period counts as discrete variables over 3–5 and 1–3, prescreened 120 Latin-hypercube points in each of the nine period configurations and refined the four best of each by pattern search with 500 evaluations, 19,080 exact evaluations in all (Table S14 of the Supplementary Material). It found one. A trivial stack with the nominal period counts, a 375 nm analyte cavity, an 838 nm reference cavity, a 12.7 nm nanolaminate at fill 0.43 and a coupling scale of 0.94 places its resonances at 1516.0 and 1652.3 nm, with peak transmittances of 0.595 and 0.589 and a total thickness within 1.2% of the target. Its loaded quality factors are 234.2 and 205.3 when evaluated with the resonance-metric routine applied to both stacks in Table S14 of the Supplementary Material; the coarser search-stage estimator that applies the Protocol 1 gate returns 230.5 and 202.2 for the same stack. All of these values lie inside the Protocol 1 gate. The control also reproduces both local RI sensitivities of the topological stack, 104.1 and 328.2 nm/RIU, to within the resolution of the finite-difference estimator; fitted over the calibrated interval its slopes are 93.6 and 335.1 nm/RIU against 90.7 and 329.4. Its analyte overlaps, 0.098 and 0.267, match those of the topological modes, 0.092 and 0.270, which is why the sensitivities match. Under the same 250 paired disorder vectors, this control passed the complete specification in 250 of 250 samples against 239 of 250 for the topological design, one sample apart from the 238 of the Protocol 1 run: a paired difference of −0.044 with a 95% bootstrap interval of −0.072 to −0.020, 11 discordant pairs all favoring the control, and an exact McNemar $p=9.8\times 10^{-4}$. Every one of the 11 topological failures is a conditioning failure, with the scaled condition number exceeding 20, while both modes remain trackable with identity above 0.92. The reason is that the joint match does not constrain one quantity. The control’s reference cavity is 68% longer than the topological one, its reference overlaps are 0.374 and 0.185 against 0.231 and 0.124, and its temperature slopes are therefore −0.113 and −0.037 nm/°C against −0.048 and −0.016, which gives it a determinant of 34.3 against 14.3 and a scaled condition number of 3.67 against 8.20; it enters the disorder ensemble with twice the margin from the singular locus of Section 3.4 and loses fewer samples to it.

That result settles the question the two protocols had left open, and it settles it against the topological reading. Once wavelength, loaded *Q*, peak transmission, thickness and RI sensitivity are all matched, no robustness advantage of the topological junction is resolved in this comparison; the difference reverses, and the reversal is explained by conditioning, which is a property of the overlap ratios and not of the band topology. The Protocol 1 advantage was therefore a property of the Protocol 1 control family, whose members reach the target wavelengths only with a short reference cavity, weak thermal slopes and poor conditioning, and not a property of topology. What the extended search does not do is match the temperature column of S as well, which no declared protocol required; a control matched on both the RI and temperature columns of the sensitivity matrix would be the next test, and a search with one more degree of freedom would be needed to construct it. The claim retained in this paper is accordingly narrowed from a conditional specification-yield advantage to the following: at the studied operating point, the topological junction supports two identifiable interface resonances with dual-parameter functionality under the declared disorder model, and no measurable robustness benefit attributable to topology has been isolated against a control matched on the observables that determine performance.

**Figure 15:**
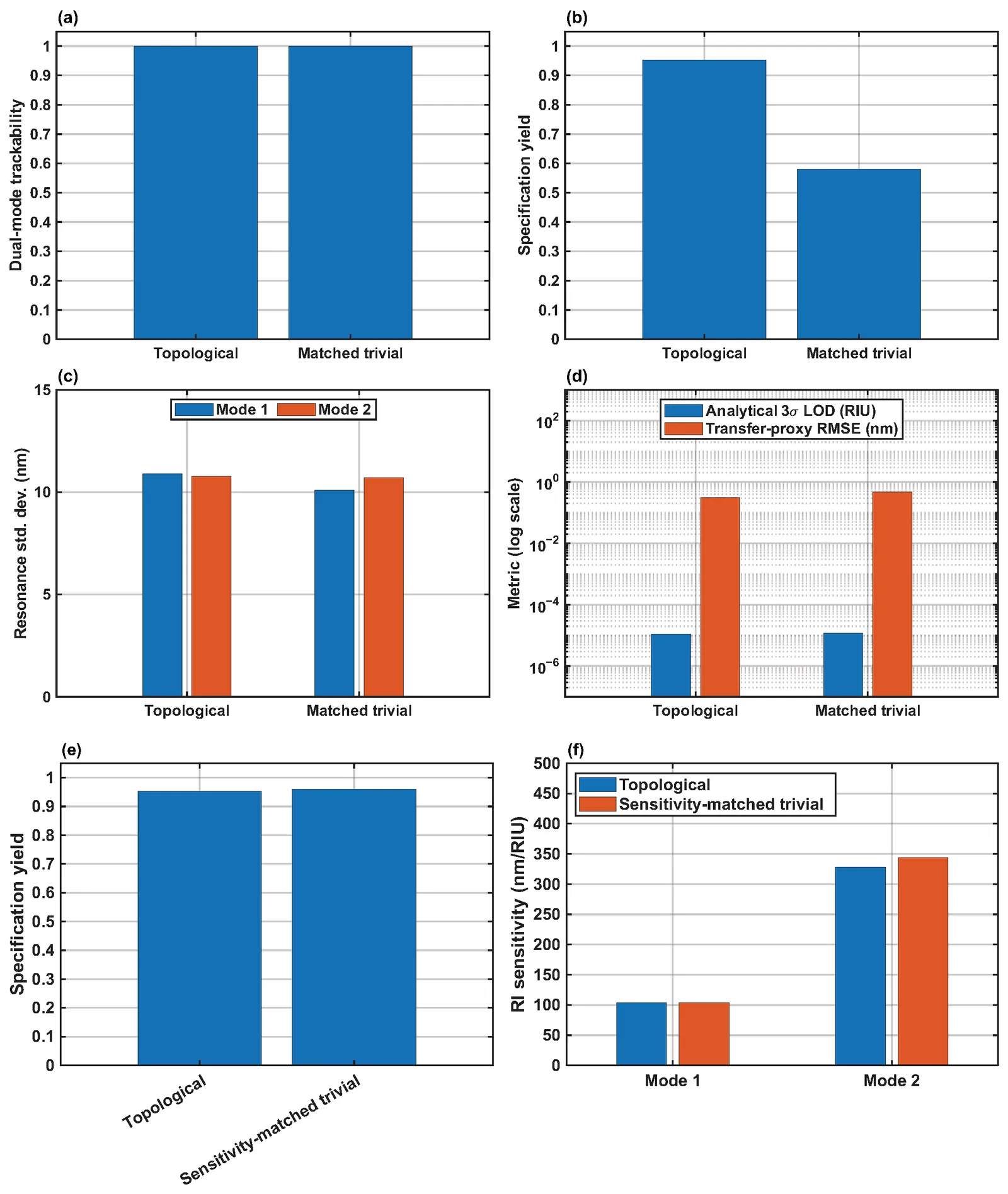
The two matched-control protocols are shown together because the second one qualifies the first. (**a**–**d**) Protocol 1, in which resonance wavelength, loaded *Q*, peak transmission, and total physical thickness are matched. (**a**) Both structures maintain perfect modal trackability, so the yield difference is not caused by missing peaks. (**b**) The complete specification yield favors the topological operating point. (**c**) The wavelength standard deviations are comparable. (**d**) The trivial control has a slightly smaller analytical LOD proxy but a larger calibration-transfer wavelength RMSE, so the advantage concerns preservation of the declared multi-metric operating point rather than a universal reduction in every error metric. (**e**,**f**) Protocol 2, which uses a separately fitted RI-sensitivity-matched trivial control that satisfies the declared spectral feasibility criterion; it is not a single control simultaneously matched on all Protocol 1 observables and RI sensitivity. (**e**) The specification yield of the topological design and of the sensitivity-matched trivial control over the same 250 paired perturbations: the bars are of nearly equal height, the paired difference is ΔY=−0.008, and its 95% bootstrap interval [−0.04,0.02] spans zero. The exact McNemar test on the 16 discordant pairs returns p=0.804, and two one-sided tests establish equivalence against a margin of five percentage points fixed in advance at one eighth of the Protocol 1 effect ($p=4.7\times 10^{-3}$). (**f**) The matching condition itself, which confirms that the trivial control reproduces both modal index sensitivities of the topological target, including the much larger sensitivity of mode 2. Within the explored control family, the Protocol 1 yield advantage is no longer statistically resolved in the separately fitted RI-sensitivity-matched control. This implicates sensitivity and modal-overlap differences as important contributors. The jointly matched control of the extended search described in the text, which matches wavelength, loaded *Q*, peak transmission, thickness and RI sensitivity at once, reverses the difference to ΔY=−0.044 with a 95% bootstrap interval of [−0.072,−0.020] (Table S14 of the Supplementary Material), so no robustness advantage attributable to topology is isolated.

Figure 15a–d decomposes the Protocol 1 result and, in doing so, excludes the three explanations that would trivialize it. Modal trackability is unity for both structures, so missing or exchanged peaks are not the source of the gap; the wavelength standard deviations are comparable for both modes, so it is not a universal suppression of resonance-position variance; and the matched trivial control can have the slightly smaller analytical RI LOD proxy while still showing the larger wavelength-transfer RMSE. The difference therefore lies in the simultaneous satisfaction of the remaining spectral, conditioning, retrieval and manufacturability gates. Under Protocol 1, the topological operating point preserves the multi-metric specification better; it does not minimize every individual error measure.

Protocol 2 uses a separately fitted RI-sensitivity-matched trivial control, and the two protocols differ in what was targeted, not in how hard each was searched: no member of the Protocol 1 family also reproduced the RI sensitivity, so Protocol 2 adds it as an explicit fitting target, and its null result is a statement about a control constructed to match sensitivity rather than a proof that no jointly matched control exists. The sensitivity-matched control reproduced the declared response to a maximum normalized mismatch of 4.87%. Under the same 250 paired perturbations, the topological design reached 0.952 against 0.960: 16 discordant pairs, with seven favoring the topological design and nine the trivial one, a paired difference of −0.008 with a 95% bootstrap interval of −0.040 to 0.024. An interval containing zero records a failure to resolve a difference, not a demonstration that none exists, so two tests are reported. The exact McNemar test returns p=0.804 (the exact form is used because 16 discordant pairs out of 250 is the regime in which the asymptotic $\chi^{2}$ version misstates the tail), and two one-sided tests against an equivalence margin of five percentage points, fixed in advance at one eighth of the Protocol 1 effect, establish equivalence at that margin ($p=4.7\times 10^{-3}$) and for any margin above 3.5 points. The data thus exclude any topological advantage above 2.4 percentage points of yield and any trivial advantage above 4.0 points, against the 37.2-point difference that survives the spectral match alone. Panels (e) and (f) verify the matching condition itself. Taken together with the jointly matched control, these results do not isolate a robustness benefit attributable to topology; they show that the Protocol 1 difference depends strongly on modal overlap and conditioning, and no universal sensitivity enhancement is claimed, in line with the broader caution that reciprocal topological photonic modes are protected only against specified perturbation classes [33,36]; in our view they should therefore be compared with controls matched in the observables that determine performance.

### 3.10. Manufacturability and Material Uncertainty

Sweeping the ITO constituent from 2.5 to 10 nm and retuning the cavities separates three quantities that fail at different rates (Figure S11 of the Supplementary Material). Resonance wavelength is not the limiting metric, since retuning recovers both wavelengths across the whole sweep. Loaded *Q* is: it holds to the declared 5 nm floor and then falls from 231.2 to 193.7 for mode 1 and from 208.3 to 185.5 for mode 2 between 5 and 10 nm, losses of 16.2% and 11.0%. The two modes also degrade unequally in sensitivity, mode 2 holding between 329.0 and 325.0 nm/RIU (1.2%) while mode 1 falls from 99.0 to 92.0 nm/RIU (7.1%), and it is that asymmetry, which erodes the channel contrast the inversion depends on, that makes the 10 nm variant fail the predefined performance-preservation flag while remaining spectrally identifiable. The 5 nm marker is a design floor used in the numerical audit, not a demonstrated physical continuity threshold.

The predeclared measured-ITO substitution tests the material model itself (Figure S14 of the Supplementary Material). Replacing the Drude curve with the on-glass ellipsometric data of Minenkov et al. shifts the resonances to 1515.201 and 1650.000 nm and gives Q=212.69 and 197.02, leaves the mode 1 slope unchanged at 100.07 nm/RIU and moves the mode 2 slope from 330.19 to 331.98 nm/RIU (0.54%), so the 3.3-fold channel separation survives; the full-covariance RI POD limits are $4.351\times 10^{-5}$ and $4.343\times 10^{-5}$ RIU, and the 300-sample specification yields are 0.98 and 0.97 with overlapping Wilson intervals (0.953–0.989 and 0.944–0.984). The sensing mechanism is therefore not an artifact of one analytic Drude curve.

Extending the substitution to all three measured films identifies the one film property the design depended on. The onset of the hyperbolic window, where Re(ε⊥) passes through zero, varies from 1102 to 1575 nm across the Drude baseline and the three ellipsometric datasets, more than 470 nm of spread for nominally the same material. Mode 1 near 1517 nm lies inside the window in three of the four cases; for the wafer-top film the onset red-shifts to 1574.75 nm, mode 1 falls below it with Re(ε⊥)=+0.486, and the optimizer as originally run rejects that film under the two-mode hyperbolicity gate. That gate is no longer part of the specification: Section 3.3 shows that $\varepsilon_{⊥}$ leaves the normal-incidence spectrum unchanged, so the rejection is withdrawn, and the condition is read only as a TM angular-robustness requirement, with the hyperbolic onset lying below the shorter resonance if oblique TM operation is intended. The wafer-bottom film satisfies that with the widest margin and is the only configuration whose window is bounded on both sides within the evaluation grid. Section S6 of the Supplementary Material lists the per-film tensor components, window edges and mode classifications.

These comparisons do not validate the exact 5 nm film because the source ellipsometry was measured on a much thicker sample [63]. Published ultrathin-ITO studies likewise show that the infrared response remains thickness-dependent [64] and that continuity below about 10 nm depends on the growth process, with critical thicknesses of about 5 nm reported for sputtered films [64] and 8–17 nm for epitaxial ones [65], and on interface defects [65].

The tolerance model deserves the same scrutiny as the films. Its widths are not measured on a deposition tool; they were chosen to sit at or beyond what the relevant processes report. A 1.0% local and 0.3% global thickness standard deviation is looser than the run-to-run control of atomic layer deposition, whose self-limiting surface reactions fix the growth per cycle at the sub-nanometre level [66], and is at the level of a monitored sputtering or evaporation run; the 0.72 layer-to-layer correlation represents process memory along the stack, and Section 3.8 shows the yield to move by only 0.013 across the whole correlation range. Interface roughness and interdiffusion enter through the same mechanism in the forward model, a graded interlayer that replaces each sharp boundary by a mixed layer with twice the RMS roughness thickness, capped at 30% of the thinner adjacent layer, and drawn per interface with mean 0.8 and standard deviation 0.35 nm in the fabrication ensemble. Sweeping that interlayer deterministically to 3 nm RMS at every interface, six times the nominal mean, moves the resonances by at most 0.08 nm and lowers the loaded *Q* by 0.35% and 0.28% (Table S13 of the Supplementary Material). An ITO/TiO_2_ interface that has interdiffused by a nanometre is therefore inside the tested range, and the reason it does not matter is structural: the nanolaminate carries 0.5–1% of the modal energy, so its internal profile is a second-order quantity. Thickness-dependent ITO properties are represented by the carrier-density and mobility scalings of the process window: specification yield stays above 0.95 for carrier densities from 0.96 to 1.3 times nominal and mobilities from 0.55 to 1.6 times nominal, a window that includes the reduced mobility ultrathin films typically show; the window was computed with the hyperbolicity gate active and is conservative for that reason, since a film failing only that gate now passes. Bi et al. report a 10 nm ITO film with a resistivity of $5\times 10^{-4}$ Ω cm [64], which implies a carrier-density–mobility product 2.6 times smaller than the Drude baseline assumed here; if that shortfall were carried entirely by the mobility, the film would fall below the 0.55 floor of the window, so the 5 nm constituent remains the least certain input of the model, as Section 4.3 states, and the nanolaminate-free recommendation of Section 3.3 removes it. Thermal hysteresis and long-term drift are not in the ensemble at all, and are bounded here instead. A hysteresis of $10^{-4}$ in the reference-coating index between heating and cooling, 0.2% of its thermo-optic excursion over the 100 K span, moves the resonances by 25 and 15 pm and is read by the inversion as an apparent change of $2.1\times 10^{-5}$ RIU and −0.48 °C, half the RI limit and four times the temperature limit; and Section 3.6 gives the uncorrected drifts that equal one detection limit, 4.0 and 14.3 pm for the RI channel and 5.9 and 1.9 pm for temperature. Both are therefore calibration-interval specifications rather than modeling choices: the reference material must be characterized for hysteresis at the $10^{-4}$ level over the operating span, and the device must be re-zeroed before its slowest drift accumulates a few picometres. Neither can be settled numerically, and both are listed among the evidence boundaries of Section 4.3.

In the broader 500-sample material-parameter ensemble, all samples produced numerical solutions and retained both modes, and 425 satisfied the hyperbolicity-inclusive gate set. That 0.850, with its 95% interval of 0.816–0.879, is a hyperbolic-classification fraction and not a usable-device yield, and it cannot be compared with the 0.959 headline yield, for two reasons. The two ensembles vary disjoint inputs: the material ensemble holds the geometry fixed and draws the constitutive parameters (ITO carrier density and mobility, effective mass, high-frequency permittivity, the mobility temperature exponent, both TiO_2_ Cauchy coefficients, the reference index and the high-index extinction coefficient), whereas the fabrication ensemble holds the constitutive model fixed apart from index-scale and thermo-optic multipliers and varies the geometry. And the gate sets differ: every one of the 75 material-ensemble failures fails on dual hyperbolicity alone, with the worst-mode loaded *Q* staying between 161.8 and 248.2 against a floor of 150, the worst-mode peak transmission between 0.337 and 0.635 against a floor of 0.30, and the scaled condition number between 7.72 and 11.09 against a ceiling of 20. With the hyperbolicity requirement removed and every other threshold being untouched, the material ensemble passes 500 of 500. Constitutive uncertainty therefore threatens no measured specification, and Section 3.3 has shown the classification it fails to be unobservable at the operating point. A standardized driver analysis identified global thickness error as the largest linear contributor to both resonance wavelengths (coefficients 0.412 and 0.480), followed by the type-*A* thickness error (about 0.342 for both modes) and the high-index material-scale error (0.309 and 0.254), which puts deposition calibration and film-specific optical characterization ahead of further nominal sensitivity optimization.

**Figure 16:**
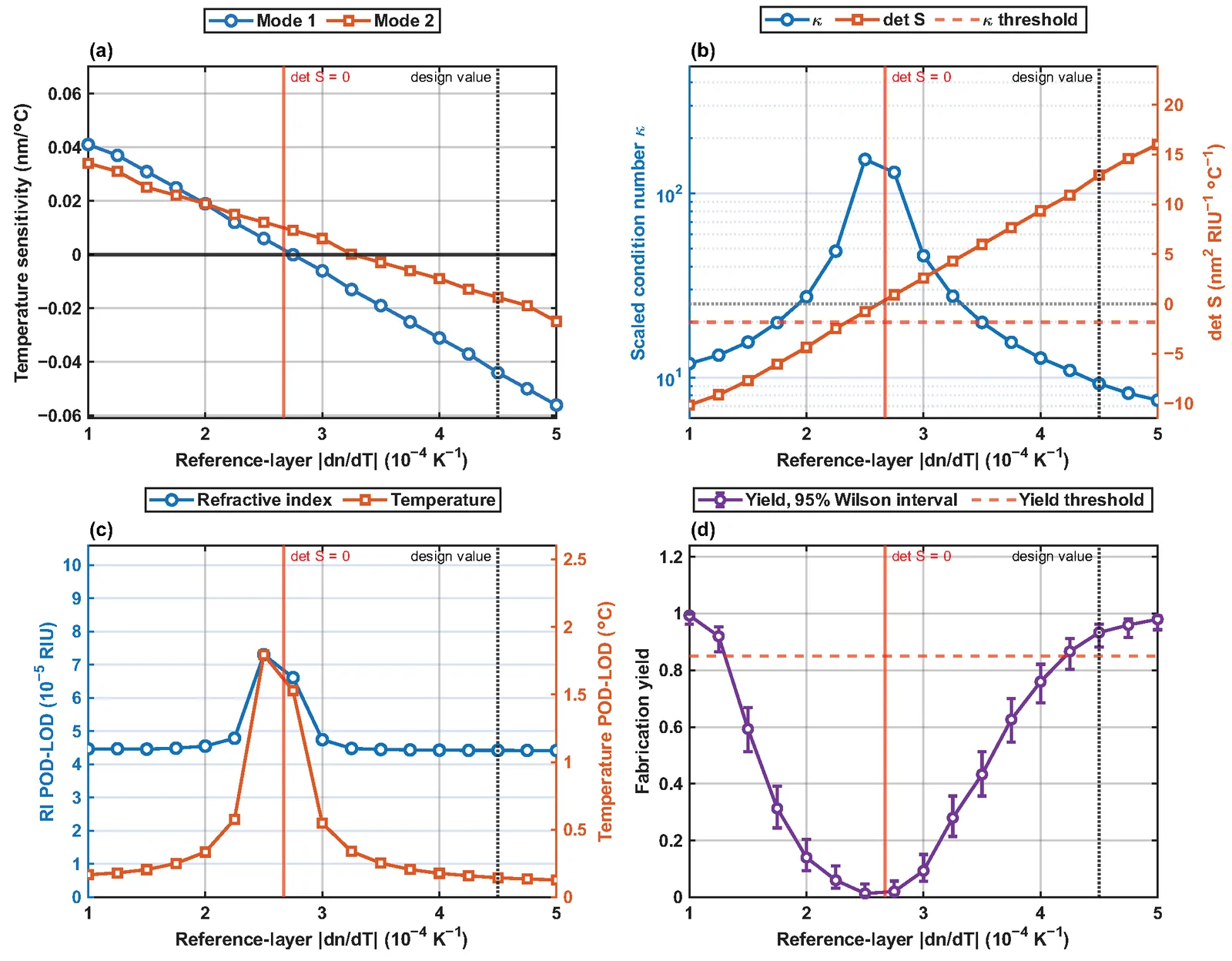
Reference-layer thermo-optic sweep. Every point re-evaluates the loaded reference design analyzed throughout this work, with only $dn_{\mathrm{ref}}/dT$ being changed; geometry, all other materials, and all thresholds stay at their frozen values, and each point carries N=150 paired Monte Carlo samples drawn from a shared seed sequence. Because the coefficient acts only through $n\left(T\right)-n\left(T_{0}\right)$, the $T_{0}$ resonance wavelengths and both RI sensitivities are invariant across the sweep, and the whole effect appears in the temperature column of S. (**a**) Temperature sensitivities of the two modes, which form that column. (**b**) Scaled condition number κ (left axis, logarithmic) and detS (right axis); the dashed line is the declared κ≤20 threshold and the grey dotted line marks detS=0. (**c**) POD detection limits for the refractive index (left axis) and temperature (right axis). (**d**) Paired fabrication yield with 95% Wilson intervals; the dashed line is the declared minimum yield of 0.85. In every panel, the dotted vertical line gives the $-4.5\times 10^{-4}$ $K^{-1}$ design value and the solid vertical line the detS=0 coefficient located by bisection at $-2.6706\times 10^{-4}$ $K^{-1}$, where the two response vectors are parallel and the measurands are inseparable. Both modes remain trackable at all 17 points: it is the inversion that fails there, not the spectrum. The sweep is supporting evidence and gates nothing.

The reference coating is the one material input whose specification the design cannot absorb. Because $dn_{\mathrm{ref}}/dT$ enters only through $n\left(T\right)-n\left(T_{0}\right)$, sweeping it leaves the $T_{0}$ wavelengths and both RI sensitivities unchanged and moves only the temperature column of S, so the sweep isolates one question: at what coefficient do the two response vectors stop being independent? Figure 16 answers this. Across $-5\times 10^{-4}$ to $-1\times 10^{-4}$ $K^{-1}$, the thermal sensitivity of mode 1 changes sign, and the determinant passes through zero at $-2.6706\times 10^{-4}$ $K^{-1}$, where the two response vectors are parallel: the scaled condition number reaches 152.9 against 7.54 at the best point, the RI POD-LOD degrades to $7.30\times 10^{-5}$ RIU, the temperature POD-LOD to 1.79 °C, and the fabrication yield falls to 0.013. Both resonances stay trackable at all 17 points, so a spectrum taken at the degenerate coefficient would look healthy, while no independent pair of parameters could be recovered from it; the detection limit, which degrades by less than a factor of two even at the singularity, is the wrong place to look for that failure, whereas the condition number rises twentyfold.

Six of the seventeen points satisfy every declared threshold, in two disjoint branches. The usable branch runs from $-4.25\times 10^{-4}$ to $-5\times 10^{-4}$ $K^{-1}$ and contains the $-4.5\times 10^{-4}$ $K^{-1}$ design value with margin on both sides. The second sits at the weak end, $-1.25\times 10^{-4}$ $K^{-1}$ and beyond, where the mechanism differs: both modes then shift positively with temperature while their RI sensitivities still differ by a factor of 3.3, so the determinant stays large. Whether that weak branch persists below $-1\times 10^{-4}$ $K^{-1}$ was not tested and is not claimed. The consequence is a materials specification rather than a tolerance: to remain on the operating branch containing the reference design, the tested results require $4.25\times 10^{-4}\leq \left|\partial n/\partial T\right|\leq 5.0\times 10^{-4}$ $K^{-1}$, the separate low-magnitude branch described above also satisfies every declared threshold, and coefficients between roughly $-1.5\times 10^{-4}$ and $-4\times 10^{-4}$ $K^{-1}$ fail at least one threshold. The forbidden interval reaches to within 11% of the design value, so this is not a remote corner of parameter space: choosing the reference material is a pass/fail decision made before fabrication, not a parameter to be trimmed afterwards.

Treating that coefficient as constant across a 100 K span is itself an idealization, so it was replaced by a secant that grows or shrinks linearly across the span at fixed value at $T_{0}$, which moves the temperature column of S and nothing else. Growth, the physical direction for a dielectric approaching its absorption edge, helps: a coefficient 50% larger at 125 °C than at 25 °C raises detS from 14.33 to 22.71 and lowers the scaled condition number from 8.20 to 5.26, and doubling it gives 31.31 and 3.91. Shrinkage is bounded: 25% weaker at the top of the span gives 10.23 and 11.43, and 50% weaker gives 6.13 and 19.04, still inside the declared ceiling of 20 but with almost no margin. The fitted quadratic term of the mode 1 wavelength surface stays below $7\times 10^{-4}$ nm/°$C^{2}$ in every case, so the bounded nonlinear calibration absorbs the curvature. The specification that matters is therefore a floor on how much the reference coefficient may weaken across the span, which this sweep places at a 50% reduction.

### 3.11. Angular and Polarization Robustness

The device is a planar stack read out in transmission, so its resonances depend on the illumination angle, and a dual-parameter readout is usable only if that dependence is small or controlled. Over the 0–15° range evaluated for both polarizations (Figure S4 and Section S10 of the Supplementary Material), the inverse problem survives: both modes stay continuously trackable, their separation never falls below 130.3 nm for TE and 132.1 nm for TM, the RI sensitivities never fall below 99.01 and 325.91 nm/RIU, and the scaled condition number rises only from 8.20 at normal incidence to 9.09 for TE and 9.22 for TM. The 15° limit is the range evaluated rather than a measured breakdown angle, so the usable angle is a lower bound. The resonances nevertheless drift by up to 21.5 nm in TE and 22.6 nm in TM across that range, against 0.33 nm for a $10^{-3}$ RIU index change on mode 2, and the maximum TE/TM splitting is 2.860 nm. Angle is therefore a first-order systematic error that the readout must fix or measure, a specification for the collimation optics rather than evidence of angular immunity; because dλ/dθ vanishes at normal incidence, a 0.02° jitter stays below the laser-reference term of the error budget only while the static misalignment is under about 0.25°, and Section 2.5 adopts ±0.1°. A matched comparison in Section S10 assigns the angular behavior to the interface geometry rather than to the nanolaminate: removing the layer changes the TE drift of mode 1 only from 8.22 to 8.28 nm and leaves the usable angle unchanged.

### 3.12. Full-Wave and Multiphysics Validation

Every number in this paper comes from one forward model, so the risk that matters is a shared implementation error that internal consistency cannot expose. Five complementary numerical formulations were therefore run against it (Table 5): a stabilized Redheffer recursion [67], which cascades the same layer matrices and audits numerical conditioning rather than independence, and four solvers that each discretize a different operator, a one-dimensional FDFD scheme [68], a three-dimensional finite-element model of the explicit 36-layer stack with Floquet ports [69], a finite-difference time-domain solve with the dispersion as auxiliary differential equations [70], and a block-iterative plane-wave eigensolver for the Bloch problem behind the topological claim [71]. The two frequency-domain solvers reproduce both resonance wavelengths to within 0.3 nm and both loaded *Q* values to within 0.5%, the time-domain solver to within 0.31 nm and 2.6%, and the eigensolver returns the π Zak-phase difference to double precision; the largest disagreement in the set is a 1.96% error in the mode 1 peak transmittance, an external-coupling quantity. None of this is experimental evidence: agreement across five formulations reduces the chance that the resonances are an artifact of one code path and says nothing about deposition defects, interface contamination, substrate effects, instrument drift or packaging. The spectral overlay of the four electromagnetic formulations (Figure S6), every grid-convergence history, the two gates applied to the time-domain material fit and the transport validation are described in Sections S8 and S11 of the Supplementary Material.

## 4. Discussion

### 4.1. Mechanism, Retrieval and What the Detection Limits Mean

The structure is a coupled two-mode resonator rather than one resonance duplicated inside a multilayer, and Section 3.4 identifies the geometric quantity that makes it a dual-parameter device: the two modes place different fractions of their energy in the analyte and in the thermo-optic reference, and it is the 5.45-fold difference between their reference-to-analyte overlap ratios, not the 3.6-fold difference in their index slopes, that keeps S invertible. The distinction matters because the two quantities can be engineered independently and because an argument written on the signs of the thermal slopes would have been reversed by the expansion-model correction described in Section S6 of the Supplementary Material, whereas the overlap-ratio argument moved by 0.95%. The audited line shape is weakly asymmetric and predominantly quasi-Lorentzian, the antisymmetric contribution reaching only 3.94% of the fitted content, so the mechanism should be attributed to coupled interface modes and overlap engineering rather than to a strong Fano resonance [49].

The nanolaminate should not be read as a sensitivity-enhancement layer. It changes phase accumulation, modal detuning, loss, splitting, external accessibility and the relative field energy in the two sensing regions at once, so the design objective it enters is multi-metric: two resolvable peaks must retain adequate sensitivity, *Q*, transmission, conditioning and fabrication tolerance simultaneously. HMM reviews and sensing studies establish the usefulness of anisotropic confinement [40,43,46]. The present results do not reproduce that usefulness at this operating point. Deleting the layer improves every metric measured here, and the hyperbolicity of what remains is invisible to a normal-incidence measurement, so neither the layer nor the sign condition on $\varepsilon_{⊥}$ can be credited with any reported performance. What survives is narrower and worth stating plainly: anisotropic loading is one of several ways to redistribute a coupled response between two sensing regions, and in this design, it was not the profitable one.

A single Jacobian does not describe the whole $1.33\leq n_{a}\leq 1.40$, 25–125 °C domain, because dispersion, thermal expansion, the temperature-dependent ITO response and the coupled-mode detuning all curve the two resonance surfaces. The bounded nonlinear inversion captures that curvature and returns held-out RMSE values of $2.43\times 10^{-5}$ RIU and 0.155 °C, roughly 47.5 dB of thermal-drift rejection in recovered index and 71.8 dB of index-drift rejection in recovered temperature. It is valid only while both resonances remain detectable and correctly identified, the measurement stays inside the calibrated domain, the local Jacobian stays nonsingular and reasonably conditioned, and the spectral-error distribution stays compatible with the uncertainty model; a broadened, mode-swapped or missing peak can invalidate the physical estimate while an optimizer converges perfectly well, which is why the prominence, linewidth, identity, residual and boundary gates are part of the retrieval method. The same physics-based surface is a transparent alternative to a learned inverse model, such as the neural-network inverse design used for nanobeam sensors [14]: the electromagnetic calculation supplies the bounded calibration and a data-driven correction can absorb repeatable fabrication or readout bias, without the training-coverage and extrapolation risks of a purely learned inverse.

The device-specific POD limits of $4.36\times 10^{-5}$ RIU and 0.123 °C are more conservative than a resolution-over-sensitivity estimate because they carry unequal and correlated peak-centre errors, nonlinear recovery, systematic bias, a 1% false-alarm target and a 95% detection target. That the full-covariance 3σ limit, the bias-aware minimum detectable change and the POD limit differ from one another is the point: “LOD” is not a unique statistic. Classical detection theory separates the decision threshold from the change detected at a stated false-negative probability [15,16], and neither is generally equal to an assumed spectrometer resolution divided by a sensitivity, so published values should be compared only after their definitions are aligned.

Where the calibration comes from matters as much as how the limit is defined. Transferring one nominal calibration to perturbed devices costs factors of 81 and 203 (Section 3.6), and the decomposition shows the whole of it is slope scatter: each device contributes an error $(S_{k}-S)x$, there is no independent zero-point term, and the population-mean mismatch enters the bias-aware MDC rather than the POD limit. Neither column of S dominates that scatter and the cross term is a third of it, so a single-point zero adjustment cannot recover the loss and a two-dimensional per-device recalibration is required. A fabricated sensor would therefore need zeroing of both wavelengths at certified index and temperature, a sparse two-dimensional calibration, confirmation of modal identity across that domain, and periodic recalibration after aging or thermal cycling. Without those steps, the headline limits do not apply; they are device-specific-calibration performance and should always be described as such. Section 3.7 quantifies how much of that per-device work is needed: the zero is indispensable, the zero plus one index step and one temperature step removes 84–93% of the excess error of a transferred calibration and reaches the per-device limits near zero, and the full surface is needed only for full-domain use. Which fabrication variations make the work necessary is also known: the global thickness error moves the zero points, the analyte-cavity thickness and the layer indices scatter the RI slopes, and the thermo-optic coefficient of the reference coating scatters the temperature slopes. The last is a material-batch property, which is why the temperature step of the per-device correction cannot be replaced by wafer metrology.

### 4.2. The Topological Contribution and the Position in the Literature

The paired matched-control analysis gives the strongest available reading of the topological contribution and also fixes its boundary. Matched on resonance wavelength, loaded *Q*, peak transmission and total physical thickness, the topological design reaches 0.952 against 0.580, a paired difference of 0.372 that holds against all 14 admissible controls enumerated. In the separately fitted RI-sensitivity-matched Protocol 2 control, the yield difference is no longer statistically resolved, with the trivial control reaching 0.960 against 0.952 with equivalence established against a pre-declared five-point margin. This implicates sensitivity and its associated modal-overlap differences as important contributors to the Protocol 1 result; the extended search of Section 3.9 then supplies the control that the two protocols lacked. Against that control, matched on wavelength, loaded *Q*, peak transmission, thickness and RI sensitivity at once, the difference reverses to −0.044 with an interval that excludes zero and with every discordant sample failing on conditioning; the Protocol 1 advantage is thereby assigned to the conditioning of that control family rather than to topology, and no robustness benefit attributable to topology is isolated. That is consistent with the restricted meaning of protection in reciprocal photonic systems, where bulk invariants constrain mode formation and connectivity but eliminate neither absorption, radiation leakage, finite-size effects, symmetry-breaking disorder nor every backscattering pathway [33,34,35], and where strong backscattering and Anderson localization have been observed experimentally in valley-Hall interface modes under realistic disorder [36]. Persistence of an in-gap peak is not by itself evidence of improved sensing performance. The defensible conclusion is that the results are consistent with the studied topological operating point retaining useful modal identities and dual-parameter functionality under the specified perturbation model, without isolating topology itself as the cause, and they also explain why an arbitrary trivial reference is inadequate: if wavelength, linewidth, optical access, field overlap or sensitivity differ, the performance difference cannot be assigned to topology at all.

Table 6 places the device among representative dual-parameter photonic sensors and among the studies closest to its coupled-topological and nanolaminate architecture. The comparison is not restricted to peak sensitivity, because sensitivity alone describes neither parameter identifiability, spectral accessibility, calibration transferability, statistical detectability nor fabrication robustness; the table therefore separates the optical metric, the retrieval or detection definition, the validation level, the robustness assessment and the use of a matched trivial control. It is descriptive rather than a ranking, since the studies use different analytes, operating domains, loss assumptions and configurations.

Several of those designs are better than this one on the metrics they report, and the paper does not claim otherwise. The coupled PC slab and dual quasi-BIC platforms reach *Q* of order $10^{5}$–$10^{6}$, the valley-Hall sensor reports 653 nm/RIU and 235 pm/°C, and the fabricated HMM platform of Sreekanth et al. reaches 30000 nm/RIU with a FOM of 590 [11,13,32,43]. On $S_{n}/\mathrm{FWHM}$, this device returns 13.86 and 41.48 ${\mathrm{RIU}}^{-1}$ against roughly $4\times 10^{4}$ ${\mathrm{RIU}}^{-1}$ for a $Q>10^{5}$ design at the same wavelength: three orders of magnitude lower. No claim of state-of-the-art sensitivity, quality factor or experimental status is made or supportable here.

What those isolated values do not settle is the two-parameter task. A large single-mode slope does not establish that two unknown perturbations can be separated, that a calibration transfers between fabricated devices, or that a stated detection probability is reached. Table 7 restates the detection-and-calibration column of Table 6 as four questions and returns the same answer to all of them: none of the compared studies defines which detection-limit statistic it reports, separates a bias-aware limit from a noise-only one, reports what happens when a calibration is transferred between nominally identical devices, or compares against a control matched on the properties that would otherwise explain the difference. That is a statement about reporting conventions rather than about the quality of the work, since most are design papers whose stated purpose is to demonstrate a structure and by that standard they succeed. The multilayer fiber-tip sensors of Vargas-Rodriguez et al., included in both tables as the closest experimental relatives of the present stack, show the same pattern from the experimental side: the forward model and the demodulation are validated on fabricated filters, and the detection statistic, the transfer of a calibration between filters and a matched control are not reported [17,18]. The gap is real nonetheless, and closing it is what this paper contributes. Sheng et al. is the sharpest case, the closest direct topological dual-parameter benchmark, with both higher nominal *Q* and survival under deliberately introduced defects [32]; this work does not supersede it on those metrics and instead answers the complementary causal question of how the apparent robustness advantage changes under progressively stricter matched-control comparisons, including the jointly matched control. Sreekanth et al. supply the experimental HMM evidence, Wei et al. the angle-tolerant HMM–TIS physics, and Majeed and Barvestani the separation and cap-layer detuning that govern coupled interface states [38,43,47]; this architecture combines those lines but remains computational, and multimethod numerical agreement is not experimental validation. The framing matches reviews identifying multiparameter operation, scalable, defect-free fabrication and experimental verification among the open challenges for topological photonic sensors [39], a gap that review draws most sharply for the non-Hermitian exceptional-point and Weyl-semimetal designs that remain primarily theoretical.

The framework also makes a falsifiable prediction about the designs in Table 6, and it runs opposite to their ranking. Per-device wavelength repeatability falls as 1/Q, with the Cramér–Rao bound on a Lorentzian centroid at fixed signal-to-noise being $\sigma_{\lambda }=\mathrm{FWHM}/\left(2\;\mathrm{SNR}\sqrt{N}\right)$, whereas the device-to-device error a transferred calibration must absorb is a bias of geometric origin that does not shrink when the resonance narrows, so the ratio of the two grows in proportion to *Q*. Anchoring the signal-to-noise product on the wavelength standard deviations reported here and the transferred limit on the factors of 81 and 203, then carrying both to the published *Q* and sensitivity values of the compared designs, gives transfer penalties between roughly $6\times 10^{3}$ and $4\times 10^{4}$ for platforms with *Q* near $10^{5}$, and $4.1\times 10^{4}$ in index and $4.4\times 10^{4}$ in temperature for the closest topological benchmark [32]. The extrapolation assumes that the compared platforms suffer the same relative fabrication spread as this one and that their per-device repeatability is linewidth-limited at comparable signal-to-noise; neither can be checked from the published reports. A high nominal *Q* buys a better per-device limit and a worse transferred one, so the two cannot be quoted independently.

### 4.3. Evidence Boundaries and the Experimental Path

The explicit-constituent agreement with EMT supports the effective medium as a design surrogate for this geometry, not as a general endorsement of local homogenization, since nanolayered HMMs can show spatial dispersion, interface effects and finite-period deviations with few constituent layers [56]. Measured-ITO substitution showed constitutive uncertainty to exceed any solver discrepancy, and the source films were thicker than the proposed 5 nm constituent and cannot validate its continuity or interfaces [63]; ultrathin ITO can remain transparent and conductive under suitable processes [64], but its response depends strongly on thickness, carrier density and mobility [64,65], and on substrate, crystallinity and interface defects [65]. The ±0.5 nm constituent sweep and the CST four-corner study retain both modes throughout the audited box, which is encouraging and is not process capability: fabrication requires thickness-specific ellipsometry, morphology by AFM or electron microscopy, sheet-resistance and preferably Hall characterization, repeated deposition runs and thermal cycling. Global thickness error dominated the wavelength variance in the material ensemble, so deposition calibration is worth more than further nominal sensitivity optimization.

Two reduced-order checks connect the optical model to a physical implementation, and both are reported in full in Section S15 of the Supplementary Material. The transport model computes Langmuir coverage in a delivery channel 50 μm high, whereas the optical model senses the bulk index of the 600 nm cavity, a factor of 83 in scale. The two describe one architecture, a channel feeding a thin sensing layer through a short vertical access. Fabricating it means releasing the cavity through etch access holes in the upper mirror, whose failure modes, stiction [72], bubble trapping and the stability of the released stack, the present calculations do not address and which are prerequisites rather than refinements. Because binding on a wall is not a change of bulk index, the transfer was computed: a 1 nm protein adlayer of index 1.53 on each cavity wall shifts the modes by 0.098 and 0.126 nm, the response is linear in coverage over 0.25–2 nm, and inverting it gives a coverage detection limit of 0.040 nm of adlayer, about 0.054 ng/mm^2^. That is a specification of the optical readout alone; the receptor chemistry, surface coverage and selectivity it assumes have not been established.

Eleven boundaries delimit the evidence. First, the study is computational: no multilayer has been fabricated and no spectrum measured. Second, the optical constants and continuity of the exact 5 nm ITO constituent are unknown. Third, the reference thermo-optic coefficient and the temperature-dependent ITO parameters are model inputs that may become nonlinear or hysteretic over 25–125 °C; Figure 16 bounds the consequence: within the tested interval, the operating branch containing the reference design requires $4.25\times 10^{-4}\leq \left|\partial n/\partial T\right|\leq 5.0\times 10^{-4}$ $K^{-1}$, a separate low-magnitude feasible branch exists near 1.0–$1.25\times 10^{-4}$ $K^{-1}$, and coefficients near $-2.5\times 10^{-4}$ $K^{-1}$ make the channels degenerate, so the reference material is a design constraint rather than a free parameter. Fourth, the POD limits require device-specific calibration and do not transfer to an uncalibrated population. Fifth, the 959/1000 yield and its Wilson interval depend on the assumed distributions, correlations and gates and are not process qualification. Sixth, the matched-control inference is conditional on the available design families and search budget: the 14-member family of Table S9 of the Supplementary Material excludes one unusually fragile control as the explanation, but every admissible member has its cavity lengths against the same edge of the declared box. The extended search of Section 3.9 widened that box and found a jointly matched control against which the advantage reverses, so the inference is now bounded by the temperature column of S, which no protocol matched. Seventh, the selected reference design is a feasibility-audited, hand-selected reference confirmed by exact Pareto recomputation, not an optimizer output and not a proven global optimum, with the primary objective flat to 8.0% over the feasible set and a surrogate holdout of $R^{2}=0.220$ over 22 held-out designs that forbids treating the surrogate as globally accurate. Eighth, the microfluidic and binding calculations are reduced-order checks without a tested receptor–analyte chemistry. Ninth, the weak resonance asymmetry does not support a strong-Fano claim. Tenth, thermal hysteresis of the reference coating and long-term drift are outside every ensemble; Section 3.10 converts them into calibration-interval requirements, a $10^{-4}$ hysteresis bound on the reference index and re-zeroing before a few picometres of drift accumulate, and neither can be settled numerically. Eleventh, the loaded stack analyzed throughout is not the configuration recommended for fabrication; the nanolaminate-free variant of Table 3 is, and while its headline figures are reported there, its calibration surfaces, control families and cross-solver validations were not rebuilt. These restrict the scope without negating the findings: the results support a reproducible computational operating point and a bounded matched-control interpretation, and they do not support a topological robustness advantage, transferable factory calibration, guaranteed yield or demonstrated biosensing.

An experimental program follows directly from that list. Material feasibility comes first, and it now has a shorter path: the recommended nanolaminate-free stack needs only the dielectric mirrors, the two cavities and the reference coating, whose thermo-optic coefficient and hysteresis should be measured over the operating span before anything else is deposited. For the loaded variant, deposit representative ITO/TiO_2_ bilayers and characterize thickness, continuity, roughness, complex optical constants, electrical properties and thermal stability. The complete multilayer should then be fabricated alongside spectrally matched and sensitivity-matched trivial controls on the same substrate and in the same deposition run, so that paired devices share wafer-scale process variation and the matched-control inference becomes experimental rather than numerical. The optical stage should measure resonance wavelength, linewidth, transmission and mode identity over a certified two-dimensional RI–temperature grid, with calibration and blind-validation fluids kept separate and repeated null and low-signal measurements used to estimate empirical covariance, bias, false-alarm frequency and POD curves. Only then should the device be extended to a biosensing application, which additionally requires controlled functionalization, receptor-density measurements, selectivity and nonspecific-binding controls, regeneration tests, fouling and drift assessment, and measurements in realistic matrices. Until those experiments exist, the system is a numerically validated RI–temperature sensing architecture and not a demonstrated biosensor.

The principal contribution is therefore neither the multilayer nor its forward model. Transfer-matrix models of thin-film stacks are settled physics, and multilayer dual-parameter sensors have been modeled, fabricated and measured before [17,18]; on nominal sensitivity and *Q*, this stack is not competitive with the designs of Table 6. What the study adds is the evaluation that those reports leave out. It defines the detection limit as a statistic, propagates a correlated wavelength covariance and a systematic bias through a bounded nonlinear calibration to reach it, and shows which instrument input each limit is a restatement of. It treats calibration as a property of the individual device, quantifies what transferring it costs and how much per-device measurement recovers the loss, and names the fabrication variations responsible. And it tests the topological claim against controls matched on the observables that determine performance, finding against the jointly matched control of Section 3.9 no robustness benefit attributable to topology, which is the strongest statement the present data support. The overlap-ratio rule of Section 3.4 and the three structural audits are what the same evaluation returned when it was turned on the design itself. Within that boundary, the results are consistent with the studied operating point maintaining identifiable modes and dual-parameter functionality under the specified perturbation model; the contribution of topology has to be evaluated against carefully matched trivial controls and cannot be inferred from the existence of an interface state.

## 5. Conclusions

The multilayer studied here is a conventional thin-film stack, and its forward model is the standard one; what this study contributes is the evaluation applied to it. This study applied a complete detection-limit framework (covariance propagation, bias-aware minimum detectable change, probability-of-detection limits, calibration transfer and matched trivial controls) to a coupled interface-mode multilayer measuring RI and temperature simultaneously, and used the same framework to audit the structural claims the device is built on. The two modes are unequally localized in the analyte and thermo-optic reference regions, which creates two distinct response vectors within a single finite multilayer. Three audits bound how that arises. The bare $U_{1}|U_{2}$ contact does carry untuned in-gap states, so the junction is topological in the sense the invariants describe, but the two modes used for sensing enter the gap only once the cavities are open and are therefore cavity-selected resonances of that junction rather than the adiabatic continuation of its bare-contact states. Deleting the ITO/TiO_2_ nanolaminate improves every metric measured here, so the layer is reported as a negative result. The hyperbolicity condition leaves the normal-incidence spectrum exactly unchanged and brings no measurable benefit under oblique TM illumination either, so it is withdrawn from the design specification, and the nanolaminate-free stack is the configuration recommended for fabrication. None of these findings weakens the detection-limit results, which is the point: the framework is what the device is a case study for.

The two resonances sit at 1516.790 and 1651.200 nm with loaded quality factors of 231.70 and 207.91, RI sensitivities of 90.71 and 329.41 nm/RIU and temperature sensitivities of −0.04780 and −0.01560 nm/°C. First-order perturbation theory ties both columns of that matrix to one geometric quantity per mode, the electric-energy overlap, and identifies the difference between the two modes’ reference-to-analyte overlap ratios as what keeps the matrix invertible; the scaled condition number is 8.20. Because the two wavelength surfaces are not planar over the operating range, both parameters were recovered by bounded nonlinear inversion, with held-out RMSEs of $2.43\times 10^{-5}$ RIU and 0.155 °C. Covariance- and bias-aware POD analysis gives device-specific detection limits of $4.36\times 10^{-5}$ RIU and 0.123 °C at a 1% false-alarm and 95% detection target; transferring one nominal calibration to perturbed devices degrades them to $3.54\times 10^{-3}$ RIU and 25.01 °C. Those limits are set by the index standard and the wavelength reference rather than by the resonance linewidth, they degrade by an order of magnitude on a compact-spectrometer readout, and on a fabricated device, they are recovered near the zero by a per-device zero plus two reference steps; the temperature they refer to is the device temperature. An independent 1000-device Monte Carlo passed 959 realizations, a 95.9% specification yield with a 95% Wilson interval of 94.5–97.0% under the stated uncertainty model.

The matched-control comparison defined the boundary of the topological claim. Matched on resonance wavelength, loaded *Q*, peak transmission and total physical thickness, the topological design reached 0.952 against 0.580, a paired difference of 0.372 with a 95% bootstrap interval of 0.312–0.432; a separately fitted RI-sensitivity-matched trivial control reached 0.960 against 0.952, a difference of −0.008 whose interval contains zero. A control found by an extended search that matches wavelength, loaded *Q*, peak transmission, thickness and RI sensitivity at once reached 1.000 against 0.956, a difference of −0.044 with a 95% interval of −0.072 to −0.020, every discordant sample failing on conditioning. What this comparison does not support is a conditional topology-attributable yield advantage: once the five declared observables are matched, no robustness benefit is isolated, and the residual difference is explained by the temperature column of the sensitivity matrix, which no protocol constrained. The results remain consistent with the studied operating point retaining useful modal identities and dual-parameter functionality under the specified perturbation model; they are neither a universal sensitivity enhancement nor unconditional fabrication immunity.

Five complementary numerical validation routes support the electromagnetic and transport models: the independent frequency-domain codes agree on the resonance wavelengths to within 0.02% and the loaded *Q* to 0.5%, the Redheffer cascade audits numerical conditioning, and the plane-wave eigensolver reproduces the bulk bands, inversion parities and π Zak-phase difference. That agreement reduces implementation-specific numerical risk; it does not replace fabrication, thickness-specific characterization of the 5 nm ITO constituent, experimental optical calibration or analyte-selectivity measurements. Future work should fabricate the topological and matched trivial structures under identical process conditions, measure their calibration surfaces and empirical detection probabilities, and determine experimentally whether any topology-specific robustness remains once the relevant spectral and sensing observables are simultaneously matched.

## Figures and Tables

**Table 1:** Frozen geometrical parameters of the selected reference design.

Parameter	Symbol	Value	Unit
TiO_2_ layer thickness	$d_{A}$	161.50	nm
SiO_2_ layer thickness	$d_{B}$	267.24	nm
Half-layer termination thickness	$d_{B}/2$	133.62	nm
Analyte-region thickness	$d_{n}$	600.00	nm
Reference-region thickness	$d_{r}$	500.00	nm
ITO thickness per nanolaminate insert	$d_{\mathrm{ITO}}$	5.00	nm
TiO_2_ thickness per nanolaminate insert	$d_{{\mathrm{TiO}}_{2},H}$	5.00	nm
Total nanolaminate-insert thickness	$d_{H}$	10.00	nm
ITO filling fraction	*f*	0.50	–
Outer periods on each side	$N_{\mathrm{out}}$	4	–
Central coupling periods	$N_{\mathrm{mid}}$	2	–
Central photonic-section length	$d_{c}$	857.48	nm
Total physical thickness	$L_{\mathrm{tot}}$	5407.40	nm
Explicit physical layers	–	36	layers

**Table 2:** Input standard deviations of the five wavelength-covariance components of Equation (20). Each is an assumed instrument specification, not a measurement. The right-hand column states how the input enters the 2×2 wavelength covariance; $S_{n}$ and $S_{T}$ are the columns of the sensitivity matrix, so any uncertainty in a physical standard is projected onto the resonances through the same slopes the retrieval uses.

Component	Input Value	Entry into $C_{\lambda }$
$C_{\mathrm{fit}}$, joint two-peak fit	350 fits	Sampled: 0.01 nm step, 0.020 nm resolution FWHM, 0.005 nm source linewidth, $10^{-3}$ detector-noise RMS, four averages
$C_{\mathrm{laser}}$, common-mode reference	$1.0\times 10^{-3}$ n m	$\sigma^{2}11^{T}$, fully correlated between modes
$C_{\mathrm{laser}}$, independent jitter	$3.0\times 10^{-4}$ n m	$\sigma^{2}I$
$C_{\mathrm{thermal}}$, stage stability	0.020 °C	$\sigma^{2}S_{T}S_{T}^{T}$
$C_{\mathrm{angle}}$, incidence jitter	0.020° RMS	Response coefficient sampled from the angle-resolved model by a 0.50° finite-difference probe about normal incidence
$C_{\mathrm{cal}}$, index standard	$1.0\times 10^{-5}$ RIU	$\sigma^{2}S_{n}S_{n}^{T}$
$C_{\mathrm{cal}}$, temperature standard	0.010 °C	$\sigma^{2}S_{T}S_{T}^{T}$
$C_{\mathrm{cal}}$, repeat averaging	5 repeats	$C_{\mathrm{fit}}/5$

**Table 3:** Complete metric set with and without the nanolaminate. The second configuration is the working stack with its two 10 nm layers deleted and every other parameter held fixed. Both columns come from the same paired sweep, whose line-shape fit returns loaded *Q* about 0.6% and 1.6% above the high-resolution spectral fit quoted elsewhere in this paper; the comparison between the columns is unaffected. Yields are specification yields under the correlated-local, systematic-global and combined-realistic fabrication scenarios, 120 samples each. The final seven rows are computed with the same covariance budget, probability-of-detection and calibration-transfer routines as the headline limits of Section 3.6; the loaded-stack entries reproduce those headline values.

Quantity	With Nanolaminate	Without	Change
Resonance wavelengths (nm)	1516.79/1651.20	1505.65/1639.79	−11.14/−11.41
Loaded *Q*	233.1/211.3	272.4/242.3	+16.9%/+14.7%
Peak transmittance	0.510/0.674	0.748/0.869	+47%/+29%
$S_{n}$ (nm/RIU)	90.70/329.42	90.45/336.06	−0.3%/+2.0%
$S_{T}$ (nm/°C)	−0.04780/−0.01560	−0.05460/−0.01792	—
detS	14.331	16.728	+16.7%
Scaled condition number	8.201	7.301	−11.0%
$S_{n}/\mathrm{FWHM}$ (${\mathrm{RIU}}^{-1}$)	13.86/41.48	16.36/49.65	+18%/+20%
Yield, systematic global	0.942	0.983	+4.1 pts
Yield, combined realistic	0.975	1.000	+2.5 pts
Yield, correlated local	1.000	0.992	−0.8 pts
Per-device $\sigma_{\lambda }$ (pm)	1.76/3.48	1.85/3.54	+5%/+2%
RI POD-LOD, per device (μRIU)	43.6	43.5	−0.2%
*T* POD-LOD, per device (°C)	0.123	0.117	−5.3%
RI POD-LOD, transferred (mRIU)	3.54	3.54	−0.1%
*T* POD-LOD, transferred (°C)	25.0	21.9	−12%
Transfer penalty, RI/*T*	81.3/203	81.3/188	0/−7%

**Table 4:** Thermal optical-path budget of the stack over a 100 K excursion, resolved by functional layer type. Entries are the change in $\sum n_{\ell }d_{\ell }$ contributed by each type, split into the part driven by ∂n/∂T and the part driven by thermal expansion. The cross term is the product of the two and is negligible everywhere except in the reference coating. A: high-index dielectric; B: low-index dielectric; H: ITO/TiO_2_ nanolaminate; AN: analyte channel; REF: thermo-optic reference coating. The 34-layer count treats each nanolaminate insert as one effective H layer; the explicit constituent representation of Table 1, in which each insert is one ITO layer and one TiO_2_ layer, contains 36 physical layers.

Region	Layers	Index (nm)	Expansion (nm)	Cross (nm)	Total (nm)
A, high-index dielectric	12	+12.920	+3.095	+0.010	+16.025
B, low-index dielectric	18	+2.672	+0.212	+0.000	+2.885
H, nanolaminate	2	+0.833	+0.015	+0.000	+0.849
AN, analyte channel	1	0	+0.399	0	+0.399
REF, reference coating	1	−22.500	+7.049	−0.225	−15.676
Whole stack	34	−6.074	+10.770	−0.214	+4.482

**Table 5:** Agreement across complementary numerical formulations. The Redheffer row cascades the same layer matrices and audits numerical conditioning; each remaining electromagnetic row discretizes a different operator, so a shared implementation error would have to reappear in an unrelated formulation to escape them. Errors are relative to the production characteristic-matrix model, per mode. The transport rows are not electromagnetic and validate the reduced-order analyte-delivery model only.

Solver	What It Discretizes	Resonance Wavelength	Loaded *Q*
Redheffer S-matrix	The same layer matrices, cascaded by a numerically stable recursion	RMSE $2.5\times 10^{-15}$ in transmittance	—
1D FDFD, 1 nm grid	Spatial Maxwell operator on a uniform grid	0.000, 0.000 nm	0.42%, 0.31%
CST FEM, 36 explicit layers	3D finite elements with Floquet ports and an independent material implementation	−0.291, +0.052 nm	0.46%, 0.21%
MEEP FDTD	Time-domain fields with the dispersion as auxiliary differential equations	0.040, 0.301 nm	2.58%, 1.90%
MPB plane-wave eigensolver	The bulk Bloch eigenproblem, parities and Zak phases	Bands to $1.5\times 10^{-5}$ in a/λ; $\Delta \theta_{\mathrm{Zak}}=\pi$ to double precision	—
FEniCSx 2D and 3D	Convection–diffusion with Langmuir kinetics; Stokes flow in a finite-width duct	NRMSE ≤2.3% on surface observables; ≤0.95% against the rectangular-duct series	—

**Table 6:** Comparison of representative dual-parameter, topological, and HMM-based photonic sensing studies with the present work. Each dual-mode sensitivity entry is written as $S_{n}/S_{T}$, with $S_{n}$ in nm/RIU and $S_{T}$ in pm/K; a semicolon separates the two modes. Exp., experimental; Num., numerical. Detection limits, demodulation deviations, and concentration thresholds use different definitions and are not directly interchangeable.

Study and Platform	Role and Measurands	Reported Sensitivities $S_{n}/S_{T}$	*Q*, FOM, or Error Metric	Calibration and Detection Assessment	Robustness, Validation, and Principal Distinction
Wang et al., three coupled PC cavities [9]	Dual RI–temperature benchmark	523/2.5; 145/60	RI and temperature deviation ratios ∼0.6% and 0.4%	Local linear 2×2 sensitivity matrix; no POD-based LOD	Numerical coupled-mode/PC analysis; no nonlinear transfer, high-sample yield, or matched control
Yang et al., coupled double PC slab [11]	High-*Q* dual RI–temperature benchmark	960/−66.5; 210/50.75	*Q* of order $10^{5}$; deviations 0.6% and 1.0%	Linear sensitivity-matrix demodulation and anti-interference analysis	TCMT and full-wave simulation; higher *Q* and RI slope, but no topology, POD, or yield analysis
Wu et al., cascaded PC nanobeam cavities [10]	Reference-channel dual sensor	210/53; 0/−90	Maximum RI and temperature deviations ∼0.56% and 3.33%	Linear matrix with a nearly RI-insensitive reference channel	3D FDTD and external-interference audit; no finite-range nonlinear calibration or fabrication yield
Gou et al., ANN-designed PC nanobeam cavity [14]	Inverse-designed dual sensor	405/40; 531/27	$Q=9.34\times 10^{4}$ and $1.55\times 10^{5}$	ANN-assisted design; no probability-based detection limit	Numerical ANN/FDTD validation; no calibration-transfer, yield, or topological-control analysis
Han et al., dual quasi-BIC PC slab [13]	High-*Q*, low-demodulation-error dual sensor	Both RI slopes reported above 700; mode-dependent $S_{T}$	*Q* near $10^{6}$ and $10^{5}$; deviations $3.1\times 10^{-6}$ RIU and 0.1 K	Resolution-based 2D sensing-matrix error; no POD-based LOD	Numerical FDTD and multipole analysis; no matched trivial control or transferred-calibration audit
Sheng et al., valley-Hall corner states [32]	Closest topological dual-parameter benchmark	176/235; 653/137	$Q>10^{5}$; maximum defect-induced peak deviation <2.64%	Linear two-state sensing matrix; no formal POD-based LOD	Numerical study with 12 defect/impurity cases; no spectrum- and sensitivity-matched trivial controls
Sreekanth et al., Au/Al_2_O_3_ HMM biosensor [43]	Experimental HMM benchmark; RI and binding	30000/– (maximum bulk RI value)	Maximum FOM 590	Experimental detection of 244 Da molecules at picomolar concentration	Fabricated microfluidic HMM; single-parameter and nontopological, but direct experimental material evidence
Wei et al., HMM-containing 1D topological heterostructure [47]	Closest HMM–TIS structural benchmark; no dual sensing	–	No sensor *Q* or LOD reported as a primary result	No RI–temperature inversion	Numerical thickness audit and TM operating angle to 45.9°; establishes angle-tolerant HMM–TIS physics
Majeed and Barvestani, coupled 1D interface states [38]	Closest coupled-TIS mechanism benchmark	204/–; preliminary estimate at M=14, no temperature channel	$Q\approx 10^{4}$, FOM ≈1300 (preliminary estimate); coupling controlled by separation and spectral alignment	Preliminary detection limit $\approx 7\times 10^{-4}$ RIU; no two-parameter retrieval or POD-based LOD	Numerical transfer-matrix study; directly supports separation/cap-layer detuning physics but has no HMM or sensing-yield framework
Vargas-Rodriguez et al., three-layer thin-film filter on a fiber tip [17]	Closest experimental multilayer dual RI–temperature benchmark	Fringe amplitude −0.20 ${\mathrm{RIU}}^{-1}$ and position 0.017 nm/RIU; position 1.04 nm/°C (simulated, one fringe, 1.30–1.31 RIU, 50– 60 °C)	No *Q* or FOM (interferometric fringes); agreement with reference liquids and a ± 1.1 °C thermocouple	Multiple-reflection model fitted to measured spectra; EMD demodulation widens the range beyond the linear sensitivity matrix; no POD-based LOD	Fabricated and measured; no calibration-transfer, yield or matched-control analysis
This work, ITO/TiO_2_-nanolaminate-loaded coupled 1D interface modes	Integrated RI–temperature framework	90.71/−47.80; 329.41/−15.60	Q=231.70 and 207.91; $S_{n}/\mathrm{FWHM}=13.86$ and 41.48 ${\mathrm{RIU}}^{-1}$; scaled κ=8.20	Bounded nonlinear inversion; RMSE $2.43\times 10^{-5}$ RIU and 0.155 °C; POD-LOD $4.36\times 10^{-5}$ RIU and 0.123 °C	N=1000 yield, measured-ITO ensemble, explicit tolerances, FDFD/CST/MEEP electromagnetic checks, MPB topology audit, FEniCSx transport validation, paired Protocol 1 spectrally matched control, a separately fitted RI-sensitivity-matched Protocol 2 control, and a jointly matched control on all five observables

**Table 7:** Reporting practice in the compared studies, restated from the detection-and-calibration assessments of Table 6. Entries record what each cited work reports, not what it could have reported. An entry of “no” records that the statistic behind a limit is not defined rather than that no number is quoted: Han et al. propagate assumed spectral resolutions of $2.8\times 10^{-4}$ and $3.1\times 10^{-3}$ nm through the inverse sensing matrix, and Majeed and Barvestani give a preliminary limit of $7\times 10^{-4}$ RIU, neither with a stated noise model or confidence level.

Study	LOD Statistic Defined	Bias-Aware Limit	Calibration Transfer	Matched Control
Wang et al. [9]	no	no	no	no
Yang et al. [11]	no	no	no	no
Wu et al. [10]	no	no	no	no
Gou et al. [14]	no	no	no	no
Han et al. [13]	no	no	no	no
Sheng et al. [32]	no	no	no	no
Sreekanth et al. [43]	experimental only	no	no	no
Wei et al. [47]	not applicable	no	no	no
Majeed and Barvestani [38]	no	no	no	no
Vargas-Rodriguez et al. [17]	no	no	no	no
This work	yes	yes	yes	yes

## Data Availability

The data presented in this study are archived in Zenodo at https://doi.org/10.5281/zenodo.21879672 and will be openly available upon publication. Version 1.2.0 contains the simulation code, frozen material, geometry and acceptance-criterion inputs, the cross-solver verification models, and the numerical series behind every figure and table. Data are released under CC BY 4.0 and code under the MIT licence. No experimental data were generated in this study.

## Supplementary Materials

The following supporting information can be downloaded at: https://www.mdpi.com/article/10.3390/s26185955/s1, a single Supplementary Material document containing Sections S1–S15 with the extended methods and modeling details, the predeclared numerical acceptance criteria and audit appendices, and the supplementary results, including Figures S1–S17 and Tables S1–S15.

## Abbreviations

The following abbreviations are used in this manuscript:

AFM	Atomic force microscopy
ANN	Artificial neural network
BIC	Bound state in the continuum
CI	Confidence interval
CMT	Coupled-mode theory
CPU	Central processing unit
CST	CST Studio Suite (full-wave solver)
EMT	Effective-medium theory
FDFD	Frequency-domain finite difference
FDTD	Finite-difference time-domain
FOM	Figure of merit
FWHM	Full width at half maximum
GPU	Graphics processing unit
HMM	Hyperbolic metamaterial
ITO	Indium tin oxide
LOD	Limit of detection
MAD	Median absolute deviation
MAPE	Mean absolute percentage error
MDC	Minimum detectable change
MEEP	MIT Electromagnetic Equation Propagation
MPB	MIT Photonic Bands
MPI	Message passing interface
NRMSE	Normalized root-mean-square error
PC	Photonic crystal
PDMS	Polydimethylsiloxane
POD	Probability of detection
RI	Refractive index
RIU	Refractive index unit
RMS	Root mean square
RMSE	Root-mean-square error
SUPG	Streamline-upwind Petrov–Galerkin
TCMT	Temporal coupled-mode theory
TE	Transverse electric
TIS	Topological interface state
TM	Transverse magnetic
TMM	Transfer-matrix method
