# Supplementary material for: Bias-Aware Detection Limits, Calibration Transfer and Matched-Control Robustness Assessment in Dual-Parameter Photonic Refractive-Index and Temperature Sensing: A Coupled Interface-Mode Multilayer Case Study
Source: Sensors (Basel). 2026 Sep 20;26(18):5955. doi: 10.3390/s26185955 (PMC13611379; doi:10.3390/s26185955)
Supplement: Supplementary file 1 [file sensors-26-05955-s001.zip › sensors-4526680-supplementary.pdf]

Supplementary Material

Bias-Aware Detection Limits, Calibration Transfer and Matched-Control Robustness Assessment in Dual-Parameter Photonic Refractive-Index and Temperature Sensing: A Coupled Interface-Mode Multilayer Case Study

Agah Oktay Ertay   Muhammed Mustafa Ertay

This document collects the material that supports the article without being needed to follow its argument: the full constitutive and numerical formulations, the optimisation algorithm and its budgets, the convergence and cross-solver audits, the frozen parameter tables, and the transport–reaction models. Section numbers referenced from the article as Sn refer to this document. Every number here comes from the same single execution of the analysis code that produced the article, and the tables are written directly by that code.

Contents

S1 Constitutive Models . . . 2
- S1.1 Dielectric and Thermo-Optic Models . . . 2
- S1.2 ITO Drude and Measured Optical Constants . . . 4
- S1.3 Effective-Medium Model of the HMM . . . 5

S2 Electromagnetic Formulation . . . 5
- S2.1 Stabilized Scattering-Matrix Formulation . . . 5

S3 Band Topology of the Two Half-Crystals . . . 6
- S3.1 Bulk Band Structure and Bloch Dispersion . . . 6
- S3.2 Parity Eigenvalues and Zak Phase . . . 6
- S3.3 Reflection-Phase Criterion and Bulk–Boundary Correspondence . . . 7

S4 Design Search and Monte Carlo Model . . . 7

S5 Independent Numerical Validation Models . . . 10
- S5.1 Computational Environment and Runtime . . . 10
- S5.2 FDFD Model . . . 11
- S5.3 CST Full-Wave Model . . . 11
- S5.4 FDTD Model . . . 11
- S5.5 Plane-Wave Eigensolver Model . . . 12
- S5.6 Finite-Element Transport–Reaction Model . . . 12
- S5.7 Finite-Element 3D Hydrodynamic Model . . . 13

S6 Model Parameters . . . 13

S7 Numerical Acceptance Criteria . . . 14

S8 Solver and Convergence Details . . . 16
- S8.1 Scalar Redheffer Star Product . . . 16
- S8.2 FDFD Grid Convergence . . . 16
- S8.3 CST Full-Wave Model . . . 16
- S8.4 FDTD Model . . . 16
- S8.5 Plane-Wave Eigensolver Audit . . . 17
- S8.6 Finite-Element Transport–Reaction and Hydrodynamic Models . . . 17

S9 Narrative Summary Figures . . . 18

S10 Angular and Polarization Behaviour . . . 19

S11 Independent Numerical Validation in Detail . . . 21
- S11.1 Independent FDTD Validation . . . 23
- S11.2 Independent Topological Eigensolver Audit . . . 25

S12 Determinant of the Sensitivity Matrix from the Modal Overlaps 27
S13 Supporting Results Floats 29
S14 Robustness of the Reported Results to the Declared Assumptions 33
S14.1 Instrument-Noise and Drift Scenarios 33
S14.2 Reduced Per-Device Calibration 33
S14.3 Oblique-Incidence Test of the Hyperbolicity Condition 33
S14.4 Interface Roughness and Interdiffusion Sweep 33
S14.5 Jointly Matched Trivial Control 36
S14.6 Drivers of the Sensitivity-Slope Scatter 36
S15 Microfluidic Integration and Surface-Binding Transfer 37

S1 Constitutive Models

S1.1 Dielectric and Thermo-Optic Models

Each isotropic material j is represented by a complex refractive index and relative permittivity,

$$\widetilde{n}_j(\lambda, T) = n_j(\lambda, T) + \mathrm{i}\kappa_j(\lambda, T), \qquad \varepsilon_j(\lambda, T) = \widetilde{n}_j^2(\lambda, T). \tag{S1}$$

Material dispersion is evaluated at each wavelength. The frozen near-infrared reference values at $T_0 = 25\,^\circ\mathrm{C}$ are $n_{\mathrm{TiO_2}} = 2.40$, $n_{\mathrm{SiO_2}} = 1.4440$, and $n_\mathrm{r} = 1.41$; the dispersion models that produce them, together with the extinction coefficients and the thermal coefficients repeated for convenience, are tabulated in Table S4. Small extinction coefficients are retained in the calculation rather than forcing the dielectric layers to be exactly lossless.

Over the declared temperature interval, the first-order thermo-optic and linear-expansion laws are

$$n_j(\lambda, T) = n_j(\lambda, T_0) + \alpha_{n,j}(\lambda)(T - T_0), \tag{S2}$$
$$d_j(T) = d_j(T_0)\left[1 + \alpha_{d,j}(T - T_0)\right], \tag{S3}$$

where $\alpha_{n,j} = \partial n_j/\partial T$ and $\alpha_{d,j}$ is the linear thermal-expansion coefficient. The coefficients used in the frozen model are listed in Table S1. The reference layer is treated as an idealized thermo-optic design material with $\partial n/\partial T = -4.5 \times 10^{-4}$ K^{-1}, chosen so that the reference-region thermal response is rotated relative to the analyte-RI response. No specific commercial polymer is claimed, and no film-specific value is adopted for the design coefficient itself. Optical polymers are nevertheless the natural material family for such a layer, and both their thermo-optic coefficients and the wavelength dispersion of those coefficients have been measured systematically for waveguide applications [1, 2]. Polydimethylsiloxane is a concrete example, and the individual claims are supported separately. The wavelength-dependent refractive index and attenuation of commonly used PDMS formulations have been characterized for optical microsystems [3]; extrapolating their single-term Sellmeier fit to 1550 nm gives $n \approx 1.42$, close to the $n_\mathrm{r} = 1.41$ used here. PDMS-based hybrid photonic devices have separately demonstrated a large negative thermo-optic response and experimental thermal tuning between 20 °C and 75 °C [4], which indicates an application-relevant interval rather than the extended stress-test range discussed below. Experimental implementation will require replacement of this design coefficient by measured $n(\lambda, T)$, extinction coefficient, thermal-stability, and glass-transition data for a selected material, together with the corresponding primary characterization references; the sensitivity matrix and detection limits would then be recomputed on that physical footing.

Because that coefficient is assumed rather than measured, its influence is quantified directly instead of being left to the reader. An additional sweep varies $\partial n/\partial T$ over 17 values from -1×10^{-4} to -5×10^{-4} K^{-1} with the geometry, every other material, and every acceptance threshold frozen at the loaded reference design analysed throughout this work, and re-evaluates each point with the same routines that generate the conditioning, detection-limit, and fabrication-yield results. Fabrication yield uses $N = 150$ Monte Carlo realizations per point drawn from one shared seed sequence, so the comparison across coefficients is paired. Since $\partial n/\partial T$ acts

only through $n(T) - n(T_0)$, the resonance wavelengths at T_0 and the entire RI column of $\mathbf{S}$ are numerically identical at all 17 points, and the whole effect appears in the temperature column. Note that this sweep evaluates $\mathbf{S}$ as a local finite-difference Jacobian at the nominal operating point, whereas the matrix reported in Equation (29) of the article is obtained from regression slopes over the full calibration span. Because the thermal response is not strictly linear across that span, the two estimators disagree by 10% on the determinant, with the largest single-entry difference also 10%, on $S_{n,1}$; the two temperature entries agree to 8% and 2%. The sweep is therefore read as a comparison at fixed estimator rather than as a restatement of the reported sensitivity matrix.

The sweep shows that performance is not monotone in the reference coefficient. As $|\partial n/\partial T|$ decreases, $S_{T,1}$ rises from -0.056 to $+0.041$ nm/°C and its ratio to $S_{n,1}$ climbs toward that of mode 2. Because $\det(\mathbf{S}) = S_{n,1}S_{n,2}(S_{T,2}/S_{n,2} - S_{T,1}/S_{n,1})$, the determinant in Equation (15) of the article passes through zero where the two thermal-to-index ratios coincide. That crossing is not the coefficient at which $S_{T,1}$ changes sign: at $S_{T,1} = 0$ the determinant still equals $S_{n,1}S_{T,2} = 0.90$, so the loss of the sign difference and the loss of identifiability are separate events. Bisection places that degeneracy at $\partial n/\partial T = -2.67 \times 10^{-4}$ K^{-1}, where the RI and temperature response vectors are parallel and the two parameters cannot be separated at any noise level. Approaching it, the scaled condition number rises to 152.9, the RI detection limit degrades to 7.3×10^{-5} RIU, the temperature limit to 1.79 °C, and the fabrication yield falls to 0.013. The exact location of the degeneracy depends on which of the two $\mathbf{S}$ estimators is used; its existence and approximate position do not.

Six of the 17 coefficients satisfy every acceptance threshold. Four of them form a contiguous branch from -4.25×10^{-4} to -5×10^{-4} K^{-1} on which conditioning and both detection limits improve monotonically with $|\partial n/\partial T|$, and which contains the design value with margin on either side: at -4.5×10^{-4} K^{-1} the scaled condition number is 9.27 and the yield 0.933, against 7.54 and 0.980 at -5×10^{-4} K^{-1}. The other two are the weakest coefficients tested, -1.25×10^{-4} and -1×10^{-4} K^{-1}, which are well conditioned for a different reason: both modes then shift positively with temperature, but their RI sensitivities still differ by a factor of 3.3, so the determinant remains large. Whether that branch persists below -1×10^{-4} K^{-1} was not tested. The practical consequence is a materials specification rather than a tolerance. Within the tested interval, a reference coating on the high-magnitude branch, $4.25 \times 10^{-4} \le |\partial n/\partial T| \le 5.0 \times 10^{-4}$ K^{-1}, reproduces the reported operating mechanism, and the separate low-magnitude branch described above also satisfies the declared thresholds through a different response orientation, whereas coefficients between roughly -1.5×10^{-4} and -4×10^{-4} K^{-1} fail at least one threshold and must be avoided. Per-point results are reported in Section 3.10 of the article; the underlying per-point table is included in the archived reproducibility package cited in the Data Availability Statement.

Table S1: Thermo-optic and thermal-expansion inputs at the reference temperature.

Material	α_n (K^{-1})	α_d (K^{-1})
TiO_2	8.0×10^{-5}	8.0×10^{-6}
SiO_2	1.0×10^{-5}	5.5×10^{-7}
Thermo-optic reference	-4.5×10^{-4}	1.0×10^{-4}

The analyte has the nominal index $n_{\mathrm{a},0} = 1.33$. No specific chemical species is claimed: the interval covers aqueous solutions from pure water to concentrated sugar or protein solutions, and the model treats the analyte as a transparent dielectric of prescribed index. Two consequences of that idealization are worth quantifying rather than leaving implicit. First, the operating wavelengths sit on the long-wavelength side of the 1450 nm water absorption band, where the extinction coefficient of water is of order $k \simeq 8 \times 10^{-5}$. With the analyte energy fractions computed in Section 3.4 of the article, 0.092 for mode 1 and 0.270 for mode 2, the absorption-limited quality factor $Q_{\mathrm{abs}} = n/(2k\Gamma_{\mathrm{a}})$ is 9.0×10^4 and 3.1×10^4 respectively. Both exceed the loaded quality factors of 232 and 208 by more than two orders of magnitude, so analyte absorption is negligible against the radiative and material losses already in the model. Second, an absorbing or dispersive analyte would change this conclusion, and a species whose absorption

band overlaps either resonance would have to be modelled explicitly. RI and temperature are treated as independent inverse variables over

$$1.33 \le n_\mathrm{a} \le 1.40, \qquad 25\,^\circ\mathrm{C} \le T \le 125\,^\circ\mathrm{C}. \tag{S4}$$

An application-specific relation $n_\mathrm{a}(T)$ may later be incorporated as prior information, but it is deliberately not imposed here because doing so would reduce the dimensionality of the dual-parameter problem.

The 25 °C to 125 °C interval is used solely as an *extended numerical stress test* and should not be interpreted as the operating range of a specific analyte, assay, or reference polymer. It is chosen so that (i) the nonlinear two-dimensional calibration is exercised over a full 100 °C span, allowing the curvature and RI–temperature interaction of the nonlinear calibration surfaces defined in Equation (17) of the article to become numerically resolvable; (ii) the ITO temperature-dependent Drude parameters, the thermo-optic coefficients of TiO_2, SiO_2, and the reference layer, and the interface thermal expansion are simultaneously driven far enough from the reference temperature to expose the limits of the first-order approximations used in Equations (S2)–(S3); and (iii) the covariance- and bias-aware POD framework is evaluated under a demanding thermal-drift budget that upper-bounds any narrower application-specific interval. Within this stress-test view, the nonlinear retrieval RMSE and POD analyses are evaluated over the declared calibration domain, whereas the reported sensitivity matrix represents local derivatives at the nominal operating point; narrower application-specific intervals would yield tighter local metrics. Extending the reduced analytic thermo-optic and Drude models beyond 125 °C, or into a range where the reference-layer glass transition or ITO annealing regime becomes relevant, is left outside the present numerical scope.

S1.2 ITO Drude and Measured Optical Constants

The baseline dispersive ITO response is modeled by

$$\varepsilon_\mathrm{ITO}(\omega, T) = \varepsilon_\infty - \frac{\omega_p^2(T)}{\omega^2 + \mathrm{i}\gamma(T)\omega}, \tag{S5}$$

with $\varepsilon_\infty = 3.9$, $\omega_{p,0} = 2.65 \times 10^{15}$ rad s^{-1}, and $\gamma_0 = 1.20 \times 10^{14}$ rad s^{-1}. The temperature increments used in the frozen model are

$$\omega_p(T) = \omega_{p,0}\left[1 + \eta_p(T - T_0)\right], \qquad \gamma(T) = \gamma_0\left[1 + \eta_\gamma(T - T_0)\right], \tag{S6}$$

where $\eta_p = -1.0 \times 10^{-4}$ K^{-1} and $\eta_\gamma = 1.0 \times 10^{-3}$ K^{-1}. The model also carries an equivalent carrier-density, effective-mass and mobility representation, constrained to reproduce the frozen room-temperature Drude parameters; the complete set, including those derived values, is listed in Table S5.

Because ITO optical constants depend strongly on thickness, deposition method, oxygen content, carrier density, annealing, substrate, roughness, and film continuity, the Drude curve is not used as the sole material-realism check. The complete sensor calculation is repeated with ellipsometrically measured n and κ data reported by Minenkov et al. [5]. The on-glass data set was declared in advance as the primary measured-data substitution because its substrate configuration was the closest available match to the intended stack; top- and bottom-wafer data are retained as an optical-constant ensemble. The measured room-temperature values are interpolated only within their supported wavelength interval, and the declared Drude temperature increment is applied separately rather than inventing unmeasured temperature-dependent ellipsometric data.

The provenance of these third-party data is as follows. All three data sets (the 110 nm on-glass film and the top- and bottom-sublayer constants of the 88 nm on-wafer film) were obtained as tabulated numerical values and were not digitised from a published figure: they were downloaded from the In_2O_3–SnO_2 (Minenkov) entry of the refractiveindex.info database [6], which redistributes the ellipsometric constants reported in the source study [5]. Each file holds 706 wavelength points spanning 191.46 nm to 1688.69 nm. The only modification applied was conversion of the wavelength column from micrometres to nanometres; the tabulated n and κ values are

used unaltered. They are placed on the simulation wavelength grid by shape-preserving piecewise cubic Hermite interpolation. The Drude-to-measured comparison is confined to the overlap between the 1450 nm to 1720 nm evaluation window and the measured support, that is 1450 nm to 1688.69 nm, containing 69 tabulated points. No value extrapolated beyond the measured support enters a root-mean-square error, a resonance solve, or a hyperbolic-window edge, and any window edge that reaches the support limit is reported as a lower bound rather than as a measured edge, as marked in Table S6. The database is released under a CC0 1.0 universal public-domain dedication.

The measured source film is substantially thicker than the proposed 5 nm constituent. Reports of ultrathin ITO demonstrate that infrared transparency and electrical continuity can persist in some process-specific films, but also show strong thickness dependence [7, 8] and, below about 10 nm, a loss of metallicity through interface defects [8]. These studies support treating an ultrathin implementation as physically investigable; they do not validate the exact 5 nm ITO layer assumed here. Film-specific ellipsometry, AFM, continuity, conductivity, and thickness measurements remain necessary before fabrication claims can be made.

S1.3 Effective-Medium Model of the HMM

For high-throughput design and optimization, an ITO/TiO_2 pair is first represented by a local uniaxial effective medium whose optical axis is normal to the constituent layers. The approximation assumes planar interfaces and a constituent period small relative to the wavelength and the local field-variation length. It is therefore used as a surrogate material representation, not as a claim that one bilayer is microscopically homogeneous. Local effective-medium theory can lose accuracy for a small number of periods, strong spatial dispersion, or appreciable interface nonlocality [9].

The design hierarchy is: (i) optimization, sensitivity-matrix construction, nonlinear two-dimensional calibration, uncertainty propagation, POD-based limit-of-detection analysis, and the $N = 1000$ Monte Carlo yield confirmation are performed using the validated uniaxial effective tensor of the 5 nm ITO/5 nm TiO_2 physical design; (ii) the selected design is subsequently audited by replacing both HMM slabs with their explicit 5 nm/5 nm constituents and re-solving the spectrum and field profiles; and (iii) the explicit constituent stack is used for independent FDFD and CST full-wave validation. The effective-permittivity equations and hyperbolic classification are given in Section 2.3.2 of the article; the numerical EMT-to-explicit comparison is reported in Section 3.2 of the article. Headline sensing figures of merit are not obtained by re-running the full optimization or uncertainty pipeline directly on the explicit stack.

S2 Electromagnetic Formulation

S2.1 Stabilized Scattering-Matrix Formulation

At an interface from medium i to medium j, admittance-form field coefficients are

$$r_{ij} = \frac{Y_i - Y_j}{Y_i + Y_j}, \qquad t_{ij} = \frac{2Y_i}{Y_i + Y_j}. \tag{S7}$$

With incoming amplitudes a_i^+ and a_j^- and outgoing amplitudes a_i^- and a_j^+, the interface is written as

$$\begin{bmatrix} a_i^- \\ a_j^+ \end{bmatrix} = \underbrace{\begin{bmatrix} r_{ij} & t_{ji} \\ t_{ij} & r_{ji} \end{bmatrix}}_{\mathbf{S}_{ij}} \begin{bmatrix} a_i^+ \\ a_j^- \end{bmatrix}. \tag{S8}$$

Propagation through a homogeneous layer of thickness d_j uses the passive factor $p_j = \mathrm{e}^{\mathrm{i}q_j d_j}$ and the two-port block

$$\mathbf{S}_j^{(p)} = \begin{bmatrix} 0 & p_j \\ p_j & 0 \end{bmatrix}. \tag{S9}$$

The interface and propagation blocks are cascaded using the Redheffer star product [10, 11]:

$$\mathbf{S}_{\text{tot}} = \mathbf{S}_1 \star \mathbf{S}_2 \star \cdots \star \mathbf{S}_N. \tag{S10}$$

The star product analytically eliminates internal incoming and outgoing amplitudes while retaining the external scattering relation. In contrast to unrestricted transfer-matrix multiplication, it does not explicitly propagate exponentially increasing factors associated with passive evanescent waves. It consequently avoids overflow and severe loss of precision in long, lossy, high-Q, or strongly evanescent stacks. Numerical guardrails enforce passive branch selection, reciprocity, finite amplitudes, energy balance in lossless tests, spectral-boundary clearance, and continuous mode identity. The complete scalar star product is written out in S8.

Two independent cascade implementations are retained, and their roles must not be confused. Production spectra, resonance metrics, sensitivity matrices, nonlinear calibration, and all Monte Carlo ensembles are evaluated with the characteristic-matrix (transfer-matrix) cascade, which is the faster route for the many millions of spectral evaluations these studies require. The stabilized Redheffer star-product implementation described above is evaluated separately and is the numerical-conditioning reference against which the transfer-matrix results are audited; over the operating band the two agree to a transmittance RMSE of 2.5×10^{-15} for the effective-medium stack and 2.6×10^{-15} for the explicit constituent stack, so the choice of cascade does not affect any reported quantity. Section 3.12 of the article reports the conditioning audit, including the loss-scale and period range within which unrestricted transfer-matrix multiplication remains safe.

S3 Band Topology of the Two Half-Crystals

S3.1 Bulk Band Structure and Bloch Dispersion

The infinite periodic crystals are evaluated independently of the finite sensor. For a unit cell of period $\Lambda = d_A + d_B$, Bloch fields satisfy

$$\mathbf{E}_{nK}(z + \Lambda) = \mathrm{e}^{\mathrm{i}K\Lambda}\mathbf{E}_{nK}(z). \tag{S11}$$

The bulk eigenproblem is solved in a plane-wave basis [12],

$$\nabla \times \left[\mu^{-1}(\mathbf{r})\nabla \times \mathbf{E}_{nK}(\mathbf{r})\right] = \frac{\omega_{nK}^2}{c^2}\varepsilon(\mathbf{r})\mathbf{E}_{nK}(\mathbf{r}). \tag{S12}$$

As an independent one-dimensional audit, let $\mathbf{M}_{\text{uc}} = \left[\begin{smallmatrix} A & B \\ C & D \end{smallmatrix}\right]$ be the unit-cell transfer operator reconstructed from its stable scattering representation. For a reciprocal unit cell with unit determinant,

$$\cos(K\Lambda) = \frac{A + D}{2}. \tag{S13}$$

In the lossless limit, $|(A + D)/2| \leq 1$ defines an allowed band and $|(A + D)/2| > 1$ defines a band gap. With absorption, K becomes complex and the gap is identified jointly from $\mathrm{Im}(K)$ and the finite-stack scattering spectrum. Calculated bands and gaps are reported in Section 3.1 of the article.

S3.2 Parity Eigenvalues and Zak Phase

For an isolated one-dimensional band, the Zak phase is [13]

$$\theta_n^{\text{Zak}} = \mathrm{i}\int_{-\pi/\Lambda}^{\pi/\Lambda} \left\langle u_{nK} \middle| \frac{\partial u_{nK}}{\partial K} \right\rangle \mathrm{d}K \pmod{2\pi}, \tag{S14}$$

where u_{nK} is the periodic Bloch function. Numerically, a gauge-invariant product of neighboring eigenvector overlaps is used:

$$\theta_n^{\text{Zak}} = -\,\mathrm{Im}\ln \prod_m \frac{\langle u_{nK_m} | u_{nK_{m+1}} \rangle}{|\langle u_{nK_m} | u_{nK_{m+1}} \rangle|}. \tag{S15}$$

When the unit cell is inversion symmetric, the origin is placed at an inversion center, and the band is isolated, the phase is quantized to 0 or π. It can then be cross-checked using the parity eigenvalues $\xi_n = \pm 1$ at the inversion-invariant momenta:

$$e^{i\theta_n^{\mathrm{Zak}}} = \xi_n(0)\xi_n(\pi/\Lambda). \tag{S16}$$

The overlap and parity calculations are required to agree after K-grid refinement. The physically relevant invariant of a gap is formed from the cumulative phases of all bands below that gap. Because a translated inversion center can shift individual Zak phases, the interpretation is always tied to the actual termination of the finite stack.

S3.3 Reflection-Phase Criterion and Bulk–Boundary Correspondence

The bulk diagnosis is connected to the finite device through the reflection coefficients of the terminated left and right crystals,

$$r_{\mathrm{L}} = |r_{\mathrm{L}}|e^{i\phi_{\mathrm{L}}}, \qquad r_{\mathrm{R}} = |r_{\mathrm{R}}|e^{i\phi_{\mathrm{R}}}. \tag{S17}$$

For a direct ideal interface in a common lossless gap, an interface state satisfies

$$r_{\mathrm{L}}r_{\mathrm{R}} = 1, \qquad \phi_{\mathrm{L}} + \phi_{\mathrm{R}} = 2m\pi, \quad m \in \mathbb{Z}. \tag{S18}$$

For the present finite coupling section, the more general round-trip condition is

$$r_{\mathrm{L}}r_{\mathrm{R}}e^{2ik_{\mathrm{c}}d_{\mathrm{c}}} = 1, \tag{S19}$$

or, in phase form,

$$\phi_{\mathrm{L}} + \phi_{\mathrm{R}} + 2\,\mathrm{Re}(k_{\mathrm{c}})d_{\mathrm{c}} = 2m\pi. \tag{S20}$$

The connection between the cumulative Zak phase, surface impedance, and reflection-phase sign provides the one-dimensional bulk–boundary interpretation [14, 15]. In finite lossy stacks, $|r|$ need not equal unity and the state has a finite linewidth. It is therefore located from a pole-compatible resonance of the complete complex scattering response rather than by imposing Equation (S18) exactly.

S4 Design Search and Monte Carlo Model

The design search combines 76 unique discrete topology/termination configurations with ten bounded variables: d_A, d_B, d_{n}, d_{r}, d_H, f, N_{out}, N_{mid}, mirror imbalance, and a coupling-scale parameter. Integer period counts are evaluated only on their discrete support. Table S2 gives the search bounds and the frozen reference point.

Table S2: Mixed discrete–continuous optimization space.

Variable	Meaning	Lower	Upper	Frozen
d_A (nm)	High-index thickness	145.00	178.00	161.50
d_B (nm)	Low-index thickness	240.00	295.00	267.24
d_{n} (nm)	Analyte thickness	480.00	720.00	600.00
d_{r} (nm)	Reference thickness	400.00	600.00	500.00
d_H (nm)	HMM-insert thickness	5.00	22.00	10.00
f	ITO filling fraction	0.42	0.58	0.50
N_{out}	Outer period count	3	7	4
N_{mid}	Central period count	1	4	2
ΔN	Mirror imbalance	0	4	0
c_κ	Coupling scale	0.80	1.20	1.00

The competing objectives minimize RI and temperature POD limits, poor conditioning, loss, and fabrication sensitivity while maximizing usable transmission, quality factor, angular operability, and specification yield. A design is feasible only if it retains exactly two identifiable modes in the declared windows, peak transmission of at least 0.30, loaded Q of at least 150, separation of at least 60 nm, scaled condition number no greater than 20, total thickness below 9500 nm, field-identity correlation of at least 0.80, and successful bounded nonlinear inversion. Hyperbolic coverage, spectral-boundary clearance, parasitic-peak limits, passivity, and manufacturable minimum thicknesses are also enforced.

The search proceeds in five stages. First, the 76 discrete configurations are enumerated and exactly screened. Second, a constrained radial-basis-function surrogate accelerates candidate acquisition for the expensive forward model [16]. Third, every retained design is recomputed using the exact dispersive optical, calibration, and measurement models. Fourth, feasible regions are refined with epsilon constraints, Pareto search, and derivative-free local search suitable for nonsmooth peak-tracking objectives [17, 18]; a reference-vector evolutionary calculation is used only as an independent diversity comparison [19]. Fifth, a predeclared selection rule freezes the existing reference unless a challenger improves RI POD-LOD by at least 2% without degrading yield by more than 0.02, loaded Q by more than 10%, or modal identity by more than 0.02. The selected design is described as exact-recomputed and Pareto feasible, not as a proven global optimum.

Algorithm S1 restates the same procedure as an ordered sequence, showing what each stage consumes and what it hands on. Three control points in it decide what the search is entitled to claim. The exact recomputation of the frozen reference is fail-closed: unless that reference itself passes every gate under the exact model, the margin, detection-limit, epsilon-constraint and Pareto stages do not start, so no challenger can be promoted against an unvalidated baseline. The surrogate appears only inside the acquisition loops; every design that reaches the non-dominated set is recomputed with the full dispersive optical, calibration, covariance and fabrication models, so no reported quantity originates in an interpolant. The reference-vector evolutionary search runs after the design has been frozen and is recorded as a diversity comparison, which is why it cannot alter the selection. A fourth point belongs with these three, and it concerns what the rule can be expected to do rather than what it forbids. The rule is asymmetric: it screens challengers on the primary objective and treats the remaining criteria only as non-degradation constraints. That is a defensible way to prevent a marginal detection-limit gain from being bought with yield, but it also means that if the primary objective happens to be flat over the feasible set the rule will retain the reference whatever the search finds. Section 3.8 of the article reports that this is what occurred, so the outcome should be read as an audit that the reference survives an exact Pareto recomputation and not as evidence that the search located an optimum.

Table S3 gives the numerical settings under which the algorithm was executed, together with the cost the reported run incurred. They are stated because the search is specific to this problem: the same stage sequence run with different budgets, tolerances or seeds is a different search, and none of these values follows from the physics. The budgets are per stage and per topology, so the total is not a number fixed in advance but the sum of what the promotion rules requested; here that came to 11 487 exact function evaluations. The retained archive is smaller than that count, because it keeps the 380 screening designs together with the best point returned by each stage rather than every trial point, and because repeated requests are served from the evaluation cache. It holds 433 distinct designs, of which 49 are feasible and 42 approximately non-dominated.

Fabrication uncertainty includes correlated common-mode and layer-specific thickness errors, HMM filling-fraction variation, roughness-equivalent mixed interfaces, refractive-index variation, analyte-calibration error, thermo-optic uncertainty, thermal-expansion uncertainty, angle jitter, and measurement error. The frozen distribution widths include relative local and global thickness standard deviations of 0.010 and 0.003, thickness correlation $\rho = 0.72$, HMM fill standard deviations of 0.015 (device level) and 0.010 (local), and relative thermo-optic and expansion standard deviations of 0.10 and 0.12. Each virtual device receives one internally consistent perturbation vector, and its complete spectrum and inversion are recomputed.

The final design is evaluated with $N = 1000$ independent Monte Carlo devices not used during

Algorithm S1 Hybrid fabrication-aware multiobjective optimization and exact validation.

Input: Discrete topology set $\mathcal{D}$; bounds $\mathcal{X}$ on the ten design variables; objective vector $\mathbf{F}$; normalized constraint vector $\mathbf{g}$; predeclared selection rule and uncertainty model

```
Seed every random stream from the declared master and optimizer seeds
for all d ∈ D do
    Draw the frozen reference, the box centre, and three Latin-hypercube points inside the bounds of d
    Evaluate them with the exact dispersive model; store F and g
end for
Search a narrow trust region around the frozen reference for a feasible start
Promote the twelve topologies with the smallest scaled violation
while fewer than three topologies are feasible and untried topologies remain do
    Run the constrained surrogate search on each promoted topology
    Promote six further topologies in order of scaled violation
end while
Recompute the frozen reference exactly; abort the remaining stages if it fails any gate
Maximize the normalized feasibility margin on the five best topologies
Minimize the full-covariance POD RI detection limit on the five best topologies
for all ϵ scenarios do
    Repeat the surrogate search under the tightened ϵ gates
end for
Refine by bound-constrained pattern search, then by local Pareto search
Pool the retained designs and take the feasible non-dominated set
Recompute every representative with the full calibration, covariance, POD–LOD and fabrication models; discard any that fails an exact gate
Apply the predeclared compromise rule: keep the frozen reference unless a challenger improves the RI POD–LOD by at least 2% without degrading yield, loaded Q or modal identity
Confirm the selection with an independent N = 1000 fabrication Monte Carlo and its 95% Wilson interval
Run the paired matched topological–trivial control protocols
Run the reference-vector evolutionary search as a diversity audit that cannot replace the selection
```

Output: Exact-recomputed Pareto-feasible reference design, uncertainty-bounded performance metrics, and matched-control results

optimization. If N_s devices pass every declared optical, modal-identity, conditioning, nonlinear-retrieval, and manufacturability gate, the yield estimate is

$$\widehat{p} = \frac{N_s}{N}. \tag{S21}$$

Its binomial interval is calculated with the Wilson score formula [20],

$$p_{\pm} = \frac{\widehat{p} + z^2/(2N) \pm z\sqrt{\widehat{p}(1-\widehat{p})/N + z^2/(4N^2)}}{1 + z^2/N}. \tag{S22}$$

The resulting numerical yield is reported in Section 3.8 of the article and is interpreted only under the declared uncertainty model.

Topology is assessed using paired topological–trivial controls. Protocol 1 matches the two resonance wavelengths, loaded quality factors, peak transmissions, and the total physical thickness. Protocol 2 uses a separately fitted trivial control with RI sensitivity as an explicit matching target; it is not simultaneously matched on every Protocol 1 observable, and its gate instead holds the loaded quality factors and peak transmissions near the target and requires the declared minimum mode separation. The same fabrication perturbation vector is applied to each member of a pair. With binary pass indicators I_j^{top} and I_j^{tri}, the paired yield difference is

$$\Delta\widehat{p} = \frac{1}{N}\sum_{j=1}^{N}\left(I_j^{\mathrm{top}} - I_j^{\mathrm{tri}}\right). \tag{S23}$$

Confidence intervals are obtained by bootstrapping paired device indices. This design tests an operating-point-dependent advantage while controlling sampled disorder severity; it does not assume universal topological protection.

Table S3: Numerical settings of the hybrid optimization procedure, and the cost of the reported run.

Setting	Value
Discrete configurations	76 unique $(N_{\text{out}}, N_{\text{mid}}, N_R)$ triples enumerated from $N_{\text{out}} \in [3, 7]$, $N_{\text{mid}} \in [1, 4]$ and mirror imbalance $\in [0, 4]$
Initial sampling	Five designs per configuration: the frozen reference, the box centre and three Latin-hypercube points; 380 exact evaluations in total
Continuous search space	Seven of the ten variables; the three period counts are handled by the enumeration
Surrogate	MATLAB `surrogateopt` radial-basis-function interpolant; 15 initial surrogate points ($2n + 1$ with $n = 7$); batch update interval equal to the eight parallel workers; one checkpoint per stage and topology
Constraint handling	22 gate residuals, each divided by its own gate value; feasible when the largest normalized residual is at most 10^{-4}
Objective scaling	$\log_{10}(\text{POD-LOD}/10^{-3}\ \text{RIU})$; $\text{FWHM}_{\max}/15\,\text{nm}$ plus a parasitic-peak penalty; $\log_{10}\kappa/\log_{10}20$; the angular and fabrication risks are already dimensionless
Feasibility stage	48 exact evaluations per topology; twelve promoted first, then batches of six until three topologies are feasible. All 76 were searched in the reported run
Baseline trust region	72 evaluations within ±4–18% of each variable range about the frozen reference
Margin stage	Five topologies, 48 evaluations each
Detection-limit stage	Five topologies, 78 evaluations each
ϵ scenarios	Seven: $\kappa \leq \{20, 18, 16, 20, 18, 16, 15\}$, common usable angle $\geq 15^\circ$, fabrication yield $\geq \{0.85, 0.90\}$, and FWHM $\leq \{12, 11, 10\}$ nm where imposed; two topologies and 48 evaluations per scenario
Pattern search	Five starting points, at most 90 evaluations, mesh tolerance 5×10^{-4}
Local Pareto search	Two topologies, at most 180 evaluations, at most 36 of the returned points kept, thinned by farthest-point sampling in normalized decision space
Diversity audit	Reference-vector search over 126 Das–Dennis vectors (four divisions, six objectives), 80 additional exact evaluations in five infill cycles of sixteen
Duplicate rejection	Archive entries are matched on the design vector rounded to eight decimals, with the three period counts rounded to integers first
Stopping	The per-stage evaluation budget; the pattern search also stops on its mesh tolerance
Monte Carlo sizes	120 samples per scenario inside the search gates, 300 for the post-optimization release gate, and an independent $N = 1000$ ensemble for the reported yield
Random seeds	Top-level Mersenne–Twister stream 42; optimizer base 271 828, with the topology-k screening seed $271\,828 + 7919k$ and the headline Monte Carlo at reserved offset 7777; parallel and per-analysis base 104 729, each site with its own reserved offset
Exact recomputation	Calibration, full covariance, POD–LOD and fabrication ensemble, for every retained representative
Cost of the reported run	11 487 exact function evaluations requested; 433 distinct designs retained, 49 feasible, 42 approximately non-dominated, 10 recomputed at full fidelity

S5 Independent Numerical Validation Models

S5.1 Computational Environment and Runtime

The production calculations were executed in MATLAB R2026a Update 3 [21] on a single desktop workstation: an Intel Core i9-14900KF processor (24 cores, 32 threads, 3.20 GHz base clock), 64 GB of DDR5 memory operating at 4800 MT/s, an NVIDIA GeForce RTX 5060 graphics processor with 8 GB, and Windows 11 Pro (build 26200, 64-bit). Monte Carlo sampling, the global search and the spectral sweeps are distributed over a pool of eight process workers. The graphics processor carries the Gaussian-process surrogate fits; the paged scattering-matrix kernel is benchmarked against its CPU implementation when the run starts and, on this machine, the CPU path is faster and is selected automatically, so no reported quantity depends on a graphics processor being present. A complete production run takes about an hour of wall-clock time on this workstation, varying with other load on the machine rather than with anything in the calculation, which is deterministic. Each run measures its own duration, from the first executable line of the script to the assembly of the verification package, and records it to the second; a single figure quoted here could not stay true of the run a reader is holding, so the measured value travels with the results instead. The timing excludes the external solves, which are separate steps: CST, FEniCSx, MEEP and MPB each run outside MATLAB and export the files the pipeline reads. For scale, the MEEP nominal spectrum takes about eight minutes on sixteen MPI ranks and the MPB audit about a minute.

The pipeline is a single MATLAB file, and it is written against four toolboxes: the Optimization Toolbox, for the bounded nonlinear least-squares solves behind the calibration surfaces; the Global Optimization Toolbox, for the surrogate-assisted, pattern-search and multiobjective stages of the hybrid search; the Statistics and Machine Learning Toolbox, for the Gaussian-process regression surrogate; and the Parallel Computing Toolbox, for the worker pool and the GPU arrays. Only the first three are required: the code detects the absence of the Parallel Computing Toolbox and continues serially on the CPU. The run opens by setting the top-level Mersenne–Twister stream to 42, which governs anything not seeded explicitly. Two declared bases then generate the reproducible streams: 271828 for the optimizer and everything derived from it, including the independent $N = 1000$ Monte Carlo confirmation, and 104729 for the parallel and per-analysis streams, among them the matched-control bootstrap. Every site mixes its base with a reserved offset and re-seeds before drawing, so no analysis inherits a stream another has already advanced.

S5.2 FDFD Model

An independent one-dimensional finite-difference frequency-domain model solves

$$\mathbf{A}(\omega)\mathbf{e} = \mathbf{b}, \tag{S24}$$

where $\mathbf{e}$ is the discretized electric field and $\mathbf{b}$ represents the incident source. The implementation independently samples the explicit material profile, applies absorbing boundary regions, and calculates reflected and transmitted flux from the solved fields; it does not call the layer-cascade routines. The total-field/scattered-field strategy follows established FDFD practice [22]. Spatial refinement continues to a 1 nm grid and is accepted only when both resonance centers and localization patterns converge. Numerical comparisons with the S-matrix model are reported in Section 3.12 of the article.

S5.3 CST Full-Wave Model

The explicit 36-layer stack is reconstructed in CST Studio Suite 2026 [23] as a three-dimensional frequency-domain finite-element model. The transverse boundaries are Floquet periodic, the ports are normal to the stack, and the lateral period is sufficiently small that only the zeroth Floquet order propagates. The CST model is built from the same layer order, thicknesses, complex dispersive material data, incident and exit media, analyte index, excitation side and spectral interval as the production model, so the two differ only in how they discretize the same structure.

Complex S_{11} and S_{21} are exported rather than comparing power spectra alone. Adaptive-mesh convergence is evaluated separately from cross-solver agreement. In addition to the nominal 5 nm/5 nm HMM, four explicit constituent corners are solved:

$$(d_{\mathrm{ITO}}, d_{\mathrm{TiO_2,H}}) \in \{(4.5, 4.5), (4.5, 5.5), (5.5, 4.5), (5.5, 5.5)\}\ \mathrm{nm}. \tag{S25}$$

Resonance wavelength, FWHM, loaded Q, peak transmission, complex scattering, and mesh convergence are evaluated using the same post-processing definitions as the production model. The resulting discrepancies belong in Section 3.12 of the article, not in the method definition.

S5.4 FDTD Model

A third electromagnetic solver time-steps the same explicit 36-layer stack in MEEP [24], on a uniform Yee grid at normal incidence, where the two polarizations are degenerate. Its independence from the other two lies in the formulation rather than in the code: the material dispersion is carried by auxiliary differential equations advanced alongside the fields, rather than by a permittivity evaluated once per wavelength. The stack, the complex indices, the incident and exit media and the spectral grid are read from the same exported definition the CST model is built from, so the two full-wave checks share their inputs and differ only in how they discretize the problem.

The ITO Drude permittivity is one of the solver's native susceptibilities and transfers without fitting. The three dielectrics are represented by non-negative Lorentzian oscillators with poles

outside the spectral support of the excitation, fitted to the same tabulated indices the production model evaluates, and their small extinction is carried by a conductivity matched at the reference wavelength. Because a dispersion fit that is accurate on the comparison window can still be unphysical outside it, the fit is constrained and its residual is gated separately from the solver comparison, as is a slab test of the phase convention and reference planes whose answer is known in closed form. Both belong to the model rather than to its result, and both are described in S8. Reference planes sit on the two stack faces, so the exported S_{11} and S_{21} are directly the transfer-matrix r and t. Grid refinement, the nominal design and the same four constituent corners of Equation (S25) are all solved; the discrepancies are reported in Section 3.12 of the article.

S5.5 Plane-Wave Eigensolver Model

The topological interpretation is the most delicate claim in this work, and it rests on a bulk band structure, on inversion parities at the two high-symmetry points, and on a Zak phase built from them, all computed by the plane-wave expansion of Section S3.2. Those quantities are recomputed independently in MPB [12], a block-iterative preconditioned eigensolver, for the same two half-stack unit cells at the same non-dispersive reference indices.

The audit adds one thing the production calculation does not have: a second route to the Zak phase. Alongside the parity product it evaluates a discrete Berry phase around the Brillouin zone,

$$\zeta_n = -\arg\prod_j \left\langle u_{n,k_j}\middle|\varepsilon\middle|u_{n,k_{j+1}}\right\rangle, \tag{S26}$$

closed through the periodic gauge $u_{n,k+G} = \mathrm{e}^{-\mathrm{i}Gx}u_{n,k}$. Equation (S26) never refers to a parity, so agreement between the two routes is a genuine check rather than a restatement of the same argument. Only the difference between the two half-stacks is reported as a result, because an individual Zak phase depends on where the unit-cell origin is placed while the difference does not, and the difference is what bulk–boundary correspondence uses. S8 records the three conventions the calculation had to measure rather than assume.

S5.6 Finite-Element Transport–Reaction Model

A two-dimensional axial–height model is implemented in FEniCSx [25], an open-source finite-element framework, driven from Python. The channel has height 50 µm, width 500 µm, and length 10 mm; the width enters the prescribed mean velocity through the nominal flow rate of $10\,\mu\mathrm{L\,min^{-1}}$. The dissolved target concentration satisfies

$$\frac{\partial c}{\partial t} + \mathbf{u}\cdot\nabla c = D\nabla^2 c, \tag{S27}$$

with $D = 6.0\times10^{-11}\ \mathrm{m^2\,s^{-1}}$. A localized lower-wall patch follows reversible Langmuir kinetics [26],

$$\frac{\mathrm{d}\Gamma}{\mathrm{d}t} = k_a c_s(\Gamma_{\max}-\Gamma) - k_d\Gamma, \tag{S28}$$

coupled through

$$-\mathbf{n}\cdot D\nabla c = \frac{\mathrm{d}\Gamma}{\mathrm{d}t}. \tag{S29}$$

The declared inputs are $k_a = 1.0\times10^5\ \mathrm{M^{-1}\,s^{-1}}$, $K_d = 5$ nM, $\Gamma_{\max} = 2.0\times10^{-8}\ \mathrm{mol\,m^{-2}}$, a 10 nM inlet concentration, 3600 s association, and 2400 s wash.

The flow is solved first as a stationary Stokes problem with Taylor–Hood $P2+P1$ elements; because the quadratic velocity space represents the parabolic channel profile exactly, the discrete solution reproduces the analytical parallel-plate pressure drop and wall shear to round-off. Transport is then advanced with backward Euler on a fixed flow field. The cell Péclet number reaches $\sim 5.6\times10^3$, so the advective term is SUPG-stabilised; without stabilisation a Galerkin discretisation oscillates. The surface equation carries no diffusive term, so the coverage obeys an independent ordinary differential equation at every patch node and is integrated pointwise with backward Euler.

The FEniCSx outputs are compared with an independently implemented reduced-order transport model at the observable level. The predeclared gates cover mean velocity, pressure drop, wall shear, residence time, surface concentration, surface coverage, self-referenced wavelength shift, association time, and the transport-map pressure drop. Four mesh levels and four time steps are used to demonstrate numerical convergence; the time-step study varies the integration step itself, not the interval at which results are written. Parity and Bland–Altman residual plots are used to show both the parity relationship and absolute disagreement [27]. The model is an idealized numerical consistency test; it does not establish molecular selectivity, nonspecific-adsorption control, receptor chemistry, or biological detection.

S5.7 Finite-Element 3D Hydrodynamic Model

A separate stationary three-dimensional model tests the finite-width correction omitted by the two-dimensional parallel-plate approximation. It solves

$$\nabla \cdot \mathbf{u} = 0, \tag{S30}$$

$$\rho(\mathbf{u} \cdot \nabla)\mathbf{u} = -\nabla p + \mu \nabla^2 \mathbf{u}, \tag{S31}$$

with no slip on all channel walls. Four aspect ratios $H/W = 0.10, 0.25, 0.50$, and 1.00 are considered. Taylor–Hood $P2 + P1$ elements are used on a structured tetrahedral mesh graded towards the walls of the cross-section, which is where the shear that sets the pressure gradient lives; the axial direction is flow-invariant and needs only a few layers. Pressure is sampled on the centreline between the interior, fully developed cross-sections at $x = 0.2L$ and $0.8L$ to remove entrance bias. The computed pressure drop is compared with the analytical rectangular-duct series and the reduced two-dimensional correction. This model validates finite-width fluid mechanics only; it is not a full three-dimensional reactive biosensor simulation.

The numerical evidence hierarchy is therefore: stabilized S-matrix, explicit-bilayer recomputation, independent FDFD, CST full-wave scattering, FEniCSx two-dimensional transport–reaction, FEniCSx three-dimensional hydrodynamics, and statistical Monte Carlo/control comparisons. Consistency across this hierarchy reduces implementation-specific risk but does not replace fabrication, film characterization, optical measurement, device calibration, or analyte-selectivity experiments.

S6 Complete Model Parameters

The frozen geometry is tabulated in Table 1 of the article and the thermo-optic and thermal-expansion coefficients in Table S1, both in Section 2 of the article. This appendix supplies the inputs those two tables do not carry, repeating the thermal coefficients alongside them for convenience: the refractive indices together with the dispersion models that produce them, the extinction coefficients, the complete ITO Drude set with its derived carrier parameters, and the per-film hyperbolic-window data. With those tables the model is then fully specified.

Table S4: Material parameters. n_0 and k are quoted at T_0 and at the 1.55 µm reference wavelength; $\alpha = \partial n/\partial T$ and β is the linear thermal-expansion coefficient. Extinction coefficients carry their own relative temperature coefficient.

**Medium**	$\boldsymbol{n_0}$	$\boldsymbol{\alpha}$ **(K^{-1})**	$\boldsymbol{k}$	$\boldsymbol{\beta}$ **(K^{-1})**
High-index layer A (TiO_2)	2.3999	8.00×10^{-5}	2.00×10^{-5}	8.00×10^{-6}
Low-index layer B (fused SiO_2)	1.4440	1.00×10^{-5}	1.00×10^{-7}	5.50×10^{-7}
Temperature-reference coating	1.4100	-4.50×10^{-4}	1.00×10^{-5}	1.00×10^{-4}
Analyte domain	1.3300	—	—	5.00×10^{-6}
Nanolaminate block	—	—	—	5.00×10^{-6}

The analyte entry carries the coefficient of the channel wall, not of the liquid. It is the analyte *layer thickness* that enters the transfer matrix, and the thickness of a buried channel is fixed by

the solid that bounds it: a liquid connected to a reservoir expands into the reservoir rather than displacing the mirrors on either side. The channel here is bounded by the nanolaminate and by the U_2 mirror, so it takes the nanolaminate value, which lies between the TiO_2 and SiO_2 coefficients of the mirror itself. Section 3.5 of the article reports what the alternative treatments give.

All three dielectrics are dispersive, and the indices in Table S4 are the values their dispersion models give at the reference wavelength rather than fixed constants. Layer A uses a Cauchy form $n = A + B/\lambda^2$ with $A = 2.30$ and $B = 0.240$ μm^2; layer B uses the three-term fused-silica Sellmeier model; and the reference coating uses $n = n_0 + B(\lambda^{-2} - \lambda_{\text{ref}}^{-2})$ with $B = 0.010$ μm^2 and $\lambda_{\text{ref}} = 1.55$ μm, so its index equals n_0 exactly at that wavelength. The relative temperature coefficients of the extinction coefficients are 2.00×10^{-3}, 5.00×10^{-4} and 3.00×10^{-3} K^{-1} for layer A, layer B and the reference coating.

Table S5: ITO Drude parameters of Equation (S5). The carrier density and mobility are not independent inputs: they follow from ω_p, γ and m^* and are listed because the process-window analysis is parameterised in those terms.

Symbol	Quantity	Value
ε_∞	High-frequency permittivity	3.9
ω_p	Plasma frequency	2.65×10^{15} rad s^{-1}
γ	Damping rate	1.20×10^{14} s^{-1}
m^*/m_e	Relative effective carrier mass	0.35
N	Derived carrier density	7.72×10^{20} cm^{-3}
μ	Derived carrier mobility	41.9 cm^{2}V^{-1}s^{-1}
—	Relative temperature coefficient of ω_p	-1.00×10^{-4} K^{-1}
—	Relative temperature coefficient of γ	1.00×10^{-3} K^{-1}

Table S6 reports the per-film hyperbolic-window classification referred to in Section 3.10 of the article, relevant to the historical optimization constraint and to oblique-TM operation. This classification does not affect any headline normal-incidence sensing metric. If oblique-TM operation is intended, however, the hyperbolic onset must lie below the shorter resonance; one of the three measured films fails that classification.

Table S6: Hyperbolic-window coverage of the Drude baseline and the three ellipsometric ITO datasets. This is a classification and oblique-TM criterion, not a normal-incidence performance gate. Window edges marked with a dagger are limited by the evaluation grid or by the measured support and are therefore lower bounds, not measured edges.

Dataset	Onset (nm)	End (nm)	Re($\varepsilon_\perp$) at λ_1	Re($\varepsilon_\perp$) at λ_2	Both hyperbolic
Drude model baseline	1413.8	1688.7†	−1.28	−3.58	yes
ITO Minenkov2024 on glass	1263.9	1688.7†	−4.61	−9.35	yes
ITO Minenkov2024 wafer bottom	1102.0	1683.7	−11.20	−7.09	yes
ITO Minenkov2024 wafer top	1574.7	1688.7†	+0.49	−0.76	no

S7 Numerical Acceptance Criteria

Every "pass", "yield" and "converged" reported in this paper is decided against a threshold fixed before the calculation ran. Table S7 collects them. The MEEP and MPB groups were added the same way: their thresholds were written down before any spectrum or band structure had been compared, and the electromagnetic ones are the values already declared for CST rather than a second, looser standard invented alongside a second set of results. The Specification group contains the three headline optical thresholds; fabrication-yield pass status is determined by the complete set of applicable pass/fail gates in the table, which is why modal survival alone

overestimates yield, while the detection-limit rows give reporting targets only and are not a Monte Carlo pass/fail gate.

Table S7: Numerical acceptance criteria, grouped by the decision each one controls. *The detection-limit row states the reporting targets at which the POD limits are evaluated; it is not a pass/fail gate, and no Monte Carlo gate constrains the detection limit.

**Group**	**Criterion**	**Threshold**
Specification	Minimum peak transmittance	0.30
	Minimum loaded Q	150
	Maximum scaled condition number	20
Modal survival	Minimum peak transmittance	1.00×10^{-5}
	Minimum loaded Q	5
	Minimum mode separation	8 nm
	Maximum admissible resonance jump	500 nm
Spectral fit	Maximum fitted-centre shift	1.5 nm
	Outlier rejection	4.5 MAD
	Maximum fit RMSE	0.02
	Minimum valid-fit fraction	0.95
Nonlinear retrieval	Maximum RI RMSE	1.00×10^{-3} RIU
	Maximum temperature RMSE	5.0 °C
	Minimum inversion success fraction	0.95
Detection limit*	Target detection probability	0.95
	False-alarm probability	0.01
Manufacturability	Resonance-preservation tolerance	1.5 nm
	Relative Q and sensitivity tolerance	0.15
	Assumed constituent design floor	5.0 nm
	Worst-case interface roughness (RMS)	3.0 nm
CST agreement	Maximum power-spectrum RMSE	0.035
	Maximum complex-S RMSE	0.15
	Maximum resonance-wavelength error	2.0 nm
	Maximum relative loaded-Q error	0.10
	Maximum relative FWHM error	0.10
	Maximum peak-transmittance error	0.03
CST mesh convergence	Maximum resonance-wavelength change	0.60 nm
	Maximum relative loaded-Q change	0.08
	Maximum relative FWHM change	0.08
	Maximum peak-transmittance change	0.03
FEniCSx agreement	Maximum summary relative error	20%
	Maximum surface-kinetics NRMSE	15%
	Maximum transport-map NRMSE	25%
	Maximum transport-map MAPE	20%
	Plateau-coverage convergence tolerance	2%
MEEP agreement	Maximum power-spectrum RMSE	0.035
	Maximum complex-S RMSE	0.15
	Maximum resonance-wavelength error	2.0 nm
	Maximum relative loaded-Q error	0.10
	Maximum relative FWHM error	0.10
	Maximum peak-transmittance error	0.03
	Minimum field-profile correlation	0.98
MEEP model transfer	Maximum refractive-index transfer error	1.00×10^{-3}
	Maximum extinction transfer error	1.00×10^{-3}
	Maximum slab convention self-test error	5.00×10^{-3}
MEEP grid convergence	Maximum resonance-wavelength change	0.60 nm
	Maximum relative loaded-Q change	0.08
MPB agreement	Maximum band-frequency error, band-scaled	1.00×10^{-3}
	Minimum inversion-parity magnitude	0.95
	Band inversion on every isolated band	must agree
	Maximum Berry-loop Zak deviation from π	0.05 rad
	Maximum gap-edge change on refinement	1.00×10^{-4}

S8 Solver and Convergence Details

S8.1 Scalar Redheffer Star Product

The stabilized scattering-matrix cascade of Section S2.1 combines the sub-matrices S^A and S^B of two adjacent blocks through the Redheffer star product. With the common denominator

$$D = 1 - S^A_{22} S^B_{11}, \tag{S32}$$

the four scalar coefficients of the combined block are

$$S^{AB}_{11} = S^A_{11} + \frac{S^A_{12} S^B_{11} S^A_{21}}{D}, \qquad S^{AB}_{12} = \frac{S^A_{12} S^B_{12}}{D}, \tag{S33}$$

$$S^{AB}_{21} = \frac{S^B_{21} S^A_{21}}{D}, \qquad S^{AB}_{22} = S^B_{22} + \frac{S^B_{21} S^A_{22} S^B_{12}}{D}. \tag{S34}$$

The same expressions are used for TE and TM; the polarizations differ only through the layer wave numbers and interface impedances that enter the individual S^A and S^B, not through the cascade itself. Because D never contains a growing exponential of a passive evanescent layer, the cascade stays bounded where a direct transfer-matrix product overflows.

S8.2 FDFD Grid Convergence

The one-dimensional FDFD solver discretizes the spatial Maxwell operator directly instead of rearranging the same layer matrices, so agreement with the production model is not guaranteed by a shared implementation. Both modes converged at a 1 nm grid step, giving resonance centres at 1516.79 nm and 1651.20 nm. The loaded Q is not quoted here because the grid study applies the FDFD solver's own peak post-processing; the cross-solver Q comparison reported in Section 3.12 of the article uses common post-processing for both solvers and is the meaningful one.

S8.3 CST Full-Wave Model

The finite multilayer is reproduced in CST with all 36 physical layers retained rather than homogenized, using waveguide or Floquet ports matched to the illumination configuration. Convergence is established by refining the mesh until the resonance observables stop moving by more than the thresholds of Table S7, and agreement with the production model is then evaluated on the resonance wavelength, the loaded Q, the FWHM and the peak transmittance separately, so that a coincidental match in one observable cannot carry the others. The same comparison is repeated at the four ± 0.5 nm ITO/TiO_2 thickness corners.

S8.4 FDTD Model

The same explicit 36-layer stack is time-stepped in MEEP [24] on a uniform Yee grid at normal incidence, where the two polarizations are degenerate. The dispersion enters as auxiliary differential equations rather than as a permittivity evaluated once per wavelength, which is what makes this an independent check rather than a re-implementation of the production model. Reference planes are placed on the two stack faces and the intervening vacuum propagation is removed analytically, so the exported S_{11} and S_{21} are directly the transfer-matrix r and t. The production grid is 4000 pixels per micrometre, a 0.25 nm step, and the comparison uses the same 1460–1700 nm grid at 0.25 nm as the CST export.

Two properties of the model are checked before any comparison is made, because either could otherwise be mistaken for a solver disagreement. The first is the material transfer. The ITO Drude permittivity is one of the solver's native susceptibilities and transfers exactly; the three dielectrics are fitted with Lorentzian oscillators whose strengths are constrained to be non-negative and whose instantaneous permittivity is held at or above that of vacuum, with all poles kept clear of the spectral support of the pulse. Those constraints are not cosmetic: an unconstrained fit reproduces the 240 nm comparison window as the near-cancellation of two very large opposite-sign oscillators, which is accurate exactly where it is tested, implies an instantaneous permittivity

wrong by five orders of magnitude, and is unstable in the time domain. The constrained fit reproduces the tabulated indices the production solver evaluates to better than 1.4×10^{-5} in n, corresponding to a resonance shift near 0.01 nm, and it recovers the physical infrared Sellmeier pole of fused silica at 9870 nm with strength 0.892 against the model's 9896 nm and 0.897. The second is the phase convention. A single lossless slab, whose complex transmission and reflection follow in closed form from the same characteristic matrix the production solver uses, is run through the identical monitor, normalization and de-embedding arithmetic; the worst complex error is 1.0×10^{-5}. No phasor or port correction is therefore applied to the stack comparison, unlike the CST comparison, where the opposite time-harmonic convention makes one necessary.

S8.5 Plane-Wave Eigensolver Audit

The bulk band structure, the inversion parities and the Zak phase of the two half-stack unit cells are recomputed in MPB [12], a block-iterative preconditioned eigensolver, using the non-dispersive reference indices the topological analysis uses. Three details are measured rather than assumed, because each one changes the answer silently.

The operator that implements $x \to -x$ on the solver's grid is chosen as the index reversal that leaves the sampled dielectric function unchanged, which both cells admit by construction; the residual asymmetry is zero. The position variable used to strip the Bloch factor in the Berry loop is taken from the geometry: measuring it from the cell corner rather than the cell centre shifts the origin by half a period, leaves every band and every parity untouched, and adds exactly π to the Berry-loop Zak phase. At the zone centre the lowest band is the zero-frequency uniform mode, for which the solver returns a null field, so the parities and the Berry loop are anchored a short distance away; over offsets from 0.05 to 0.001 of the zone the parity sign stays $+1$, the parity magnitude rises to 0.99999 and the field non-uniformity falls to 4×10^{-7}, which is the limit being claimed.

What the audit gates is the band inversion and the Zak-phase difference, not the individual parity signs. The two codes agree on every zone-centre parity and disagree on every zone-edge parity, uniformly, for the reason given in Section S11.2 of the article: a half-period displacement of the Bloch phase reference flips all of them together. Gating the raw signs would test that choice rather than the crystal.

Only isolated bands are gated. The two layers of this design have almost equal optical thickness at the non-dispersive design indices the band-structure cell is built from, $n_A = 2.40$ and $n_B = 1.45$ rather than the dispersive values tabulated above: $n_A d_A = 387.600$ nm against $n_B d_B = 387.498$ nm, which closes the gap between the second and third bands at the zone centre: the measured separation there is 3.6×10^{-5} in normalized frequency against 8.8×10^{-2} for the first band. A Zak phase is defined only for a band separated from its neighbours throughout the zone, so the second band does not have one here and none is reported for it. The first band carries the invariant the bulk–boundary argument uses.

S8.6 Finite-Element Transport–Reaction and Hydrodynamic Models

Mesh refinement spanned 1200 to 28,800 elements and temporal refinement 60 to 7.5 s, the latter varying the backward-Euler integration step itself rather than the interval at which results are written. Between the two finest mesh levels the plateau coverage changed by 0.00084% and the association t_{90} by 0.030%; between the two finest time steps the changes were 0.021% and 0.51%. The temporal sequence 1717.2 → 1684.0 → 1667.2 → 1658.8 s halves its increment at each refinement, which is the first-order behaviour backward Euler must show, and Richardson extrapolation places the step-independent value near 1650 s. Both are far inside the 2% and 8% tolerances, so the remaining discrepancy against the reduced-order description is a model-reduction difference and not an unresolved discretization error.

S9 Narrative Summary Figures

These three composite panels restate results the article reports in their own right. Each duplicates content shown at full size elsewhere, so a reader following the argument does not need them twice; they are kept here because they are useful as a single-view summary.

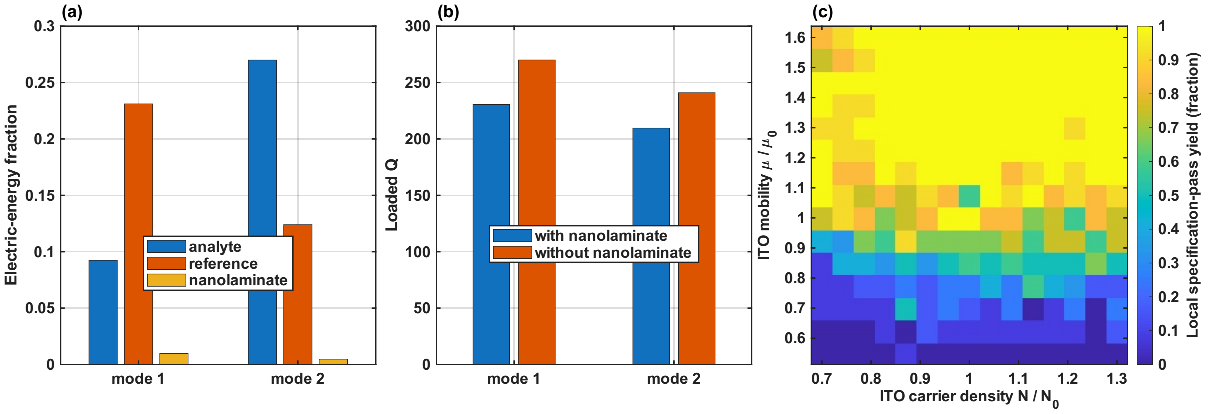

Figure S1: Backbone narrative panel: what the ITO/TiO_2 nanolaminate changes and what it costs. The composite panel shows (**a**) the analyte, reference, and nanolaminate electric-energy fractions of the two coupled interface modes, quantifying the field-redistribution effect of the nanolaminate; (**b**) the loaded-Q values with and without the nanolaminate, which show that the anisotropic block is not free: loading it lowers the loaded Q of modes 1 and 2 by 14.6% and 13.0% respectively, so the overlap control of panel (**a**) is bought at a measurable linewidth cost; and (**c**) the local specification-pass yield as a function of the normalized ITO carrier density N/N_0 and carrier mobility μ/μ_0, where N_0 and μ_0 denote the baseline Drude parameters. The underlying hyperbolic-window tensor components and the full explicit-versus-EMT spectrum are given in Figure 6 of the article and Figure 7 of the article.

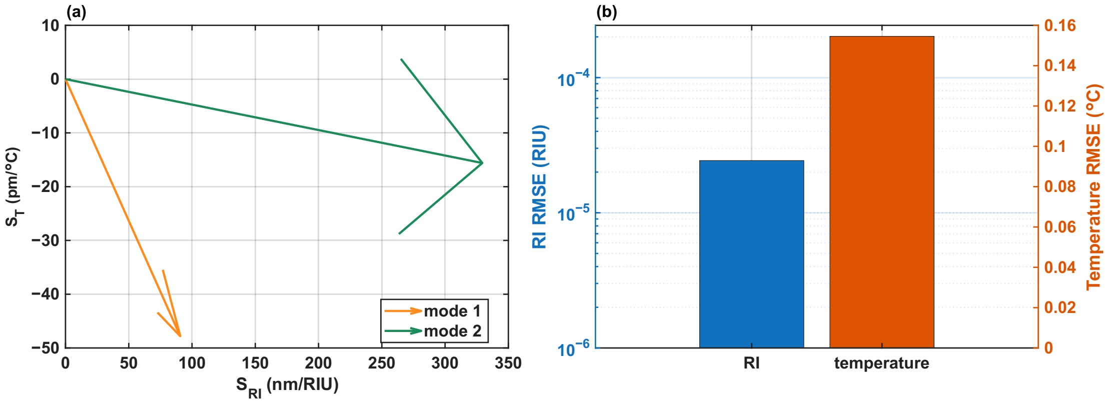

Figure S2: Backbone narrative panel: can RI and temperature be separated? The composite panel visualizes the nonlinear dual-parameter self-referencing, combining (**a**) the per-mode response vectors in RI–temperature space, showing that the two modes track along clearly nonparallel directions; and (**b**) the held-out validation RMSEs of the bounded nonlinear inversion, 2.43×10^{-5} RIU for refractive index and 0.155 °C for temperature. The per-parameter parity plots are given in Figure S10 and the full error-budget decomposition in Figure 11 of the article.

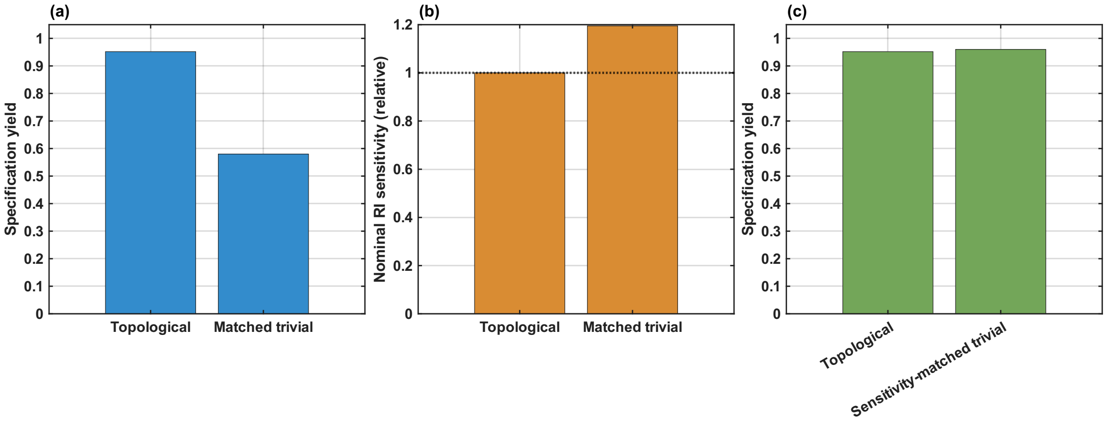

Figure S3: Backbone narrative panel: matched-control assessment of the topological contribution. The composite panel juxtaposes the paired matched-control experiments used in Section 3.9 of the article: (**a**) under Protocol 1, which matches resonance wavelength, loaded Q, peak transmission and total physical thickness, the topological design attains the higher specification yield, with a paired difference $\Delta Y = 0.372$ (95% bootstrap CI [0.31, 0.43]) that is below the median of the 14 admissible controls enumerated in Table S9; (**b**) topology is not a sensitivity gain, since the spectrally matched trivial control is in fact approximately 1.19× more RI-sensitive at the nominal operating point; and (**c**) in the separately fitted RI-sensitivity-matched Protocol 2 control, the yield difference is no longer statistically resolved and its sign reverses, with the paired data excluding any topological advantage above 2.4 percentage points. Full paired-difference bootstrap distributions and the sensitivity-matching audit are reported in the text and in Figure 15 of the article. Protocol 1 shows an apparent specification-yield advantage when the spectral observables are matched, whereas Protocol 2 removes the statistically resolved advantage when RI sensitivity is matched separately. The extended jointly matched control reported in Table S14 simultaneously satisfies the declared spectral, thickness and RI-sensitivity criteria and reverses the yield difference ($\Delta Y = -0.044$, 95% bootstrap CI $[-0.072, -0.020]$). These comparisons therefore do not isolate a robustness advantage attributable to topology; the Protocol 1 result is explained by differences in conditioning within that control family.

S10 Angular and Polarization Behaviour

Section 3.11 of the article states the operating consequence of the angular dependence in one paragraph. The angle-resolved maps behind it (Figure S4), the drift, polarization and alignment figures, and the control that assigns the behaviour to the interface geometry rather than to the nanolaminate (Figure S5) are given here; the three paragraphs and the first figure below were relocated from the article.

The device is a planar stack read out in transmission, so its resonances depend on the illumination angle, and a dual-parameter readout is usable only if that dependence is small or controlled. Mapping the transmittance over 0–15° for both polarizations (Figure S4) shows that the inverse problem survives: both modes stay continuously trackable, the minimum separation holds at 130.3 nm for TE and 132.1 nm for TM, the RI sensitivities never fall below 99.01 and 325.91 nm/RIU, and the scaled condition number degrades only from 8.20 at normal incidence to 9.09 for TE and 9.22 for TM. The 15° limit is the full range evaluated rather than a measured breakdown angle, so the usable angle is a lower bound.

The drift itself sets the operating requirement. The resonances move by up to 17.43 and 21.51 nm in TE and 20.29 and 22.56 nm in TM over that range, with RMS drifts near 8–11 nm, against 0.33 nm for a 10^{-3} RIU index change on mode 2, so angle is a first-order systematic error and the readout must either fix the incidence angle or measure it; these numbers are a specification for the collimation optics, not evidence of angular immunity. Polarization is milder within a fixed geometry: the maximum TE/TM splitting is 2.860 nm, TE retains at least 99.99% of the normal-incidence loaded Q of mode 1 against 91.1% for TM, TM preserves peak transmittance better (99.1% against 96.1%), and both satisfy the declared thresholds over the whole range. A matched comparison assigns this behaviour to the interface geometry rather than to the nanolaminate. Removing the layer changes the TE RMS drift of mode 1 only from 8.22 to 8.28 nm and leaves the usable angle unchanged, two of the remaining differences are below 1%, and the third runs

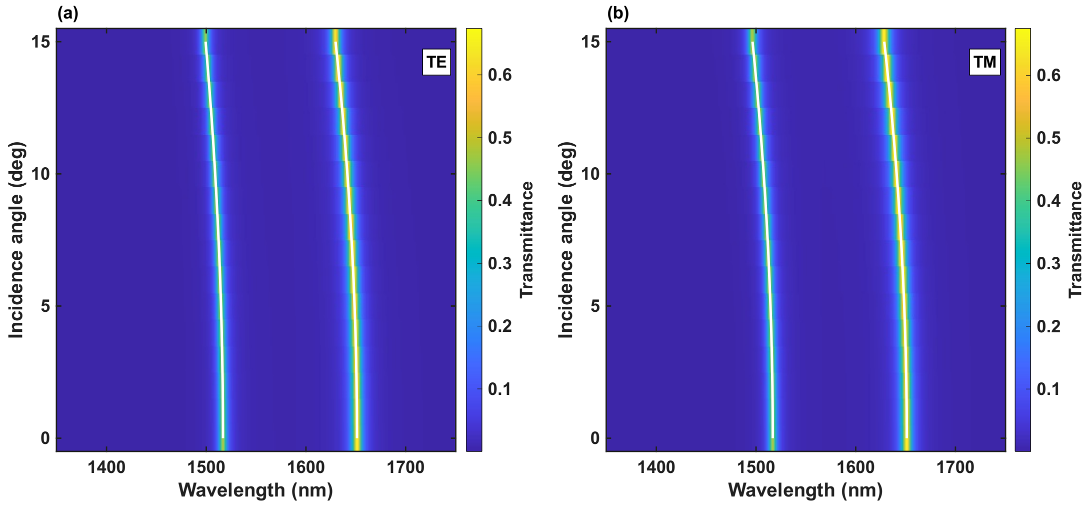

Figure S4: Angle-resolved transmittance of the complete device for (**a**) TE and (**b**) TM illumination, over 1350–1750 nm and 0–15° of incidence in 1° steps. Colour encodes transmittance, the overlaid markers are the tracked positions of the two coupled interface resonances at each angle, and the boxed tag identifies the polarization. Both modes are continuously trackable over the whole range in both polarizations, with a minimum separation of 130.3 nm for TE and 132.1 nm for TM and a maximum TE/TM splitting of 2.860 nm. The resonances nonetheless drift by up to 21.5 nm in TE and 22.6 nm in TM across the range, comparable to the shift a 6.5×10^{-2} RIU index change would produce on mode 2, so the incidence angle is a controlled operating parameter and not a negligible one.

against the layer, the worst-case scaled condition number over the sweep being 9.41 with it against 8.44 without, an 11% degradation with both far below the threshold of 20. None is a resolvable effect, so presenting the layer as an angular benefit would overstate what it does, which is the verdict the audit of Section 3.3 of the article reaches on every other metric.

Angle enters the error budget a second time, through the residual jitter about the nominal setting rather than through a known angle. Because the transmittance of a planar stack is even in θ, $\mathrm{d}\lambda/\mathrm{d}\theta$ vanishes at normal incidence, and central differences at static misalignments of 0.1, 0.25, 0.5, 1 and 2° give $|\mathrm{d}\lambda/\mathrm{d}\theta|$ of 0.020, 0.040–0.059, 0.099–0.100, 0.180–0.219 and 0.380–0.400 nm/deg for the two modes. A 0.02° jitter then contributes 4.0×10^{-4} nm at 0.1° and 4.4×10^{-3} nm at 1°, against the 1.0×10^{-3} nm laser-reference term of Section 3.6 of the article, so the alignment term stays below the laser reference only while the static misalignment is under about 0.25°. Section 2.5 of the article adopts the more conservative ±0.1° as the operating specification. This is the least demanding requirement listed here, because the penalty falls quadratically as the nominal angle approaches zero instead of needing active correction.

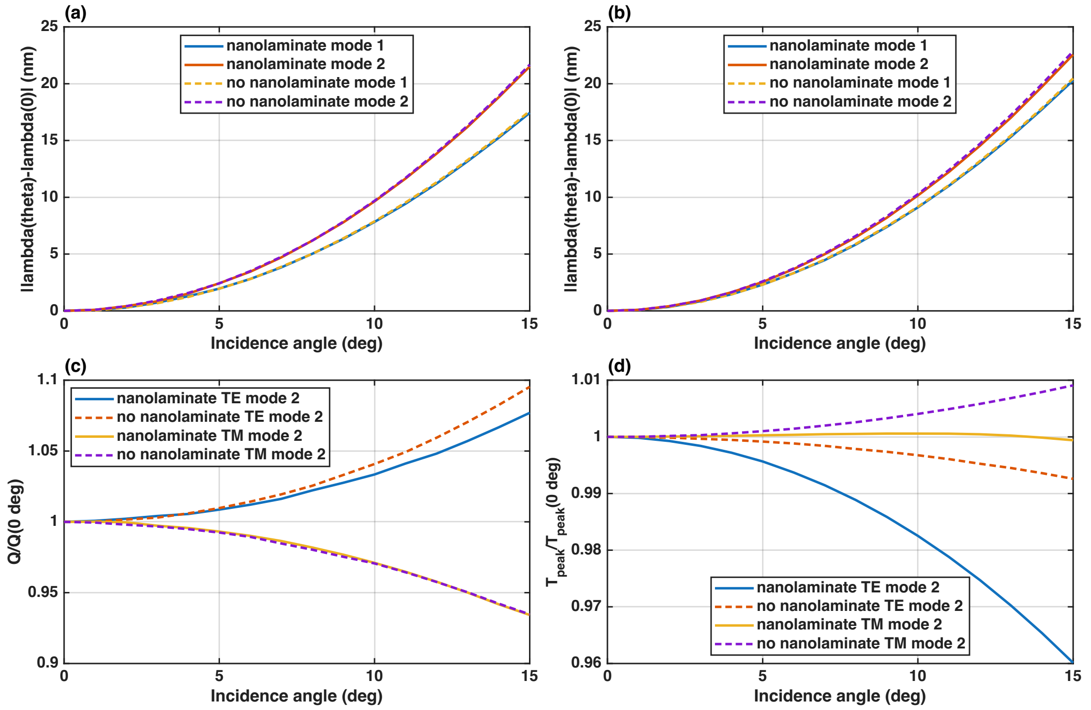

Figure S5: The matched control that assigns the angular behaviour. The sweep of Figure S4 is repeated with and without the nanolaminate: (**a**) TE angular wavelength drift, (**b**) TM angular wavelength drift, (**c**) loaded-Q retention, and (**d**) transmission retention. Removing the nanolaminate changes the TE drift of mode 1 only from 8.22 to 8.28 nm and leaves the usable angle unchanged; the remaining differences in the drift and retention metrics plotted here run in both directions and none exceeds 1%, while the worst-case scaled condition number over the sweep is 9.41 with the nanolaminate against 8.44 without it, an 11% conditioning penalty carried by the layer. No angular benefit attributable to the nanolaminate is resolved, consistent with the deletion audit of the article, in which every measured metric favours the stack without it.

S11 Independent Numerical Validation in Detail

The article summarises the cross-solver agreement in one table (Table 5) and one paragraph (Section 3.12). The spectral overlay of the four electromagnetic formulations (Figure S6) and the two paragraphs after it were relocated from the article; the full audit then follows: the grid-convergence history of each solver, the per-metric error breakdown, and the two comparisons that test something other than a resonance wavelength, namely the modal field distribution and the transport–reaction model.

Two of these tests do more than confirm a peak position. The plane-wave eigensolver reproduces the band inversion and the π Zak-phase difference between the two half-crystals, and a discrete Berry phase evaluated on its eigenvectors, which never refers to a parity, returns 3.1393 rad, 0.07% from π, so the topological premise is not an artifact of the parity bookkeeping written for this study. The time-domain solve independently places 0.232 of the mode 1 energy in the reference coating against 0.091 in the analyte, and 0.272 of mode 2 in the analyte against 0.125 in the reference, with profile correlations of 0.99999 against the production reconstruction; that asymmetry and its reversal between the modes is the physical content of the dual-parameter separation.

The largest disagreement in the set is the 1.96% CST error in the mode 1 peak transmittance, an external-coupling quantity and the one most sensitive to the port and mesh treatment; every wavelength error is below 0.02% and every linewidth error below 0.5% for the two frequency-domain solvers. None of this is experimental evidence: agreement across five formulations reduces the chance that the resonances are an artifact of one code path and says nothing about deposition defects, interface contamination, substrate effects, instrument drift or packaging.

The independent FDFD solver reproduced the two resonance centers to the reported table precision. Independently sampled FDFD markers lie on the stabilized S-matrix curves through

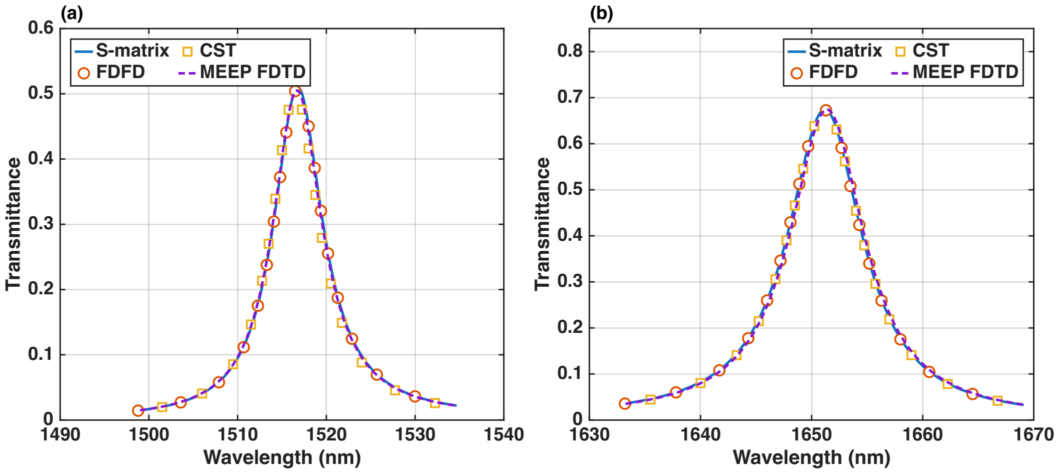

Figure S6: The Redheffer S-matrix reference and three independent finite-device electromagnetic discretizations on one pair of axes. (**a**,**b**) Stabilized S-matrix spectra for modes 1 and 2, with the 1D FDFD samples, the CST finite-element points and the MEEP FDTD curve overlaid. The FDFD samples lie on the S-matrix curves through the rising flank, the maximum and the falling flank, so the agreement covers linewidth and height and not peak position alone: the peak-transmittance differences are 0.00171 and 0.00125 and the loaded-Q differences 0.42% and 0.31%. For legibility the FDFD and CST results are drawn as a sparse set of markers rather than as their full series, which carry 721 and 144 samples across the plotted span and would otherwise form solid bands hiding the S-matrix curve; the markers are placed at equal intervals along the curve, so that they fall on the flanks and the maximum rather than on the flat wings, and the two sets are offset from each other so they interleave. Every error quoted here and in Table 5 of the article is computed from the complete series, not from the drawn subset.

the rising flank, maximum, and falling flank of both resonances, so agreement is obtained for peak center, linewidth, and height rather than for wavelength alone. With common peak post-processing, the peak-transmittance differences were 0.00171 and 0.00125, while the loaded-Q differences were approximately 0.42% and 0.31%.

The grid audit behind that agreement is given in Table S8 rather than asserted, because a second-order finite-difference scheme cannot reproduce a transfer-matrix result exactly and reporting it as though it had would apply a different standard than the time-domain comparison below, whose convergence history is quoted with its non-monotone behaviour intact. Second-order differencing carries a relative wavenumber error of order $(k\Delta)^2/6$, which at a 1 nm step is 1.7×10^{-5} and predicts a shift of a few tens of picometres. That is what the table shows: at 4 nm the resonances are displaced by 0.201 and 0.101 nm; refining to 3 nm collapses the deviations to $+0.0016$ and -0.00004 nm, they are unchanged at 2 nm, and both modes settle at -0.00004 nm for the 1.5 nm and the reported 1 nm steps. The collapse is faster than the bulk second-order estimate because the coarse-grid deviation is dominated by where the discrete grid lands on the layer boundaries rather than by truncation error alone; once the boundaries are resolved, the residual 0.04 pm floor is a property of the comparison and not of the solver, sitting below the 2 pm wavelength sampling and the peak estimator applied to both curves. A separate, coarser diagnostic sweep over 4, 2, 1 and 0.5 nm steps, retained in the data archive as a secondary check, shows the same collapse, from 0.158 and 0.064 nm at 4 nm to below 0.01 nm from the 1 nm step on. The honest reading is that agreement at the 1 nm step is below 0.01 nm and that the step is resolved, not that the two methods agree exactly.

The close overlay is significant because the FDFD solver discretizes the spatial Maxwell operator rather than rearranging the same layer matrices, reducing the likelihood that the agreement is caused by a shared cascade implementation [22].

The CST finite-element model reproduced the explicit 36-layer stack. Figure S7 separates the four extracted error classes for both modes: resonance-wavelength bars remain below 0.02%, FWHM and loaded-Q errors below 0.5%, and the only visibly larger bar is the mode 1 peak-transmission error at approximately 1.96% (mode 2 remains below 0.2% for the same quantity). Numerically, the mode 1 CST relative errors were -0.0192% in resonance wavelength, -0.4578% in loaded Q, $+0.4407\%$ in FWHM, and -1.9561% in peak transmission; for mode 2, the cor-

Table S8: FDFD grid convergence. Both solvers are sampled on the same 2 pm wavelength grid and the same peak estimator is applied to both, so the deviation is a difference between discretizations rather than between post-processing choices. The transfer-matrix reference is 1516.78913 and 1651.19900 nm. Deviations below the 2 pm wavelength grid are reported as computed; equal entries at adjacent grid steps mean the peak resolved to the same wavelength sample.

Grid step (nm)	λ_1 (nm)	λ_2 (nm)	Deviation (nm)	Max |deviation| (nm)
4.0	1516.98967	1651.30008	+0.20053, +0.10108	0.20053
3.0	1516.79076	1651.19897	+0.00162, −0.00004	0.00162
2.0	1516.79076	1651.19897	+0.00162, −0.00004	0.00162
1.5	1516.78910	1651.19897	−0.00004, −0.00004	0.00004
1.0 (reported)	1516.78910	1651.19897	−0.00004, −0.00004	0.00004

responding errors were +0.00314%, −0.2126%, +0.2162%, and −0.1598%. Figure S7 therefore indicates excellent agreement for the spectral locations and linewidth metrics used in sensing, with the remaining discrepancy concentrated mainly in external coupling to mode 1.

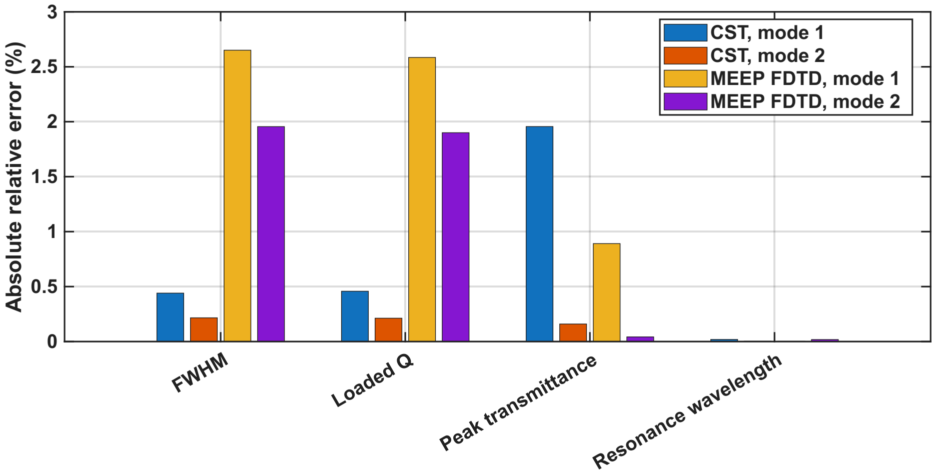

Figure S7: Nominal CST–MATLAB error audit. Bars show absolute relative error for FWHM, loaded Q, peak transmission, and resonance wavelength. The mode 1 peak-transmission difference is the largest entry; wavelength errors are the smallest. All quantities were extracted using common post-processing definitions.

All four independent ±0.5 nm ITO/TiO_2 thickness corners retained both modes; the largest absolute resonance difference within each corner was nearly uniform at 0.245–0.249 nm, with no single ITO/TiO_2 combination producing a distinct spectral failure, and loaded-Q discrepancies remained below 1.75% throughout the tolerance box (largest 0.249 nm resonance error and 1.745% loaded-Q error across the corner set). The weak corner-to-corner variation indicates that CST–MATLAB agreement is not restricted to the nominal filling fraction. The FDFD spectral overlay is given in Figure S6 and its grid audit in Table S8, and the complete four-corner resonance-wavelength and loaded-Q comparisons in Figure S8a,b.

S11.1 Independent FDTD Validation

A time-domain solve adds a third electromagnetic discretization to the FDFD and CST checks, and it differs from both in how the material enters: the dispersion is advanced as auxiliary differential equations alongside the fields rather than evaluated as a permittivity at each wavelength. Two properties of that model were gated before any comparison was read, because either could have been mistaken for a solver disagreement. The fitted media reproduce the tabulated indices the production solver evaluates to 1.4×10^{-5} in n, which corresponds to a resonance shift near 0.01 nm and therefore cannot account for a discrepancy at the nanometre scale; and a slab whose complex transmission and reflection are known in closed form is reproduced to 1.0×10^{-5} through the identical monitor and de-embedding arithmetic, so no phasor or port correction is applied to the stack comparison, unlike the CST case.

Over the full 1460–1700 nm window the transmittance RMSE against the production model was 2.06×10^{-3} and the complex S_{21} RMSE 3.49×10^{-3}, against declared bounds of 0.035 and 0.15. Per mode, the resonance-wavelength differences were 0.040 and 0.301 nm, the loaded-Q differences 2.59% and 1.90%, the FWHM differences 2.65% and 1.96%, and the peak-transmittance differences 0.0045 and 0.0003. Every one is inside the bound declared for CST, and the mode 1 wavelength difference is smaller than CST's own 0.291 nm. The four-solver figure of the article carries the FDTD spectrum on the same axes as the production model and the FDFD samples, and Figure S7 places the FDTD resonance-metric errors beside the CST ones rather than in a separate panel, so the two independent solvers can be read against each other rather than only against the production model.

Grid refinement from 1000 to 4000 pixels per micrometre moved the resonances by 0.249 nm between the two finest levels, inside the 0.60 nm bound. The sequence oscillates within about half a nanometre rather than descending monotonically, and the reason is worth stating: the layer boundaries fall at 0.01 nm multiples, so no achievable uniform grid aligns with all of them and the residual error changes sign as the grid slides across the interfaces. This FDTD model fixes the resonances to roughly a quarter of a nanometre, comfortably below the 2 nm agreement bound but not arbitrarily improvable by refining further.

The field profiles matter more here than the spectra. The physical reading of this device is that mode 1 sits in the thermo-optic reference region and mode 2 in the analyte region, and a solver that reproduced both resonance wavelengths while disagreeing about where the energy sits would undercut that reading rather than support it. The independent electric-energy fractions place 0.232 of mode 1 in the reference coating against 0.091 in the analyte, and 0.272 of mode 2 in the analyte against 0.125 in the reference coating: the asymmetry reverses between the two modes, which is what the dual-parameter separation of Section 3.5 of the article depends on. Profile correlations against the production reconstruction were 0.99999 for both modes.

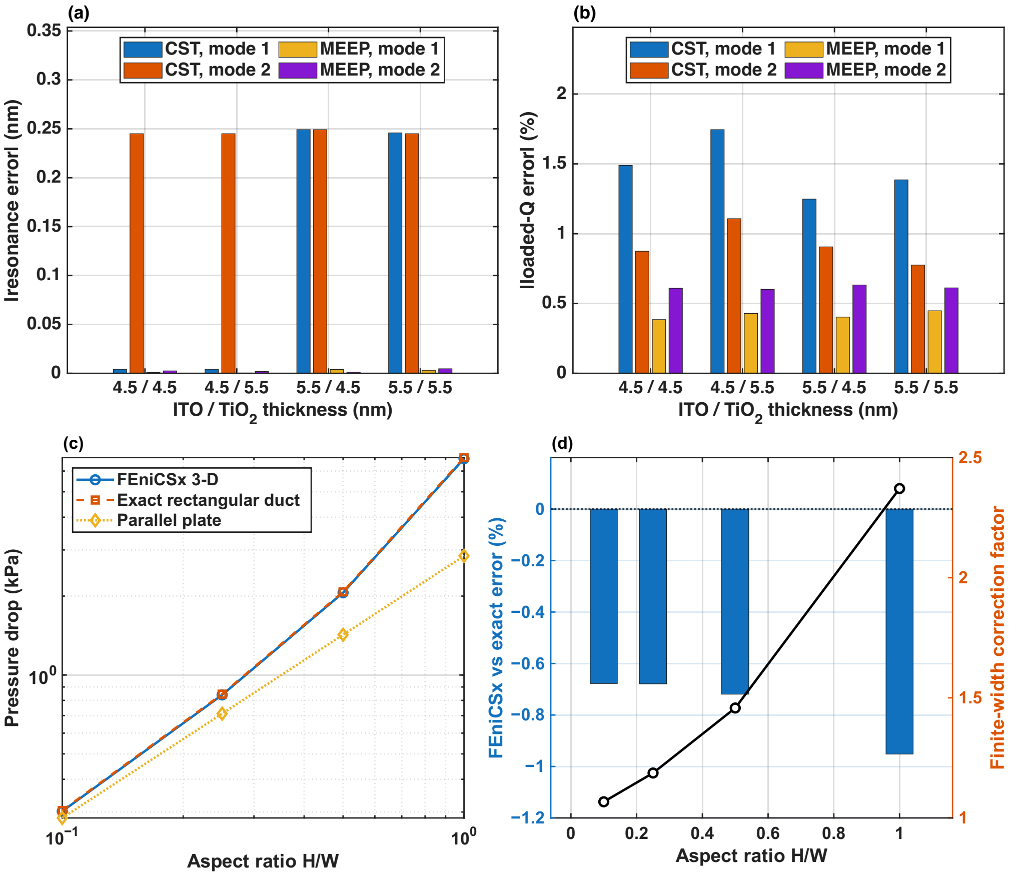

Figure S8: Agreement away from the nominal point. (**a**,**b**) CST and MEEP against the production model at the four ±0.5 nm ITO/TiO_2 thickness corners, for resonance wavelength and loaded Q; each tick gives the ITO and TiO_2 thickness of one corner in nanometres, and the largest CST discrepancies over the whole tolerance box are 0.249 nm and 1.745%. (**c**,**d**) Three-dimensional finite-width pressure validation against the analytical rectangular-duct series, and the resulting side-wall correction factor. The FEniCSx error stays below 1% while the correction factor rises from 1.067 at the nominal 500×50 μm channel to 2.371 at a square duct, which is where an uncorrected parallel-plate approximation stops being adequate.

S11.2 Independent Topological Eigensolver Audit

The most delicate claim in this work is not that two resonances exist but that they are interface states forced by a Zak-phase difference between the two half-stacks. That claim rests on a band structure, on inversion parities, and on an invariant assembled from them, all produced by the plane-wave expansion written for this study. An established block-iterative eigensolver recomputes them independently.

The bulk bands agree with the production expansion to 1.5×10^{-5} in band-scaled frequency, and the first gap runs from 1337.8 to 1842.7 nm, containing both interface states. Reaching that figure required one change to the audit rather than to either solver. This design closes its fourth gap at the zone centre for the same reason it closes its second, so bands 4 and 5 sit 10^{-4} apart at Γ; asking the eigensolver for exactly the four bands being reported left it no state above that pair, and just off Γ it returned the upper member as the fourth band. The disagreement this produced was equal, to two digits, to the band-4/5 splitting at that wave number. Solving for two bands beyond those reported removes it. Figure 4 of the article carries the independently computed bands as markers on the production dispersions. The parities require one statement of convention before they can be compared, and making it explicit is worth more than the comparison itself. At the zone centre the two codes return identical parity signs for every band of both cells. At the zone edge they return opposite signs, in all eight cases, because the parity of the full Bloch function there depends on where the Bloch phase is referenced: displacing that reference by half a period multiplies the mirrored field by $e^{i\pi}$ and flips every zone-edge sign together. An individual zone-edge parity, and therefore an individual Zak phase, is a statement about a convention as much as about a crystal. What survives the choice is the comparison between the two half-stacks, and

that is also what bulk–boundary correspondence uses. Read that way the two calculations agree exactly. Both find that the first band carries opposite zone-edge parity in the two cells, which is the band inversion; both therefore assign Zak phases differing by π, and the difference computed from the independent parities matches the production value to zero within double precision. A discrete Berry phase around the Brillouin zone, evaluated on the independent eigenvectors and never referring to a parity at all, gives a difference of 3.1393 rad, a departure from π of 0.07%. The absolute Zak phases quoted in Section 3.1 of the article and Figure 4 of the article are the production convention; the audit confirms the difference between them, not the labelling.

Two limitations belong with this result. The second band has no individual Zak phase here and none is quoted: the two layers of this design have almost equal optical thickness at the non-dispersive design indices the band-structure cell is built from, 387.600 against 387.498 nm, which closes the gap between the second and third bands at the zone centre, and a Zak phase is defined only for a band that stays separated from its neighbours. The two routes disagree for that band, which is the expected symptom of a non-isolated band rather than a discrepancy between solvers. Separately, this audit reproduces a calculation; it does not make the topological interpretation experimental, and this audit does not alter the bounded matched-control interpretation of Section 3.9 of the article.

The two-dimensional FEniCSx transport–reaction model passed all nine of its predeclared observable-level gates. For the nominal hydrodynamic quantities, the pressure-drop error was 6.30%, which is the finite-width correction the two-dimensional model does not carry, while mean velocity, residence time and wall shear agreed to numerical precision: the quadratic velocity space of the Taylor–Hood pair represents the parabolic channel profile exactly, so the computed wall shear reproduces the analytical value to round-off. For the transient surface data, normalized RMSE values were 2.29% for surface concentration, 0.50% for coverage, and 1.36% for self-referenced wavelength shift, with $R^2 = 0.997$, 0.9996, and 0.997. Across the 42-point transport map, association time gave NRMSE 8.63%, MAPE 3.74%, and $R^2 = 0.852$, whereas pressure drop gave NRMSE 0.46%, MAPE 4.35%, and $R^2 = 0.999$. Figure S9 makes these differences visible without combining incompatible units: association time and surface concentration are the largest normalized-error observables, while the transport-map pressure drop has the smallest error; most normalized points remain close to the identity line, and the largest single-point relative departures belong to the transient surface observables rather than to the transport map, reaching 64% on the self-referenced shift and 43% on surface concentration where the reference value is itself small, against 13% across the map; and association time has the weakest R^2 and therefore defines the most demanding model-reduction limitation. Mixed-unit aggregate RMSE values were deliberately excluded from interpretation: agreement is reported per observable and on that observable's own normalized scale, following the Bland–Altman argument that agreement should be assessed in the units of the measured quantity, through bias and limits of agreement rather than a scale-free summary statistic [27]. These residuals are confined to the reduced-order analyte-delivery step and do not enter any headline sensing figure of merit. The sensitivity matrix, the nonlinear calibration, the covariance- and bias-aware detection limits, and the specification yield are all computed from the optical model at a specified analyte index; the transport calculation only sets how quickly, and how uniformly, that index is established at the surface. The association time therefore bounds the surface response time and the assumption of uniform analyte loading, not the reported RI or temperature limits.

Mesh refinement from 1200 to 28,800 elements changed the plateau coverage by 0.00084% and the association t_{90} by 0.030% between the two finest levels; halving the integration step from 15 to 7.5 s changed coverage by 0.021% and t_{90} by 0.51%. The temporal increment halves at each refinement, as first-order time integration requires, so the step size is resolved rather than merely sampled. The transport–reaction discrepancies are therefore model-reduction differences rather than unresolved FEniCSx mesh or time-step errors. The surface reaction remains an idealized Langmuir model [26]. The three-dimensional finite-width calculation additionally agreed with the analytical rectangular-duct series to −0.678%, −0.679%, −0.719%, and −0.952% for $H/W = 0.10$, 0.25, 0.50, and 1.00; at the nominal 500×50 μm cross-section, FEniCSx predicted 0.30190 kPa, compared with 0.30396 kPa analytically and 0.30395 kPa from the corrected re-

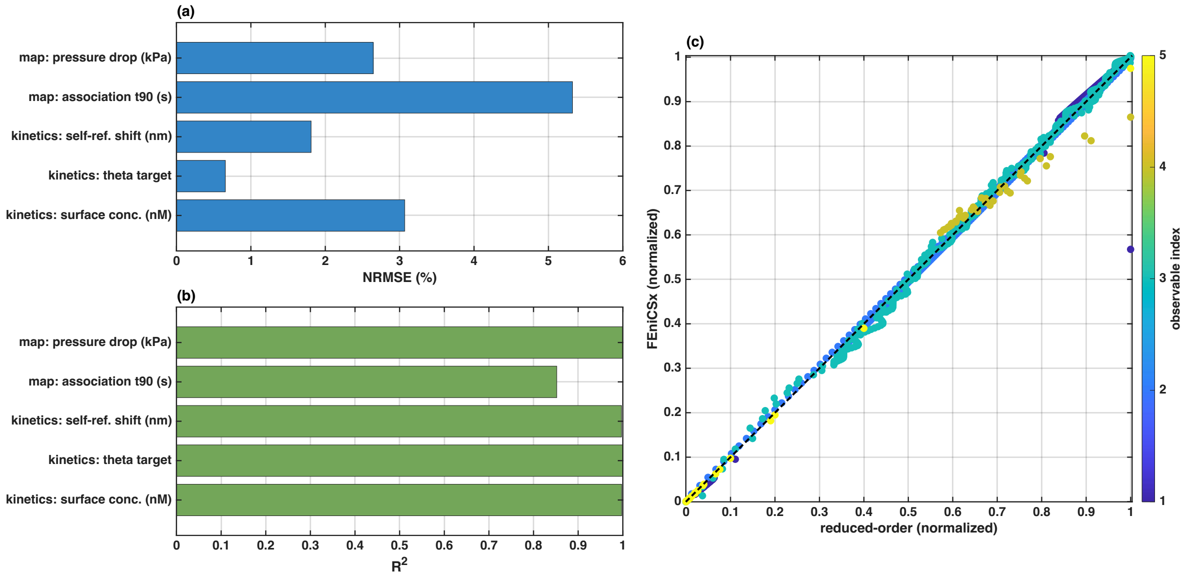

Figure S9: FEniCSx transport–reaction parity audit. (**a**) Per-observable normalized errors. (**b**) Per-observable R^2. (**c**) Combined normalized parity between FEniCSx and reduced-order outputs across all observables. Using normalized, observable-specific metrics avoids combining incompatible physical units into one misleading error statistic.

duced model. The FEniCSx error remains below 1% while the finite-width correction factor rises monotonically from 1.067 for the nominal channel to 2.371 for the square duct, which validates the side-wall correction used by the reduced model and identifies the aspect-ratio range in which an uncorrected parallel-plate approximation becomes inadequate [28]. The full 3D finite-width parity and correction-factor plots, together with the mesh- and time-step-convergence tables, are provided in Figure S8c,d.

Taken together, the S-matrix, explicit-bilayer, FDFD, CST, FEniCSx 2D, and FEniCSx 3D comparisons support numerical consistency across independent formulations. They do not constitute thin-film fabrication, optical measurement, or biological validation.

S12 Determinant of the Sensitivity Matrix from the Modal Overlaps

This section carries the algebra behind Equations (27) and (28) of the article and records what each step assumes. Steps 1 and 2 are the standard first-order result for a perturbed dielectric resonator. Steps 3 to 5 are specific to a two-mode dual-parameter stack and are what the design rule rests on.

Step 1: first-order resonance shift. For a one-dimensional resonator with permittivity $\varepsilon(z)$ and mode field $E_i(z)$, a perturbation $\delta\varepsilon(z)$ shifts the resonance by the stationary expression

$$\frac{\delta\lambda_i}{\lambda_i} = \frac{1}{2}\,\frac{\int \delta\varepsilon(z)\,|E_i(z)|^2\,\mathrm{d}z}{\int \mathrm{Re}\,\varepsilon(z)\,|E_i(z)|^2\,\mathrm{d}z}. \tag{S35}$$

This is the usual variational result for a weakly perturbed lossless cavity [29]; that reference is the apt one here because its subject is the harder case of a perturbation that moves a material boundary, which is exactly what the thermal-expansion half of θ_ℓ is. It is applied here to a lossy, dispersive and radiatively open structure, so it is an approximation on three counts: the extinction terms are dropped from the weighting integral, material dispersion is evaluated at the unperturbed wavelength, and the radiative correction accompanying a finite Q is neglected. The article measures the resulting error rather than bounding it analytically, obtaining 5.4% and 2.2% on the index column.

Step 2: region-wise overlaps. Every perturbation of interest is piecewise uniform over the functional regions of the stack. With $\Gamma_{\ell,i}$ defined as in Equation (25) of the article, a uniform index change δn_ℓ inside region ℓ gives $\delta\varepsilon_\ell = 2n_\ell \delta n_\ell$, and Equation (S35) reduces to $\delta\lambda_i/\lambda_i = \Gamma_{\ell,i}\, \delta n_\ell / n_\ell$. Summing over regions produces the two columns of $\mathbf{S}$ quoted in Equation (26) of the article. The thermal column additionally carries each region's linear expansion, because a layer that lengthens changes its optical path at constant index; the two contributions combine into the single coefficient $\theta_\ell = n_\ell^{-1} \partial n_\ell / \partial T + \alpha_\ell$. Thickness change enters at first order only through this term, which is why the budget of Table 4 of the article separates the index-driven and expansion-driven parts of every entry.

Step 3: the determinant. Writing the two columns out,

$$\det \mathbf{S} = S_{n,1}S_{T,2} - S_{n,2}S_{T,1} = \frac{\lambda_1 \Gamma_{\mathrm{a},1}}{n_\mathrm{a}}\, \lambda_2 \sum_\ell \Gamma_{\ell,2}\theta_\ell - \frac{\lambda_2 \Gamma_{\mathrm{a},2}}{n_\mathrm{a}}\, \lambda_1 \sum_\ell \Gamma_{\ell,1}\theta_\ell. \tag{S36}$$

Both terms carry $\lambda_1\lambda_2/n_\mathrm{a}$, and factoring that out together with $\Gamma_{\mathrm{a},1}\Gamma_{\mathrm{a},2}$ leaves

$$\det \mathbf{S} = \frac{\lambda_1\lambda_2}{n_\mathrm{a}}\, \Gamma_{\mathrm{a},1}\Gamma_{\mathrm{a},2} \sum_\ell \left(\frac{\Gamma_{\ell,2}}{\Gamma_{\mathrm{a},2}} - \frac{\Gamma_{\ell,1}}{\Gamma_{\mathrm{a},1}} \right) \theta_\ell, \tag{S37}$$

which is Equation (27) of the article. This cancellation is the substantive step and it is exact within the first-order model: the absolute overlaps leave the expression entirely, so no amount of field concentration in any one region can by itself make the matrix invertible.

Step 4: the analyte term vanishes identically. The sum formally runs over the analyte region too, where $v_{\mathrm{a},i} = \Gamma_{\mathrm{a},i}/\Gamma_{\mathrm{a},i} = 1$ for both modes, so that term contributes $(1-1)\theta_\mathrm{a} = 0$. The analyte sets the scale of the index column and then drops out of the determinant entirely. This follows from the definition of $v_{\ell,i}$ rather than from any modelling choice, and it holds whatever value the analyte thermo-optic coefficient takes, which is why the article's separability argument is unaffected by that coefficient being set to zero in the forward model.

Step 5: the singular locus. Using $S_{n,1}S_{n,2} = \lambda_1\lambda_2\Gamma_{\mathrm{a},1}\Gamma_{\mathrm{a},2}/n_\mathrm{a}^2$ to replace the prefactor, and splitting the reference coating out of the sum, Equation (S37) becomes Equation (28) of the article. Setting it to zero and dividing through by $S_{n,1}S_{n,2}n_\mathrm{a}$, which is nonzero whenever both modes couple to the analyte at all, leaves

$$\theta_\mathrm{REF}\, \Delta x + \Delta y = 0 \qquad \Longleftrightarrow \qquad \frac{\Delta y}{-\theta_\mathrm{REF}} = \Delta x. \tag{S38}$$

On the axes $(\Delta x,\ \Delta y/(-\theta_\mathrm{REF}))$ this is the diagonal, which is how the design map of the article is drawn. The slope contains no geometric quantity, so the locus is a materials property: changing layer thicknesses moves a design within the plane but cannot move the line. Sweeping $\partial n_\mathrm{ref}/\partial T$ instead rotates the line through a fixed cloud of designs, which is the sweep of Section 3.10 of the article and the reason its crossing is a materials specification rather than a tolerance.

What the relation predicts, and what it does not. Because $\det \mathbf{S}$ is proportional to a difference of terms that nearly cancel in this stack, the relative accuracy of the predicted determinant is far worse than that of either column taken alone. The article measures both: the index column is reproduced to a few percent, while the determinant carries a median relative error of 24.4% away from the locus and 32.7% near it. The relation is accordingly used for which side of the locus a design falls on and for the ordering of the margins, both of which are tested against the forward model, and not as a numerical predictor of $\det \mathbf{S}$.

S13 Supporting Results Floats

The article refers to each float below by its supplementary number. They are collected here rather than distributed through the article because none of them carries a result the article does not already state; they show the same audits at full size, and the tabulated control family at full length.

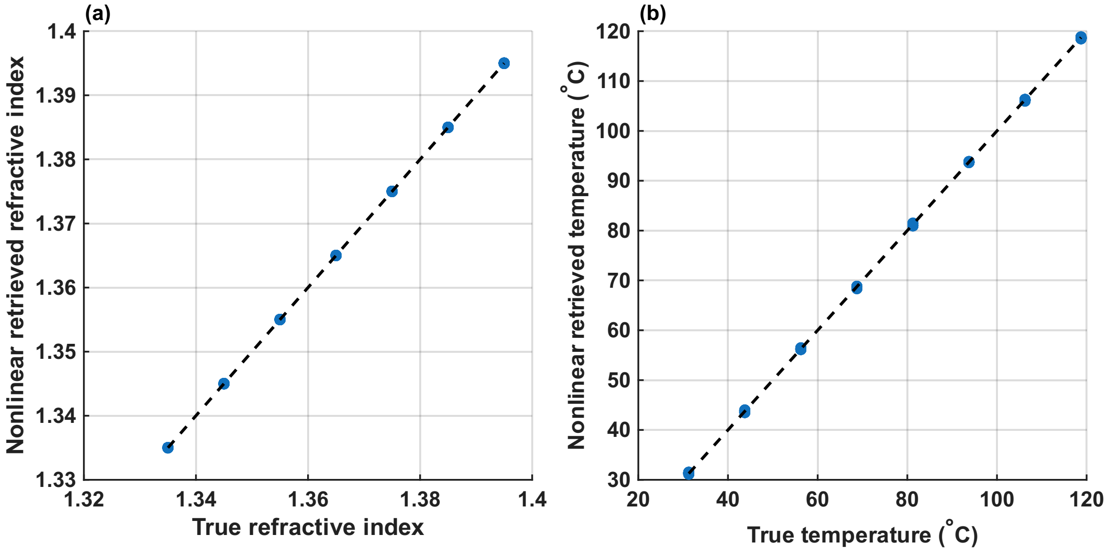

Figure S10: Held-out parity of the bounded nonlinear inversion, evaluated on the interleaved validation grid rather than on the calibration points, and under the readout-noise model rather than on noise-free wavelengths. (**a**) Refractive-index retrieval, RMSE 2.43×10^{-5} RIU. (**b**) Temperature retrieval, RMSE 0.155 °C. Both estimates follow the identity line across the full validation range instead of only near the nominal operating point, and neither shows the edge divergence that a single local sensitivity matrix would produce at the domain boundaries.

Table S9: The admissible Protocol 1 control family. Every row is a topologically trivial stack that independently satisfies the same predeclared match gate as the reported control and was exposed to the same 250 disorder vectors; the topological arm is common to all rows and yields 0.952. Rows are ordered by match quality, not by outcome. The starred row is the control reported in the text and in Figure 15 of the article.

Control	d_{HMM} (nm)	Fill f	λ_1, λ_2 (nm)	Q_1, Q_2	Y_{trivial}, ΔY
1 (reported)*	14.16	0.501	1518.8, 1647.8	195.4, 221.6	0.580, 0.372
2	15.11	0.520	1519.7, 1647.4	193.1, 216.4	0.260, 0.692
3	13.34	0.543	1518.2, 1648.3	195.2, 223.2	0.648, 0.304
4	13.61	0.520	1518.5, 1647.2	196.2, 222.2	0.536, 0.416
5	15.19	0.450	1519.7, 1647.0	195.5, 219.7	0.508, 0.444
6	17.88	0.422	1519.9, 1645.8	189.3, 213.2	0.516, 0.436
7	15.19	0.491	1519.5, 1647.7	193.3, 218.6	0.458, 0.494
8	10.32	0.574	1514.5, 1652.1	215.5, 222.7	0.480, 0.472
9	14.51	0.488	1518.7, 1647.0	194.8, 220.7	0.556, 0.396
10	18.72	0.425	1520.2, 1646.2	186.5, 211.2	0.524, 0.428
11	15.06	0.475	1518.6, 1647.1	193.4, 220.3	0.564, 0.388
12	13.65	0.556	1518.1, 1647.6	193.7, 221.3	0.600, 0.352
13	12.60	0.580	1517.6, 1648.1	195.8, 224.4	0.636, 0.316
14	11.33	0.577	1517.8, 1649.1	200.8, 228.9	0.684, 0.268

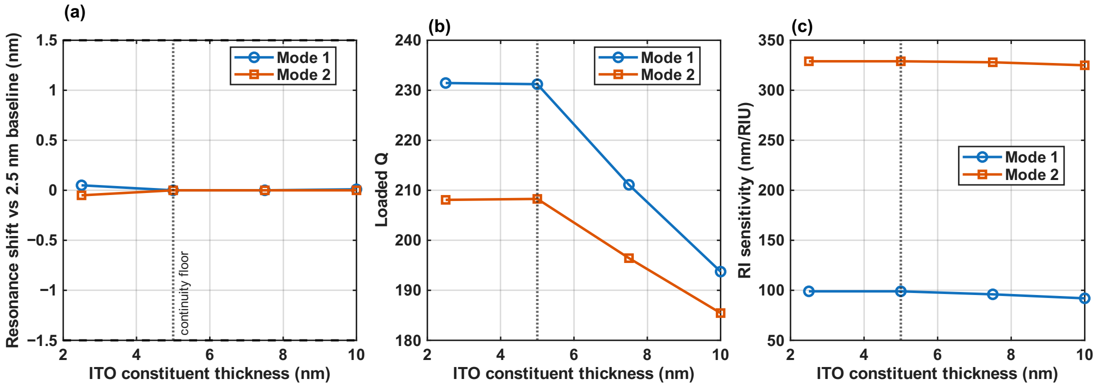

Figure S11: Constituent-thickness audit of the ITO/TiO_2 nanolaminate, swept from 2.5 to 10 nm with the cavity retuned at each point. (**a**) Resonance preservation: retuning recovers both resonance wavelengths across the whole sweep, so wavelength alone does not limit the design. (**b**) Loaded Q, which is the quantity that does: it holds to the declared 5 nm floor and then falls for both modes, most sharply between 5 and 10 nm as absorption and coupling perturbations grow. (**c**) Refractive-index sensitivity; mode 2 stays near 330 nm/RIU throughout while mode 1 degrades at the thickest realization. The vertical marker is the design floor used in the numerical audit, not a demonstrated physical continuity threshold.

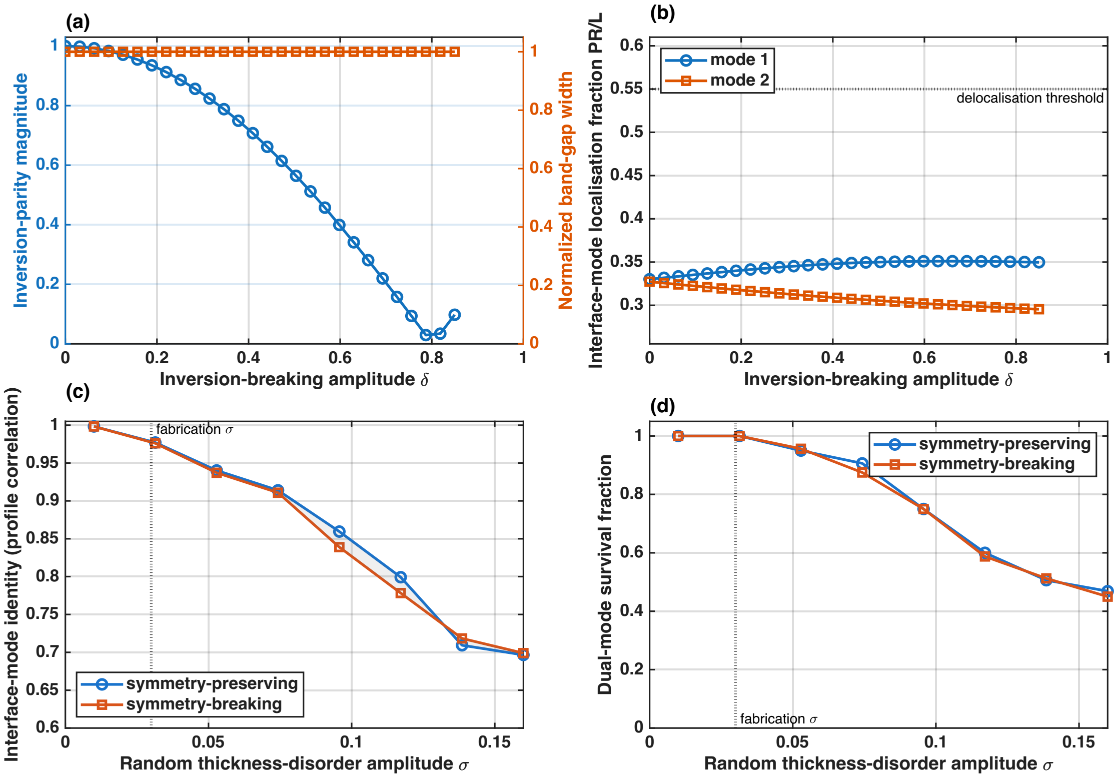

Figure S12: The limits of the topological evidence. (**a**) Under deterministic inversion-breaking detuning the bulk parity order parameter falls continuously while the normalized gap width barely moves, so parity is the sensitive quantity and gap persistence is not. (**b**) The interface state nonetheless stays localized through that same pure detuning, its participation ratio changing only modestly. (**c**) Against random disorder the mode-identity correlations of symmetry-preserving and symmetry-breaking ensembles fall almost together, the peak separation reaching only $\Delta = 0.021$. (**d**) Modal survival falls from 1.00 to 0.47 and 0.45 for the two ensembles over the same disorder range. Gap persistence, finite-mode survival and sensing-mode identity are three distinct statements: gap persistence alone does not establish protected sensing functionality, and no resolvable symmetry-based protection of modal identity is found over most of the tested disorder range.

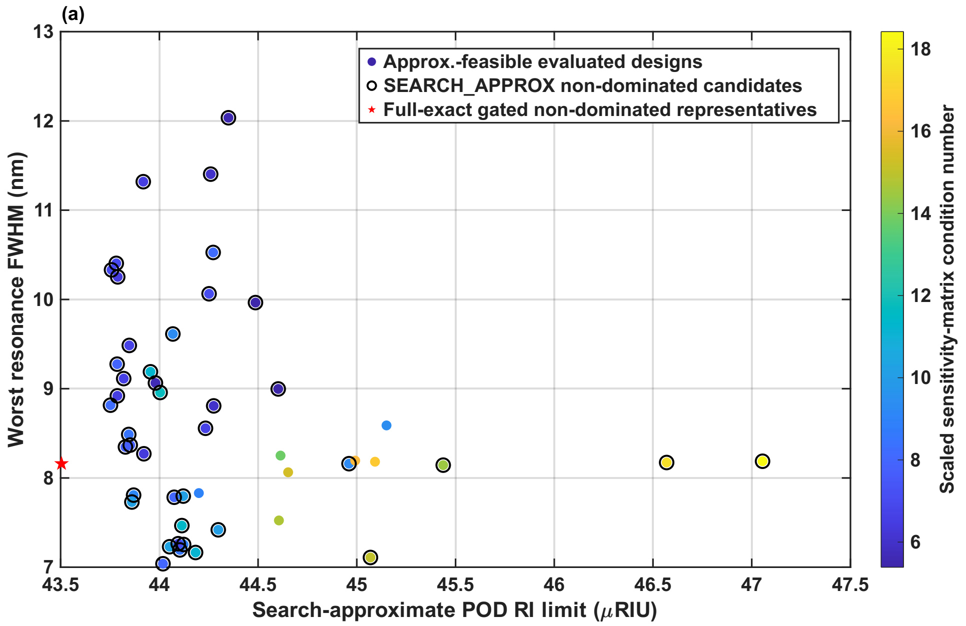

Figure S13: Search-approximate Pareto front with the full-exact gated representatives overlaid; marker colour is the scaled sensitivity-matrix condition number. The selected reference design sits near the low-linewidth boundary of the feasible cloud rather than at the minimum of either projected axis, because selection was controlled by the complete feasibility set including variables that no two-dimensional projection shows.

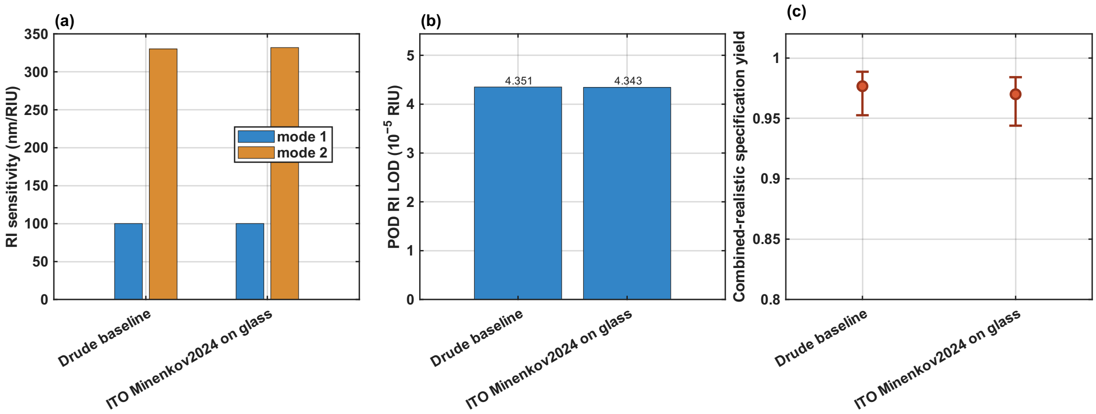

Figure S14: Measured-ITO substitution. (**a**) The two RI sensitivities remain close to the Drude-model values. (**b**) The full-covariance RI POD-LOD changes only slightly. (**c**) The 300-sample specification yields and Wilson intervals overlap. The comparison is a material-grounded numerical sensitivity analysis, not experimental validation of a fabricated sensor.

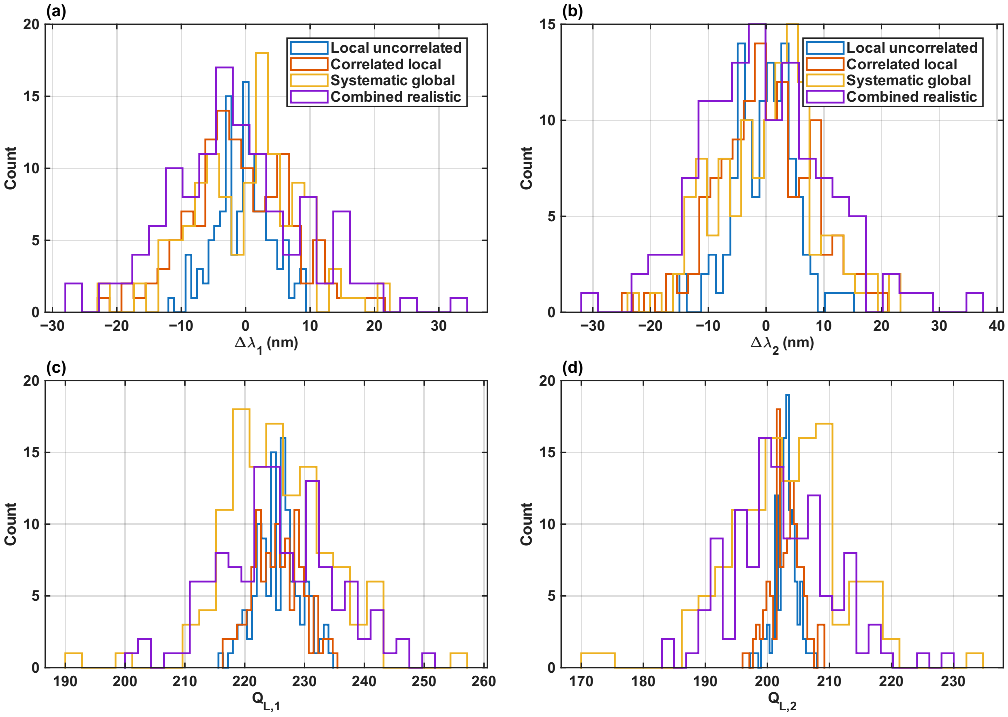

Figure S15: Behaviour of the selected operating point under the fabrication ensembles. (**a**,**b**) Spectral-offset distributions of modes 1 and 2 by scenario. (**c**,**d**) Loaded-Q distributions of the same two modes. Local uncorrelated errors give narrow distributions, while systematic-global and combined-realistic errors broaden the tails; mode 2 shows the wider low-Q response, consistent with its stronger dependence on analyte-side modal overlap and on the lossy nanolaminate environment.

S14 Robustness of the Reported Results to the Declared Assumptions

This section reports how the results of the article move when the inputs and the choices they were computed under are varied: the instrument budget behind the detection limits, the amount of per-device calibration, the incidence angle, the interface quality, the trivial control chosen for comparison, and the fabrication inputs that scatter the sensitivity matrix. Every one uses the frozen design, the production forward model and the production covariance, probability-of-detection and fabrication routines; the scripts are included in the archived reproducibility package.

S14.1 Instrument-Noise and Drift Scenarios

Table S10 defines the scenarios of Figure 13d of the article and gives the resulting limits. Figure S16 shows the joint two-peak fit behind the spectral-fit term of the budget: the 350 repeated fits, the resolution bias of the fitted centres and the Cramér–Rao bound.

Table S10: Instrument-class and drift scenarios used in the sensitivity analysis of the detection limits. Each row changes the declared inputs stated in its label and leaves every other input at its declared value; σ_λ are the resulting per-device wavelength standard deviations, and the last two columns give the limits relative to the declared model S0.

**Scenario**	σ_{λ_1} **(pm)**	σ_{λ_2} **(pm)**	**LOD, RI (RIU)**	**LOD, T (°C)**	**Ratio RI**	**Ratio T**
S0 declared model: tunable laser, 1 pm reference	1.76	3.48	4.36×10^{-5}	0.123	1.00	1.00
S1 tunable laser, 5 pm wavelength accuracy	5.29	6.08	6.52×10^{-5}	0.378	1.50	3.07
S2 spectrum analyzer: 10 pm accuracy, 50 pm resolution	10.30	10.72	1.08×10^{-4}	0.738	2.48	5.99
S3 compact spectrometer: 20 pm, 3×10^{-3} noise, 0.1 °C stage, 10^{-4} RIU standard	23.04	38.89	4.69×10^{-4}	1.554	10.76	12.62
S4 stage stability relaxed to 0.1 °C	5.00	3.80	4.36×10^{-5}	0.431	1.00	3.50
S5 index standard relaxed to 10^{-4} RIU	9.19	32.96	4.22×10^{-4}	0.123	9.69	1.00
S6 uncorrected zero drift, 5 pm per mode	5.30	6.09	8.59×10^{-5}	0.518	1.97	4.21
S7 uncorrected common-mode drift, 5 pm	5.30	6.09	6.44×10^{-5}	0.372	1.48	3.03
S8 field combination of S1, S4, S5 and S6	12.47	33.73	4.31×10^{-4}	0.747	9.90	6.07
S9 calibration-standard terms removed	1.43	1.10	1.08×10^{-5}	0.116	0.25	0.94
S10 standard and fit terms removed: reference and thermal floor	1.42	1.09	1.05×10^{-5}	0.114	0.24	0.93

S14.2 Reduced Per-Device Calibration

Table S11 lists the retrieval errors behind Figure 14 of the article. Thirty devices were drawn from the combined-realistic fabrication scenario with the production sampler; for each device the two resonance surfaces were solved exactly on the 8×9 calibration grid and the 56-point validation grid, the reference measurements required by each strategy were solved exactly, and the bounded inversion of the article was run on the corrected nominal surface. No readout noise was added.

S14.3 Oblique-Incidence Test of the Hyperbolicity Condition

Figure S17 and Table S12 compare the nominal hyperbolic nanolaminate, an isotropic-equivalent layer with $\varepsilon_\perp$ replaced by $\varepsilon_\parallel$, and the stack without the nanolaminate, over 0–30° in both polarizations. Both resonances were tracked by continuity with a 0.05 nm spectral step, and the local RI sensitivity was obtained by central differences of ±0.005 RIU at each angle. In TE the two tensor treatments coincide to machine precision, as the TE wave equation does not contain $\varepsilon_\perp$.

S14.4 Interface Roughness and Interdiffusion Sweep

Table S13 gives the deterministic interface-mixing sweep of the article's fabrication model at the nominal fill fraction. Every interface of the stack is replaced by a graded interlayer twice the RMS roughness thick, capped at 30% of the thinner adjacent layer, so the 3 nm row corresponds to a 6 nm mixed layer between the dielectric mirror layers and a 1.5 nm mixed layer on each side

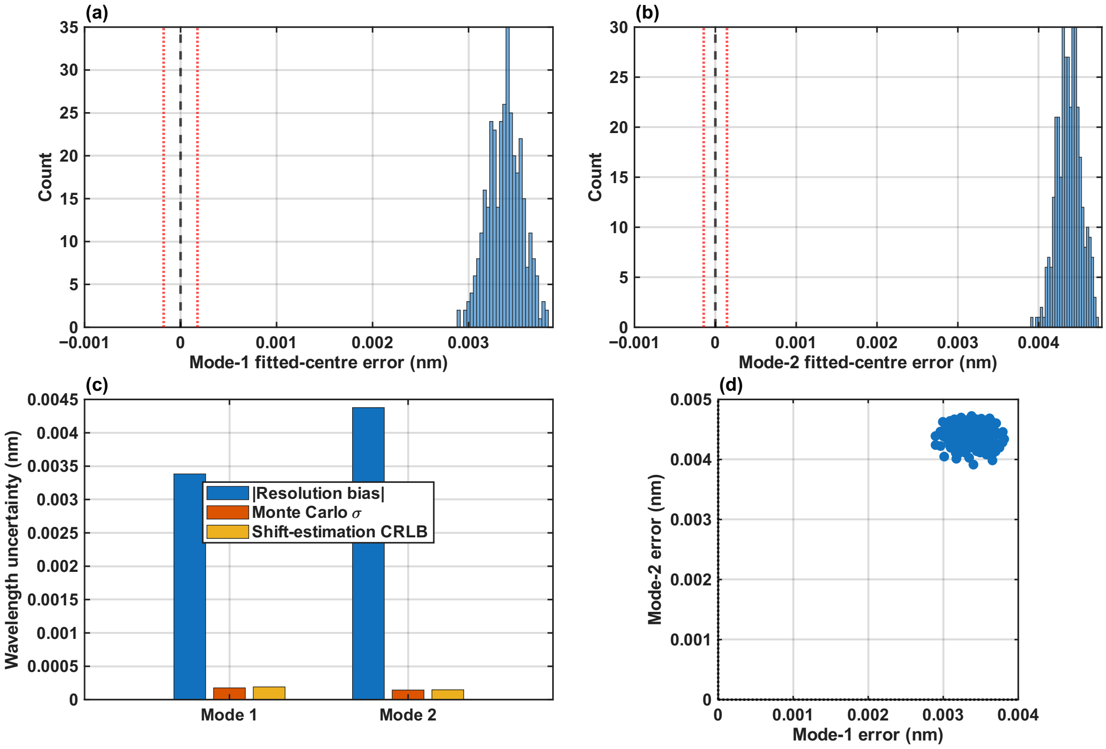

Figure S16: Joint two-peak fit statistics behind the spectral-fit covariance. (**a**,**b**) Histograms of the fitted-centre error of modes 1 and 2 over 350 simulated acquisitions under the declared readout model, with the true centre (dashed) and the $\pm 1\sigma$ band (dotted); the offset of the distributions from zero is the resolution bias introduced by the 0.020 nm instrument response acting on the asymmetric line shape. (**c**) Resolution bias, empirical fit standard deviation and Cramér–Rao bound per mode. (**d**) Joint scatter of the two centre errors. The bias is carried in the bias-aware minimum detectable change; the standard deviations are the spectral-fit entries of the wavelength covariance.

Table S11: Retrieval error under seven per-device calibration strategies, pooled over 30 perturbed devices and the 56 validation points each. The near-zero columns restrict the pool to the validation points within one calibration-grid interval of the zero point. The last column is the fraction of retrievals that did not terminate on the domain boundary.

Strategy	Points	RMSE, RI (RIU)	RMSE, T (°C)	Near zero, RI	Near zero, T	In domain
A transferred nominal calibration (no device measurement)	0	2.59×10^{-2}	51.681	2.89×10^{-2}	59.524	0.14
B per-device zero point only (1 point)	1	1.89×10^{-3}	16.934	3.30×10^{-4}	3.234	0.85
C zero + affine slope correction, small span (3 points, 0.01 RIU / 12.5 °C)	3	3.23×10^{-4}	2.526	3.75×10^{-5}	0.227	0.99
D zero + affine slope correction, wide span (3 points, 0.04 RIU / 50 °C)	3	2.03×10^{-4}	1.268	5.03×10^{-5}	0.270	1.00
E zero + bilinear correction, wide span (4 points)	4	8.96×10^{-5}	0.658	5.16×10^{-5}	0.277	1.00
F zero + quadratic correction, wide span (9 points)	9	7.79×10^{-5}	0.482	4.67×10^{-5}	0.208	1.00
G full per-device surface (72 points)	72	2.54×10^{-5}	0.172	3.85×10^{-5}	0.255	1.00

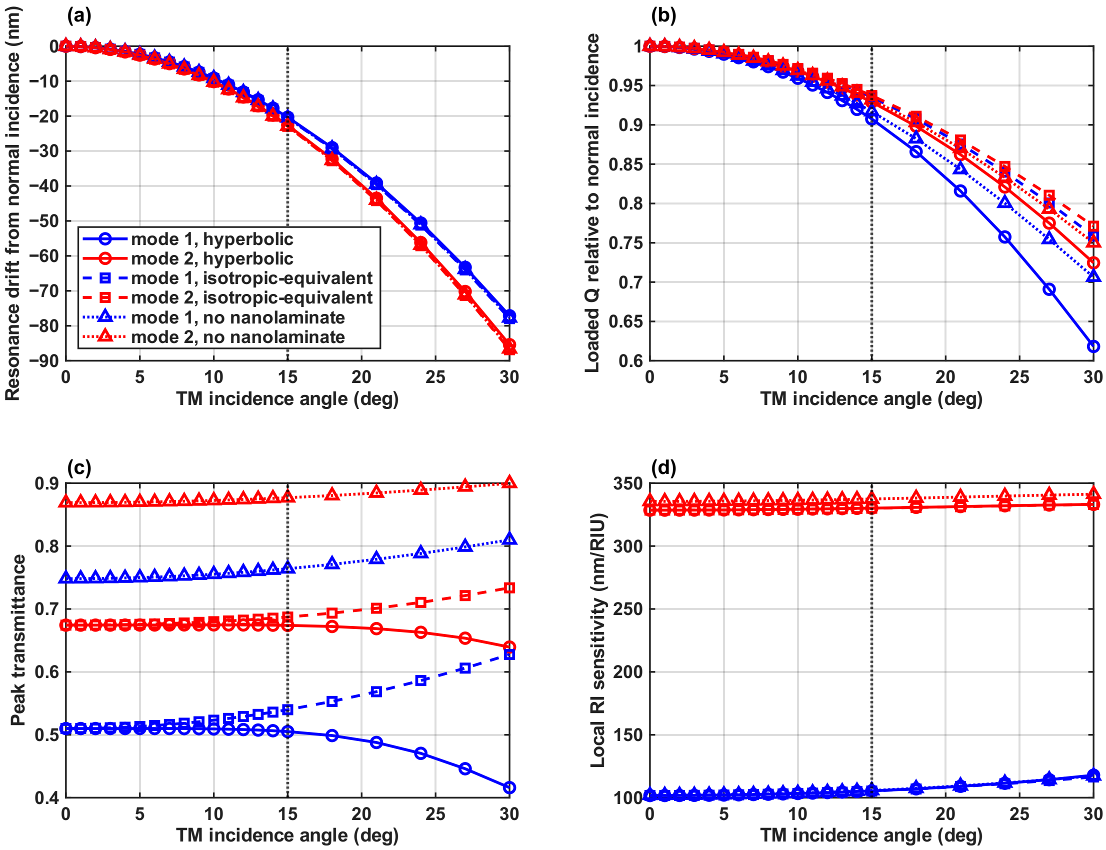

Figure S17: TM angular response of the three nanolaminate treatments. (**a**) Resonance drift from normal incidence. (**b**) Loaded-Q retention. (**c**) Peak transmittance. (**d**) Local RI sensitivity. Circles, hyperbolic tensor; squares, isotropic-equivalent layer; triangles, no nanolaminate; blue, mode 1; red, mode 2. The dotted line marks the declared 15° operating envelope.

Table S12: Angular-robustness summary of the three nanolaminate treatments over the declared 0–15° envelope and the extended 0–30° range. Paired entries are mode 1 / mode 2; retentions are the worst-case ratio to the normal-incidence value.

Pol.	Treatment	θ_{max} (deg)	RMS drift (nm)	Max drift (nm)	Min Q ret.	Min T ret.	Min S_n (nm/RIU)
TM	hyperbolic	15	9.57 / 10.64	20.31 / 22.59	0.908 / 0.929	0.991 / 0.999	101.7 / 328.6
TM	hyperbolic	30	27.91 / 30.96	77.13 / 85.40	0.618 / 0.724	0.816 / 0.948	101.7 / 328.6
TM	isotropic-equiv.	15	9.68 / 10.81	20.55 / 22.94	0.935 / 0.937	1.000 / 1.000	101.7 / 328.6
TM	isotropic-equiv.	30	28.22 / 31.46	77.95 / 86.84	0.759 / 0.771	1.000 / 1.000	101.7 / 328.6
TM	no nanolaminate	15	9.66 / 10.77	20.50 / 22.86	0.917 / 0.932	1.000 / 1.000	102.0 / 335.5
TM	no nanolaminate	30	28.14 / 31.36	77.72 / 86.58	0.706 / 0.750	1.000 / 1.000	102.0 / 335.5
TE	hyperbolic	15	8.22 / 10.13	17.46 / 21.53	1.000 / 1.000	0.961 / 0.960	101.7 / 328.6
TE	hyperbolic	30	24.08 / 29.81	66.68 / 82.70	1.000 / 1.000	0.843 / 0.830	101.7 / 328.6
TE	isotropic-equiv.	15	8.22 / 10.13	17.46 / 21.53	1.000 / 1.000	0.961 / 0.960	101.7 / 328.6
TE	isotropic-equiv.	30	24.08 / 29.81	66.68 / 82.70	1.000 / 1.000	0.843 / 0.830	101.7 / 328.6
TE	no nanolaminate	15	8.30 / 10.19	17.63 / 21.65	1.000 / 1.000	0.992 / 0.993	102.0 / 335.5
TE	no nanolaminate	30	24.34 / 29.99	67.42 / 83.22	1.000 / 1.000	0.969 / 0.970	102.0 / 335.5

of the 5 nm nanolaminate constituents. Interdiffusion at the ITO/TiO_2 interfaces is represented by the same mixed layers.

Table S13: Interface-mixing sweep at fill fraction 0.50. Shifts are relative to the sharp-interface row; loaded Q is the value returned by the fabrication-model line-shape fit.

RMS (nm)	λ_1 (nm)	λ_2 (nm)	$\Delta\lambda_1$	$\Delta\lambda_2$	T_1	T_2	Q_1	Q_2	ΔQ (%)
0.0	1516.789	1651.199	+0.000	+0.000	0.5099	0.6743	226.29	203.13	+0.00 / +0.00
0.5	1516.789	1651.199	+0.000	+0.000	0.5100	0.6744	226.27	203.11	−0.01 / −0.01
1.0	1516.789	1651.199	+0.000	+0.000	0.5101	0.6744	226.20	203.07	−0.04 / −0.03
1.5	1516.789	1651.199	+0.000	+0.000	0.5102	0.6746	226.08	202.99	−0.09 / −0.07
2.0	1516.711	1651.199	−0.078	+0.000	0.5104	0.6747	225.90	202.88	−0.17 / −0.12
2.5	1516.711	1651.199	−0.078	+0.000	0.5108	0.6749	225.73	202.74	−0.25 / −0.19
3.0	1516.709	1651.199	−0.080	+0.000	0.5111	0.6752	225.50	202.56	−0.35 / −0.28

S14.5 Jointly Matched Trivial Control

The extended search evaluated 19,080 trivial stacks over nine period configurations, a 200–1300 nm analyte cavity, a 150–900 nm reference cavity, a 5–22 nm nanolaminate, fill 0.42–0.58 and a coupling scale of 0.70–1.40, with the Protocol 1 match gate and the Protocol 2 sensitivity gate applied jointly. One candidate passed both: $N_{\text{out}} = 4$, $N_{\text{mid}} = 2$, $d_{\text{n}} = 374.8$ nm, $d_{\text{r}} = 838.2$ nm, $d_H = 12.7$ nm, $f = 0.428$, $c_\kappa = 0.938$. Table S14 compares its nominal properties with the topological stack, both evaluated with the fitted-slope routine of the article, and gives the outcome of the 250-sample paired benchmark. The loaded Q values in the table are extracted from the sampled full width at half maximum in the same local spectral window for both stacks, and they are the values quoted in Section 3.9 of the article; the coarser search-stage estimator that applies the Protocol 1 gate returned 230.5 and 202.2 for the control (`Table_R1_joint_control_frontier.csv` of the reproducibility package) against the 234.2 and 205.3 tabulated here, a difference of the same origin as that between the locally estimated and the fitted RI sensitivities. The topological arm passed 239 of 250 samples in this run, one more than in the Protocol 1 run of the article.

Table S14: The jointly matched control against the topological stack. Paired entries are mode 1 / mode 2; sensitivities are fitted over the calibrated interval with the routine of the article, and the loaded Q values come from the same resonance-metric routine for both stacks (the search-stage gate estimator gives 230.5 and 202.2 for the control; see text). Under the 250 paired disorder vectors the control passed the complete specification in 250 samples and the topological stack in 239, a paired difference of -0.044 with a 95% bootstrap interval of -0.072 to -0.020; the 11 discordant pairs all favour the control and all fail the conditioning gate, and the exact McNemar test gives $p = 9.8 \times 10^{-4}$. Both arms remained fully trackable with a minimum mode identity above 0.92.

**Quantity**	**Topological stack**	**Jointly matched control**
Resonance wavelengths (nm)	1516.8 / 1651.2	1516.0 / 1652.3
Loaded Q	233.1 / 211.3	234.2 / 205.3
Peak transmittance	0.510 / 0.674	0.595 / 0.589
S_n, fitted (nm/RIU)	90.7 / 329.4	93.6 / 335.1
S_T, fitted (nm/°C)	−0.0478 / −0.0156	−0.1127 / −0.0369
Analyte overlap Γ_{a}	0.092 / 0.270	0.098 / 0.267
Reference overlap Γ_{ref}	0.231 / 0.124	0.374 / 0.185
$\det \mathbf{S}$	14.33	34.30
Scaled condition number	8.20	3.67
Total thickness (nm)	5407	5472
Specification yield, 250 paired samples	0.956	1.000
Median scaled condition number over the samples	8.79	4.01
Worst scaled condition number over the samples	45.0	13.4

S14.6 Drivers of the Sensitivity-Slope Scatter

Table S15 reports, for each entry of the sensitivity matrix, the five fabrication inputs with the largest standardized regression coefficients over the 480 valid samples of the article's fabrication ensemble, computed exactly as the wavelength-driver analysis of Section 3.10 of the article.

Table S15: Leading drivers of the scatter of each sensitivity-matrix entry across the fabrication ensemble. Entries are the input followed by its standardized regression coefficient.

**Rank**	$S_{n,1}$	$S_{n,2}$	$S_{T,1}$	$S_{T,2}$
1	reference index (+0.18)	TiO_2 index (+0.25)	reference $\partial n/\partial T$ (−0.80)	reference $\partial n/\partial T$ (−0.67)
2	analyte-cavity thickness (−0.14)	analyte-cavity thickness (+0.20)	reference expansion (+0.26)	reference expansion (+0.21)
3	reference-cavity thickness (+0.10)	reference index (−0.17)	TiO_2 $\partial n/\partial T$ (+0.15)	TiO_2 $\partial n/\partial T$ (+0.19)
4	TiO_2 index (−0.10)	nanolaminate fill (+0.13)	analyte-cavity thickness (−0.09)	analyte-cavity thickness (+0.09)
5	nanolaminate thickness (+0.10)	reference-cavity thickness (−0.08)	TiO_2 index (−0.07)	reference index (−0.06)

S15 Microfluidic Integration and Surface-Binding Transfer

Section 4.3 of the article summarises in one paragraph two reduced-order checks that connect the optical model to a physical implementation: the reconciliation of the transport geometry with the optical geometry, and the transfer from surface binding to resonance shift. Their full statement, relocated from the article, follows.

The transport calculations answer a different question and must be reconciled with the optical geometry, since the two differ by a factor of 83: the transport model computes Langmuir coverage in a channel 50 µm high, whereas the optical model senses the bulk index of a 600 nm layer. Both describe one architecture, a delivery channel feeding a thin sensing layer. The 50 µm channel sets the pressure drop, the Péclet number and the depletion time (a 600 nm channel would be unusable there because the pressure drop scales as h^{-3}), and the analyte reaches the sensing region through a short vertical access and binds on the walls of the 600 nm cavity where the mode is. Fabricating that means releasing the cavity through etch access holes in the upper mirror, a standard surface-micromachining sequence whose failure modes here are stiction of the released membrane [30], bubble trapping at this aspect ratio and the mechanical stability of a 1715 nm dielectric stack spanning the opening. None is addressed by the present calculations, and all are prerequisites rather than refinements.

Binding on a wall is also not a change of bulk index, so the transfer was computed rather than assumed: an adlayer of thickness t_{ad} and index n_{ad} was inserted on each cavity wall and both modes relocated. For a densely packed protein film, $n_{\mathrm{ad}} = 1.53$ and $t_{\mathrm{ad}} = 1$ nm, close to 1 ng/mm^2, the shifts are 0.098 and 0.126 nm, equivalent to uniform index changes of 1.08×10^{-3} and 3.83×10^{-4} RIU; the second is within 16% of a two-wall mixing estimate, while for mode 1 the exact value is 1.6 times larger because the adlayer sits where that mode has proportionally more field. The response is linear in coverage over the 0.25–2 nm range tested. Inverting the relation gives a coverage detection limit of 0.040 nm of adlayer, about 0.054 ng/mm^2 or 1.8×10^{-9} mol/m^2 for a 30 kDa target at an assumed film density of 1.35 g/cm^3. That is a specification of the optical readout alone; the receptor chemistry, surface coverage and selectivity it assumes have not been established.

References

[1] Zhang, Z.; Zhao, P.; Lin, P.; Sun, F. Thermo-Optic Coefficients of Polymers for Optical Waveguide Applications. *Polymer* **2006**, *47*, 4893–4896. https://doi.org/10.1016/j.polymer.2006.05.035.

[2] Bednarski, H.; Hajduk, B. Dispersion of Thermo-Optic Coefficient of Optical Polymers. *Polymer* **2021**, *230*, 124074. https://doi.org/10.1016/j.polymer.2021.124074.

[3] Schneider, F.; Draheim, J.; Kamberger, R.; Wallrabe, U. Process and Material Properties of Polydimethylsiloxane (PDMS) for Optical MEMS. *Sens. Actuators A Phys.* **2009**, *151*, 95–99. https://doi.org/10.1016/j.sna.2009.01.026.

[4] Markos, C.; Vlachos, K.; Kakarantzas, G. Bending Loss and Thermo-Optic Effect of a Hybrid PDMS/Silica Photonic Crystal Fiber. *Opt. Express* **2010**, *18*, 24344–24351. https://doi.org/10.1364/OE.18.024344.

[5] Minenkov, A.; Hollweger, S.; Duchoslav, J.; Erdene-Ochir, O.; Weise, M.; Ermilova, E.; Hertwig, A.; Schiek, M. Monitoring the Electrochemical Failure of Indium Tin Oxide Electrodes via Operando Ellipsometry Complemented by Electron Microscopy and Spectroscopy. *ACS Appl. Mater. Interfaces* **2024**, *16*, 9517–9531. https://doi.org/10.1021/acsami.3c17923.

[6] Polyanskiy, M.N. Refractiveindex.info Database of Optical Constants. *Sci. Data* **2024**, *11*, 94. https://doi.org/10.1038/s41597-023-02898-2.

[7] Bi, R.; Zheng, C.; Yu, W.W.; Zheng, W.; Wang, D. Breaking through the Plasma Wavelength Barrier to Extend the Transparency Range of Ultrathin Indium Tin Oxide Films into the Far Infrared. *J. Appl. Phys.* **2023**, *134*, 165301. https://doi.org/10.1063/5.0165653.

[8] Kim, D.; Lee, S. Persistent Metallic Sn-Doped In_2O_3 Epitaxial Ultrathin Films with Enhanced Infrared Transmittance. *Sci. Rep.* **2020**, *10*, 4957. https://doi.org/10.1038/s41598-020-61772-y.

[9] Elser, J.; Podolskiy, V.A.; Salakhutdinov, I.; Avrutsky, I. Nonlocal Effects in Effective-Medium Response of Nanolayered Metamaterials. *Appl. Phys. Lett.* **2007**, *90*, 191109. https://doi.org/10.1063/1.2737935.

[10] Redheffer, R. On the Relation of Transmission-Line Theory to Scattering and Transfer. *Journal of Mathematics and Physics* **1962**, *41*, 1–41. https://doi.org/10.1002/sapm19624111.

[11] Cotter, N.P.K.; Preist, T.W.; Sambles, J.R. Scattering-Matrix Approach to Multilayer Diffraction. *J. Opt. Soc. Am. A* **1995**, *12*, 1097–1103. https://doi.org/10.1364/JOSAA.12.001097.

[12] Johnson, S.G.; Joannopoulos, J.D. Block-Iterative Frequency-Domain Methods for Maxwell's Equations in a Planewave Basis. *Opt. Express* **2001**, *8*, 173–190. https://doi.org/10.1364/OE.8.000173.

[13] Zak, J. Berry's Phase for Energy Bands in Solids. *Phys. Rev. Lett.* **1989**, *62*, 2747–2750. https://doi.org/10.1103/PhysRevLett.62.2747.

[14] Xiao, M.; Zhang, Z.Q.; Chan, C.T. Surface Impedance and Bulk Band Geometric Phases in One-Dimensional Systems. *Phys. Rev. X* **2014**, *4*, 021017. https://doi.org/10.1103/PhysRevX.4.021017.

[15] Gao, W.; Xiao, M.; Chen, B.; Pun, E.Y.B.; Chan, C.T.; Tam, W.Y. Controlling Interface States in 1D Photonic Crystals by Tuning Bulk Geometric Phases. *Opt. Lett.* **2017**, *42*, 1500–1503. https://doi.org/10.1364/OL.42.001500.

[16] Regis, R.G.; Shoemaker, C.A. A Stochastic Radial Basis Function Method for the Global Optimization of Expensive Functions. *INFORMS J. Comput.* **2007**, *19*, 497–509. https://doi.org/10.1287/ijoc.1060.0182.

[17] Audet, C.; Dennis, J.E., Jr. Mesh Adaptive Direct Search Algorithms for Constrained Optimization. *SIAM J. Optim.* **2006**, *17*, 188–217. https://doi.org/10.1137/040603371.

[18] Audet, C.; Custódio, A.L.; Dennis, J.E., Jr. Erratum: Mesh Adaptive Direct Search Algorithms for Constrained Optimization. *SIAM J. Optim.* **2008**, *18*, 1501–1503. https://doi.org/10.1137/060671267.

[19] Cheng, R.; Jin, Y.; Olhofer, M.; Sendhoff, B. A Reference Vector Guided Evolutionary

Algorithm for Many-Objective Optimization. *IEEE Trans. Evol. Comput.* **2016**, *20*, 773–791. https://doi.org/10.1109/TEVC.2016.2519378.

[20] Wilson, E.B. Probable Inference, the Law of Succession, and Statistical Inference. *J. Am. Stat. Assoc.* **1927**, *22*, 209–212. https://doi.org/10.1080/01621459.1927.10502953.

[21] The MathWorks, Inc. *MATLAB*, version 26.1.0.3276743 (R2026a) Update 3; The MathWorks, Inc.: Natick, MA, USA, 2026.

[22] Rumpf, R.C. Simple Implementation of Arbitrarily Shaped Total-Field/Scattered-Field Regions in Finite-Difference Frequency-Domain. *Prog. Electromagn. Res. B* **2012**, *36*, 221–248. https://doi.org/10.2528/PIERB11092006.

[23] Dassault Systèmes. *CST Studio Suite*, version 2026.5, release of 18 June 2026 (change 1767070); Dassault Systèmes Deutschland GmbH: Darmstadt, Germany, 2026.

[24] Oskooi, A.F.; Roundy, D.; Ibanescu, M.; Bermel, P.; Joannopoulos, J.D.; Johnson, S.G. MEEP: A Flexible Free-Software Package for Electromagnetic Simulations by the FDTD Method. *Comput. Phys. Commun.* **2010**, *181*, 687–702. https://doi.org/10.1016/j.cpc.2009.11.008.

[25] Baratta, I.A.; Dean, J.P.; Dokken, J.S.; Habera, M.; Hale, J.S.; Richardson, C.N.; Rognes, M.E.; Scroggs, M.W.; Sime, N.; Wells, G.N. DOLFINx: The Next Generation FEniCS Problem Solving Environment. Preprint, Zenodo, 2023. https://doi.org/10.5281/zenodo.10447666.

[26] Langmuir, I. The Adsorption of Gases on Plane Surfaces of Glass, Mica and Platinum. *J. Am. Chem. Soc.* **1918**, *40*, 1361–1403. https://doi.org/10.1021/ja02242a004.

[27] Bland, J.M.; Altman, D.G. Statistical Methods for Assessing Agreement between Two Methods of Clinical Measurement. *Lancet* **1986**, *327*, 307–310. https://doi.org/10.1016/S0140-6736(86)90837-8.

[28] Shah, R.K.; London, A.L. *Laminar Flow Forced Convection in Ducts: A Source Book for Compact Heat Exchanger Analytical Data*; Academic Press: New York, NY, USA, 1978; ISBN 978-0-12-020051-1. https://doi.org/10.1016/C2013-0-06152-X.

[29] Johnson, S.G.; Ibanescu, M.; Skorobogatiy, M.A.; Weisberg, O.; Joannopoulos, J.D.; Fink, Y. Perturbation Theory for Maxwell's Equations with Shifting Material Boundaries. *Phys. Rev. E* **2002**, *65*, 066611. https://doi.org/10.1103/PhysRevE.65.066611.

[30] Maboudian, R.; Howe, R.T. Critical Review: Adhesion in Surface Micromechanical Structures. *J. Vac. Sci. Technol. B* **1997**, *15*, 1–20. https://doi.org/10.1116/1.589247.
